# High diversity of picornaviruses in rats from different continents revealed by deep sequencing

**DOI:** 10.1038/emi.2016.90

**Published:** 2016-08-17

**Authors:** Thomas Arn Hansen, Sarah Mollerup, Nam-phuong Nguyen, Nicole E White, Megan Coghlan, David E Alquezar-Planas, Tejal Joshi, Randi Holm Jensen, Helena Fridholm, Kristín Rós Kjartansdóttir, Tobias Mourier, Tandy Warnow, Graham J Belsham, Michael Bunce, Eske Willerslev, Lars Peter Nielsen, Lasse Vinner, Anders Johannes Hansen

**Affiliations:** 1 Centre for GeoGenetics, Natural History Museum of Denmark, University of Copenhagen, DK-1350 Copenhagen, Denmark; 2 Carl R. Woese Institute for Genomic Biology, The University of Illinois at Urbana—Champaign, Urbana, IL 61801-2302, USA; 3 Trace and Environmental DNA Lab and Australian Wildlife Forensic Services, Curtin University, Perth, Western Australia 6102, Australia; 4 Center for Biological Sequence Analysis, Department of Systems Biology, Technical University of Denmark, Kemitorvet, DK-2800 Kongens Lyngby, Denmark; 5 Virus Research and Development, Statens Serum Institut, DK-2300 Copenhagen, Denmark; 6 Departments of Bioengineering and Computer Science, The University of Illinois at Urbana—Champaign, Urbana, IL 61801-2302, USA; 7 National Veterinary Institute, Technical University of Denmark, Lindholm, DK-4771 Kalvehave, Denmark; 8 Department of Autoimmunology and Biomarkers, Statens Serum Institut, DK-2300 Copenhagen, Denmark

**Keywords:** cardiovirus, metagenomics, picornavirus, *Rattus norvegicus*, sequencing, viral discovery

## Abstract

Outbreaks of zoonotic diseases in humans and livestock are not uncommon, and an important component in containment of such emerging viral diseases is rapid and reliable diagnostics. Such methods are often PCR-based and hence require the availability of sequence data from the pathogen. *Rattus norvegicus* (*R. norvegicus*) is a known reservoir for important zoonotic pathogens. Transmission may be direct via contact with the animal, for example, through exposure to its faecal matter, or indirectly mediated by arthropod vectors. Here we investigated the viral content in rat faecal matter (*n*=29) collected from two continents by analyzing 2.2 billion next-generation sequencing reads derived from both DNA and RNA. Among other virus families, we found sequences from members of the *Picornaviridae* to be abundant in the microbiome of all the samples. Here we describe the diversity of the picornavirus-like contigs including near-full-length genomes closely related to the Boone cardiovirus and Theiler's encephalomyelitis virus. From this study, we conclude that picornaviruses within *R. norvegicus* are more diverse than previously recognized. The virome of *R. norvegicus* should be investigated further to assess the full potential for zoonotic virus transmission.

## INTRODUCTION

Rodents constitute a diverse taxonomic group rich in species. Globally, rodents are considered the natural reservoir of a large proportion of known zoonoses.^1, 2^ The ubiquitous *Rattus norvegicus* (*R. norvegicus*) thrives in close proximity to humans in most urban or rural settings and have notoriously been associated with zoonotic disease transmission of mainly bacterial pathogens (for example, *Y. pestis*, rat bite fever and leptopspirosis).^2^ Zoonotic transmission may be direct through contact with the carrier animal or its faeces or indirectly mediated by arthropod vectors (for example, ticks or fleas). Serological and epidemiological evidence shows that rodents are among the animal reservoirs for important human viruses including hanta virus, arenavirus and cowpox virus.^3^ Furthermore, there are strong indications of encephalomyocarditis virus transmission from rodents to domestic swine.^4, 5^ Zoonotic transmissions of emerging viruses are extremely difficult to predict and control. One important factor in containment of outbreaks of viral diseases is efficient PCR-based diagnostics, as in the case of the severe acute respiratory syndrome-coronavirus^6, 7^ and viral haemorrhagic fevers where early differential diagnostics are important to limit secondary transmission.^8^ However, PCR diagnostics requires knowledge about the sequence of the pathogen genome and its variation. Therefore, it remains important to explore the viromes of animal species that potentially host zoonotic viruses.

Examples such as Influenza A virus subtype H5N1,^9^ or middle east respiratory syndrome coronaviruses,^10^ Nipah- or Hendravirus,^11^ Lassavirus^12^ or recently Ebolavirus^13^ show that important emerging diseases may result from infections by viruses belonging to many different taxonomic classes.

Traditionally, known virus species have been surveyed in rodents by serology, sequence-specific PCR or virus propagation in cell cultures.^14, 15, 16^ The diversity of specific virus families hosted by wild rodents have been assessed in previous studies.^17, 18, 19, 20, 21^ Here we used Illumina (San Diego, CA, USA) and Ion Torrent next-generation sequencing (NGS) platforms to examine faecal samples from *R. norvegicus* collected in several metropolis habitats from different continents. The aim of the study was to explore the virome in *R. norvegicus* faecal matter. Our results show a surprisingly high diversity of picornavirus-like contigs. The results suggest that the virome of *R. norvegicus* is far more diverse than previously thought. Furthermore, the results contribute fundamental knowledge on the zoonotic potential of viruses carried by this abundant rodent species, living in very close proximity to humans.

## MATERIALS AND METHODS

### Collection of rat faecal samples

Faecal samples were collected from urban areas of Malaysia, Hong Kong and Denmark. All Danish samples from wild rats (*n*=20) were collected from four locations within the Copenhagen area: Egedal Municipality (EM), Copenhagen University Hospital (CUH), Botanical Garden of Copenhagen (BGC) and Amager East (AE). In addition, one sample was collected from laboratory rats at the faculty of health and medical sciences (FHMS), University of Copenhagen. Five samples were collected in Kuala Lumpur, Malaysia, one in Kuala Langat, Malaysia and two samples were obtained from Hong Kong, China. The samples collected in Asia were shipped at ambient temperature in Falcon tubes and immediately frozen upon arrival. The samples from Denmark were frozen at –20 °C within 24 h of collection. No animals were harmed during the sample collection.

### Virion enrichment and RNA and DNA extraction

The frozen faecal samples were vortexed vigorously in 800 μL of PBS for 1 min and incubated at room temperature for 30 min. Following the incubation, the samples were re-vortexed vigorously for a minute and then centrifuged at 12 000 *g* for 5 min. The supernatant was split into three aliquots of 160 μL and subsequently passed through 0.22 μM sterile filters at 6000 *g* for 5 min. Each of the three filtrates were nuclease treated using 14 μL Turbo DNase (2U/μL; Ambion, Waltham, MA, USA), 6 μL Baseline ZERO DNase (1 U/μL; Epicentre, Madison, WI, USA), 6 μL RNase Cocktail (Ambion), 8.5 μL sterile water and 20.5 μL 10 × Turbo buffer in a total volume of 205 μL and incubated at 37 °C for 2 h. The three aliquots of enriched virions were pooled and nucleic acid extracted using the QIAamp Viral RNA Mini Kit (Qiagen, Hilden, Germany), followed by the addition of 1 μL RNase Out (Invitrogen, Carlsbad, CA, USA) to the extract. Indexed RNA and DNA libraries were subsequently prepared using ScriptSeq v2 (Epicentre) and Nextera XT DNA Sample Preparation kit (Illumina), respectively, according to the manufacturers' guidelines. All samples from AE and Hong Kong as well as four from CUH and two from Kuala Lumpur were pooled location-wise in equal ratios before building ScriptSeq libraries. All samples were sequenced on the HiSeq 2000 with 100 bp long paired-end reads. Eight samples were also sequenced on the MiSeq system with 250 bp long paired-end reads.

### Sequencing data analysis

Raw reads from the HiSeq platform were demultiplexed using Novobarcode (http://novocraft.com/main/index.php, vBeta-0.8). Demultiplexed reads were received from the MiSeq platform. For each sample, AdapterRemoval (v1.1)^22^ was used to trim low-quality bases, to remove adaptor sequences from paired-end reads and to merge paired-end reads overlapping with more than 11 nt. Reads were assembled into larger contigs using Ray Meta (v2.2.0)^23^ with default settings. The contigs are available in NCBI Bioprojects (PRJNA323583). The contigs were mapped using PROmer (v3.07) from the MUMmer package^24^ to several databases from European Bioinformatics Institute (EBI) consisting of archaea, archaeal viruses, bacteria, bacteriophages and viruses. Furthermore, fungi and protist genomes from the National Centre for Biotechnology Information (NCBI) were used for reference. The mapped data were filtered and tiled using delta-filter and delta-tiling from MUMmer, respectively. The contigs were grouped based on how they mapped to the reference. For each group, the mean contig length, mean identity to reference and total coverage of reference were summarized (Supplementary Table S1). The output from PROmer with option show-tiling was used to find all hits from a contig to establish a common taxonomic rank within a group. For instance, if one contig in the group mapped to multiple references, the ranking for the group would be the highest common rank, for example, the kingdom, given by NCBI, as summarized (Supplementary Table S1).

Putative virus contigs were searched against Rfam (version 12.0) from Sanger^25, 26^ to identify potential non-coding RNAs. Multiple structural alignments of internal ribosome entry site (IRES) regions of novel viral contigs and Boone cardiovirus (JQ864242.1) were performed using locARNA-p (v1.7.16).^27^ Secondary structure of the consensus sequence was predicted using partition function and minimum free energy options for RNAalifold (no lonely pairs, no closing G–U pairs).^28^

The reads were mapped to the contigs using Bowtie2 (v2.1.0).^29^ This mapping was used to assess the quality of two Boone cardiovirus-like contigs using samtools (v1.2).^30^ The number of unique reads was calculated using MarkDuplicates from the Picard command-line tools (http://picard.sourceforge.net, v1.130) using the default settings. These unique reads and the contigs were mapped to the Virus Pathogen Database and Analysis Resource (VIPR) database^31^ using Bowtie2 (v2.1.0).^29^

### Phylogenetic analysis

All contigs of >5000 nt in length resembling cardioviruses were extracted and getorf was applied from the Emboss software suite (v6.6.0)^32^ to extract all the open reading frames (ORFs) coding for proteins longer than 100 amino-acid residues. We searched the translated ORFs against the NCBI np database with BLAST^33^ and found four long proteins (ranging from 1818 to 2326 amino-acid residues in length) with significant hits to polyprotein sequences from the *Picornaviridae* family. As previous studies have used the polyprotein sequences to identify novel members of the *Picornaviridae* family,^34, 35, 36^ we selected these four sequences for phylogenetic analysis of the assemblies. In order to infer the evolutionary relationship between our sequences and the members of the *Picornaviridae* family, we searched for homologous polyprotein sequences in the NCBI nr database. Three iterations of PSI-BLAST^37, 38^ were compared against the NCBI nr database using the four sequences as the seed sequences, resulting in 999 hits to *Picornaviridae* sequences, of which 407 were considered near full-length (more than 2000 amino-acid residues long). We aligned the four new sequences and 407 near full-length sequences obtained from the BLAST search using PASTA (v1.1.0),^39^ a new alignment method that can accurately align evolutionarily divergent sequences. We estimated a maximum-likelihood (ML) tree on the alignment under the LFG+gamma substitution model using RAxML (v8.0.19)^40^ (model selection performed using helper scripts on the RAxML website, downloaded on 25 August 2014). Support was drawn on the tree using 100 bootstrap replicate runs (bootstrap values on major nodes were ≥69). The remaining fragmentary contigs were analyzed as follows: hmmbuild from the HMMER software package (version 3.0) [hmmer.org] was used to build a profile Hidden Markov Model (HMM) on the PASTA alignment of the near full-length sequences. Next, hmmsearch was used to find all ORFs from the fragmentary contigs that had a hit to the profile HMM (93 total ORFs hit). We inserted the ORFs into the ML tree using SEPP (SATé-enabled phylogenetic placement) (v2),^41^ a phylogenetic placement method that can accurately place evolutionarily divergent sequences.

### Confirmation of viral contigs

Validation of contigs was carried out using two approaches: specific Sanger sequencing and metagenomics using the Ion torrent personal genome machine (PGM). Specific regions of 18 selected novel viral genomes were validated by Sanger sequencing. Total nucleic acids from samples were extracted using the QIAmp DNA stool kit (Qiagen). Total RNA was reverse transcribed using SuperScript III (Life Technologies, Carlsbad, CA, USA) and random hexamers (50 ng/μL). Specific DNA regions were amplified using primers designed with Primer3 (v3-0.4.0)^42^ (Supplementary Table S3). Amplification was performed using Platinum Taq DNA Polymerase (Life Technologies) with cycling conditions according to the manufacturer and with 40 cycles of amplification. Sanger sequencing was performed with the same amplification primers at Macrogen Europe. TraceTuner (v3.0.6beta)^43^ was used to format AB1 files from SANGER sequencing to fastq using -nocall -q options. Nucleotides with low-quality scores were trimmed using seqtk (https://github.com/lh3/seqtk) trimfq -q 0.01. Sequences were aligned using NUCmer from the MUMMER package. We concurrently performed another metagenomic experiment by reverse transcription and random amplification using primer K as previously described,^44^ with 35 cycles of amplification. These randomly amplified DNA fragments were sequenced on the PGM. All sequences below 50 nt length were discarded and the rest were trimmed of 20 nt from each end to avoid any primer contamination. Next, reads were trimmed and aligned using the same parameters as for the Sanger sequences. Three samples from EM were processed this way.

### Metabarcoding for diet content identification

Total DNA extracted with the Qiagen stool kit was assessed for quantity and quality of the target DNA using quantitative PCR (qPCR). The qPCR assessment allowed identification of low copy-number DNA extracts as well as the presence of inhibitors. Three dilutions (undiluted, 1:10 and 1:100) of each DNA extract were assessed with mammalian 16S rRNA and insect COI mitochondrial primers^45, 46^ in addition to plant trnL plastid primers.^47^ All qPCR assays were carried out in 25 μL volumes, which included: 2 mM MgCl_2_ (Fisher Biotec, Wembley, Australia), 1 × Taq polymerase buffer (Fisher Biotec), 0.4 μM dNTPs (Astral Scientific, Taren Point, Australia), 0.1 mg bovine serum albumin (Fisher Biotec), 0.4 μM of each primer and 0.2 μL of Taq DNA polymerase (AmpliTaq Gold, Applied Biosystems, Foster City, CA, USA). The cycling conditions were: initial denaturation at 95 °C for 5 min, followed by 40 cycles of 95°C for 30 s, annealing at primer-specific temperature for 30 s (16S 57 °C, COI 52 °C, trnL 52 °C), 72 °C for 30 s and a final extension at 72 °C for 10 min (Applied Biosystems).

As described previously,^48, 49^ fusion primers with unique 6–8 bp multiplex identifier (MID) tags were assigned in duplicate to each of the DNA extracts (undiluted, 1:10, or 1:100) that showed positive amplification (free from inhibition) for each of the regions of interest, as identified from the qPCR assessment described above. The fusion primers consisted of adaptor sequences appropriate for the high-throughput sequencing platform (Ion Torrent Life Technologies and MiSeq Illumina), in addition to the gene-specific primer. Fusion MID-tagged PCR amplicons were then purified with Agencourt AMPure XP Beads following the manufacturer's protocol (Beckman Coulter Genomics, MA, USA). To confirm the presence of correct amplicon size and determine crude estimates of concentration, the MID-tagged PCR amplicons were electrophoresed on a 2% agarose gel. Crude equimolar pooling of the final amplicon library was determined from the agarose gel before further quantification and high-throughput sequencing.

Dilution series of the amplicon library were made and quantified against a set of synthetic oligonucleotides of known molarity to determine the appropriate MID-tagged amplicon ratio required for the high-throughput sequencing platforms. Sequencing was carried out following the manufacturer's protocols (Life Technologies and Illumina).

### Analysis of diet content

The high-throughput sequencing output files were retrieved and processed using Geneious (v7.1) (http://www.geneious.com/). Sequence reads were accepted for further processing when they had exact matches for primers and MID tag sequences at both ends of each amplicon. Sequence reads were sorted back into original sample batches by searching for unique MID tags. Non-redundant sequence sets for each sample were generated using USEARCH (v6.2).^50^ Each sample file was then checked for chimeras in USEARCH using the UCHIME *de novo* method,^51^ which were removed along with singleton reads. The files were then imported into YABI,^52^ where a BLASTn search was carried out against the NCBI GenBank nucleotide database.^33, 53^ BLAST files were downloaded and imported into the program MEtaGenome ANalyzer (MEGAN v4.70.4)^54^ for taxonomic analysis using the lowest common ancestor parameters: minimum support of 1, minimum score of 65 and top percent of 5. The taxonomic assignments were based on available databases and represent a conservative estimate of families and genera.

## RESULTS

The introduction of NGS has started a new era for the screening of pathogens carried by vectors like *R. norvegicus*. In this study, we have conducted metagenomic analyses of reads and *de novo* assembled contigs, allowing simultaneous identification of both known and unknown viruses.

Samples from Kuala Lumpur (Malaysia), Kuala Langat (Malaysia), Hong Kong (China) and five locations in the Copenhagen area (Denmark), were subjected to virion-enrichment approaches followed by metagenomic investigations of DNA and RNA. For each sample, sequencing libraries were prepared from both RNA and DNA. Altogether we generated more than 2.2 billion paired-end HiSeq reads and over 32 million paired-end MiSeq reads, from which >15.8 million contigs were generated, using the Ray Meta assembler.

**Figure 1:**
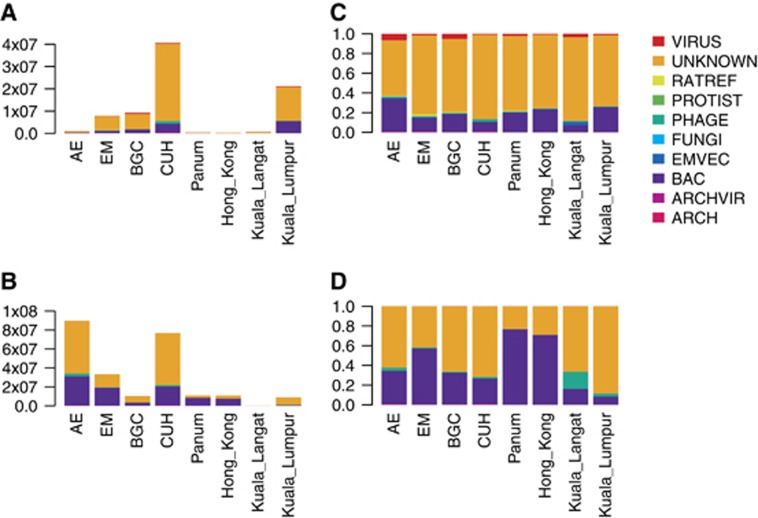
The distributions of unique reads are shown for RNA (**A**) and DNA (**B**) libraries in absolute numbers of unique reads, as well as proportions of unique reads from RNA (**C**) and DNA (**D**) libraries. Unique Illumina reads were mapped to contigs that were mapping to complete reference genomes from viruses, *R. norvegicus* (Rn5), protists, phages, fungi, vectors, bacteria, archaic viruses and Archaea. Sample locations in Denmark are abbreviated as indicated: Amager East (AE), Egedal (EM), Botanical Garden of Copenhagen (BGC), Faculty of Health and Medical Sciences (FHMS) and Copenhagen University Hospital (CUH). Additional sampling locations included Hong Kong, Kuala Langat and Kuala Lumpur.

The contigs were aligned to genomes in a custom database identifying 2524 contigs mapping to known viruses from eukaryotic hosts and ~875 000 contigs with similarity to other species, primarily bacteria. While mapping the contigs to the database can reveal information about the metagenomic diversity, the number of reads constituting these contigs may be used as an estimate of the abundance of the species present in the samples. Consequently, unique reads, mapping back to the contigs, were used to estimate the ratio of the reads classified into taxonomic kingdoms (Figure 1).

More than 320 million unique reads mapped to the contigs. In libraries from each location, an average of ~40 million unique reads mapped to contigs from the RNA libraries, a mean of 1.7% of these reads aligned to eukaryote-specific viruses; this proportion was only 0.015% for the DNA libraries (Figure 1). We found that the greatest proportion of unique reads were either of unknown origin or mapped to bacterial contigs. The number of unique reads varied between the different sampling locations, with CUH and AE yielding the highest number of unique reads from the RNA and DNA libraries, respectively (Figures 1A and 1B). Overall, substantially fewer reads from the RNA libraries could be mapped than from the DNA libraries. Despite this, we found >33 times more reads mapping to contigs resembling viruses of eukaryotes in our RNA library data. Although samples were sequenced to different depths, data sets with fewer reads still exhibited a proportionally larger number of reads mapping to viral contigs, for example, AE (Figures 1C and 1D).

**Figure 2:**
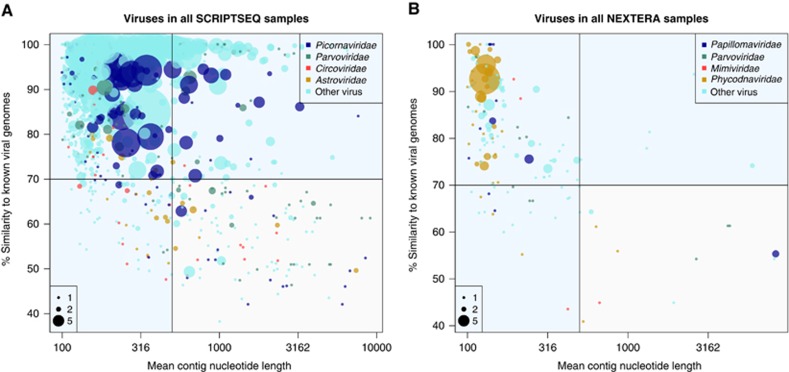
Viral diversity in urban wild *R. norvegicus*. Each dot represents ≥1 contig(s) from a specific sample that maps to a specific reference genome within the virus families indicated (colour coded). The dot size indicates the number of non-overlapping contigs mapping to each reference genome (scale bar). The *x*-axis shows the average contig length, while the *y*-axis shows the mean identity of the contigs mapping to viral reference genomes. Thresholds corresponding to contig lengths exceeding 500 nt and percentage identity <70% are delimited by the horizontal and vertical lines. (**A**) and (**B**) show RNA and DNA sequencing results, respectively.

Contigs were grouped by taxonomic identity to genomic reference sequences and sorted according to the average length and the average identity to the closest related reference genome. All the grouped contigs showing identity to a eukaryote-specific viral reference genome are illustrated in Figure 2.

Combining the RNA and DNA data, a total of 169 contigs in 135 groups had an average length exceeding 500 nt and a mean nucleotide identity <70% to known viral genomes as illustrated in Figure 2 (panels A and B, lower right quadrant) and thus may represent sequences of new viruses.

**Figure 3:**
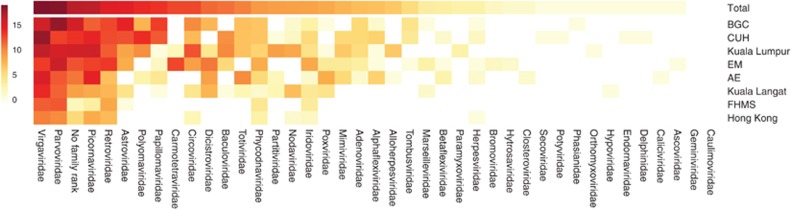
Abundance and diversity of virus families. Distribution and taxonomic classification (family) of reads mapping to assembled viral contigs. Colour intensity represents log2-transformed counts of reads mapped to the contigs resembling a viral family for each sampling location shown on the *y*-axis. Sample locations in Denmark are abbreviated as indicated: Amager East (AE), Egedal (EM), Botanical Garden of Copenhagen (BGC), Faculty of Health and Medical Sciences (FHMS) and Copenhagen University Hospital (CUH).

To obtain an estimate of the relative virus abundance for each sample we mapped all the unique reads onto the virus-associated contigs. The largest proportion of reads mapping to the contigs showed identity to the family *Virgaviridae* (*n*=1 132 052), followed by *Parvoviridae* (*n*=968 808). The third most prevalent group contained reads that were identified as specific viral species but for which there is no taxonomic family classification (*n*=261 382). The fourth and fifth most prevalent families were the *Picornaviridae* (*n*=236 450) and *Retroviridae* (*n*=57 814) (Figure 3). Overall, almost 85% of the virus-associated reads mapped to the contigs from the four mentioned viral families. The samples from Hong Kong and the FHMS contained far fewer reads mapping to the viral contigs (only 0.41% of the reads in total). As the picornavirus family comprises many pathogenic species for humans and livestock, we conducted a more detailed analysis on these sequences.

Our analysis of RNA libraries revealed 17 cases of contigs resembling putative picornaviruses having a mean length of more than 500 nt with <70% identity to published picornavirus genomes. These were found in samples across all localities except Hong Kong, and as expected, the laboratory rats from FHMS. Ten contigs from these cases showed between 85% and 96% identity (via BLASTn alignment) to the sequence of Boone cardiovirus (BCV) (which was not included in our custom database), apparently isolated from laboratory rat faeces. Hence, one contig covered a near-full-length genome (8485 nt) resembling the sequence of the BCV-1 isolate (GenBank JQ864242).

Subsequently, picornavirus-like contigs (excluding the *Kobuvirus* genus) were aligned and placed onto a maximum-likelihood phylogenetic tree of picornavirus reference sequences, using SEPP (see Materials and Methods section).

SEPP analysis showed that 73 contigs across libraries from ten samples resembled Theiler's virus of rats (accession no. BAC58035.1), Rat theilovirus (for example, ACD67870.1) or Theiler's encephalomyelitis virus (for example, AGM61326). The identity of these contigs to the reference sequences ranged from 62% to 100% including a contig spanning 7625 nt with 84% identity to Theiler's encephalomyelitis virus, constituting a novel near-full-length viral genome (Supplementary Table S1). The identified near-full-length genome has a number of indels compared with Theiler's encephalomyelitis virus genomes distributed without disrupting the ORF in each case, while introducing substantial changes in amino-acid residues. This sequence encoded the canonical DVETNPG/P motif at the junction of the 2A/2B protein coding regions.

**Figure 4:**
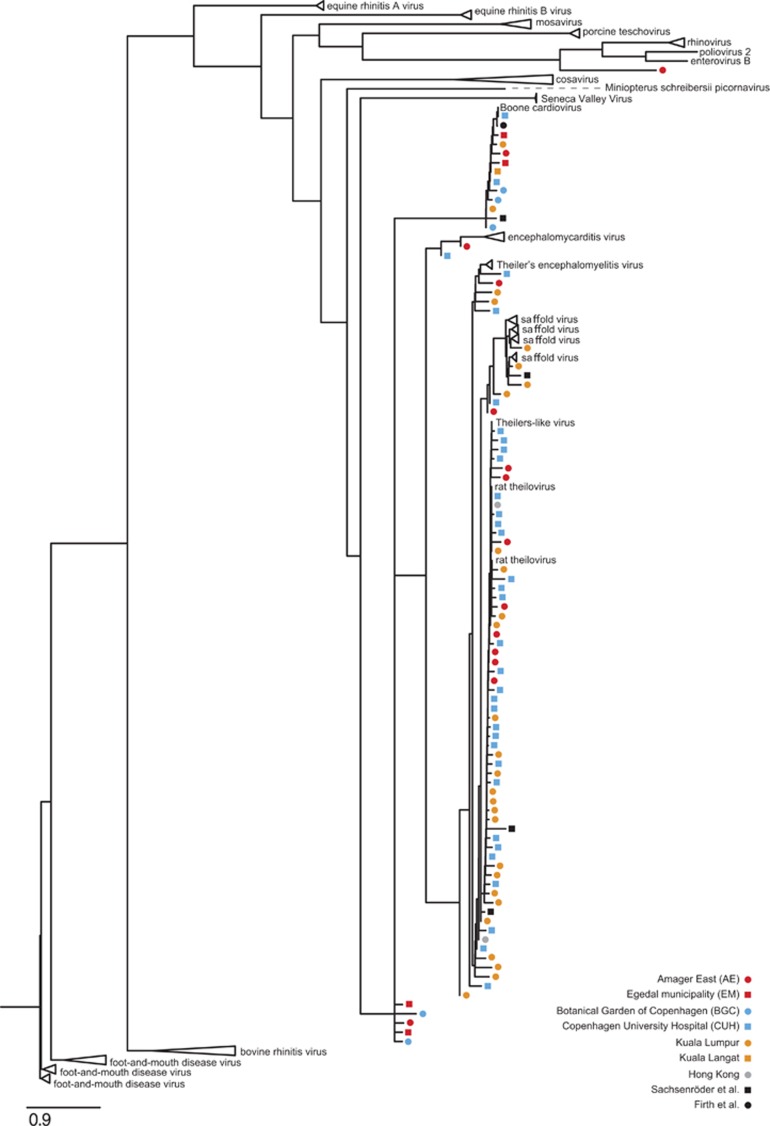
Maximum-likelihood phylogenetic tree of reference sequences from the *Cardiovirus* genus, with subsequently placed picornavirus ORF contigs identified in the present study. Picornavirus-like contigs were added to the tree using SEPP software (see Materials and Methods), including four contigs published elsewhere^19, 20^ (denoted Sachsenröder *et al.* and Firth *et al.*, respectively). Contigs from samples from different geographical locations are colour coded as indicated in the legend. The data set from FHMS was excluded, as no cardiovirus-like contigs were identified. Monophyletic clusters of multiple reference sequences were collapsed (for example, equine rhinitis A virus). The major nodes of the underlying ML tree were supported by bootstrap values ≥69.

In addition, we found distinctive clustering of 11 contigs among the Boone cardiovirus sequences (Figure 4 and Supplementary Materials), including the 8485-nt near-full-length sequence mentioned above. Likewise, two contigs from data published elsewhere also belonged to this monophyletic group. Five contigs (356–2680 nt) coding for ORFs clustered basal to the Boone cardiovirus cluster and the larger cardio-/Saffold virus cluster that comprised a total of 87 contig sequences. Four contigs, including one contig from elsewhere,^19^ formed a monophyletic cluster with the Saffold virus reference sequences.

Interestingly, the Boone cardiovirus and the other contigs within the cluster (*n*=13) lacked the sequence encoding the DVETNPG/P motif. As this motif is required for separation of the viral 2A and 2B proteins, this finding was somewhat surprising. To ensure that the lack of cleavage motif was not due to an assembly error, we mapped the reads onto two Boone cardiovirus-like contigs exceeding >6.5 kb, and found that both showed high depth of coverage (mean >280) that corroborates the findings. We found a total of five ambiguous nucleotide sites in the consensus sequence created from all the reads. None of these ambiguities disrupted the ORF. Furthermore, indels were found in a low fraction of reads (<6.7%), indicating that the contigs may be considered the best assembly from the reads. Hence the lack of DVETPNG/P motif is likely a biological occurrence. The phylogenetic analysis revealed that many of the identified viral contigs originated from novel cardio- or picornaviruses. The results imply that *R. norvegicus* may be a reservoir of picornavirus species far more diverse than the previously recognized.

Outside the ORFs, we conducted consensus RNA secondary structure of the viral IRES sequences in the contigs. The predictions showed that the IRES domain structures were highly conserved among the contigs and the Boone cardiovirus reference sequence, supporting the relationship between these genomes (Supplementary Figure S1).

In the present study, selected viral sequences identified from assembled contigs were reverse transcriptase PCR amplified from the sample extracts and subjected to Sanger sequencing. In addition, we performed alternative metagenomic analyses on data obtained from a different NGS platform (Ion Torrent) (Supplementary Figure S2). In all the investigated cases, we obtained confirmatory results.

In addition to the metagenomic analyses we applied DNA metabarcoding analysis of the faecal samples collected at six locations to assess the dietary composition of the investigated animals. Overall, the metabarcoding analysis suggested a varied composition of plant species in the rat diet. In the six samples, we found evidence of 22 different plant families including species of cereal or vegetables also common in human or household animal food (Supplementary Table S2).

## DISCUSSION

Increased urbanization continues to provide ample opportunities for zoonotic transmission of microbes to humans from rodents, such as the *R. norvegicus.*^2^ As *R. norvegicus* are found in high numbers in most populated areas it is important to investigate their virome. The introduction of NGS has started a new era for pathogen discovery and here we have performed more extensive sequencing than previous studies of faecal samples from *R. norvegicus* enriched for virions.^17, 19, 20^

We used the number unique reads mapping to contigs to estimate the proportions of sequences belonging to different taxonomic ranks. In our study, the possible bias that may be generated by bacterial or fungal growth during post-sampling storage would reduce the proportion of viral reads, hence providing a conservative estimate of the viral proportion. The low virome abundance and diversity in the sample from Hong Kong (Figures 1 and 3) may indicate suboptimal sample preservation and degradation of viral RNA. In agreement with other studies, we found a large proportion of unknown sequences.^55^

In our search for novel genomes, we have focused on contigs exceeding an arbitrarily defined threshold of >500 nt in length and <70% nucleotide identity to known viral reference genomes. It is highly likely that applying less-stringent thresholds would enable identification of even more candidate viral species. For example, we also identified contigs with >70% identity to known rat-specific cardioviruses (Supplementary Table S1). Expanding the analysis to other viral families could reveal further virome diversity.

Likewise, the SEPP approach enabled placement of short contigs (>100 nt) into the picornavirus phylogeny.^41^ Almost all of the small contigs aligned with high confidence (HMMER *e*-value <10^−5^). In virus discovery studies, where full-length novel genomes are rarely detected, identification may rely on small contigs and single reads.^19^ Our results demonstrate the use of this placement approach as a supplement to virus discovery.

We detected picornavirus contigs in RNA libraries from all samples. In ten cases across geographical locations including several urban and rural habitats within Denmark along with Asian metropolises, contigs showed high sequence similarity to Boone cardiovirus. The *Picornaviridae* are a diverse family of ssRNA viruses, which includes numerous human and animal pathogens^56^ that are often shed in faeces.^17, 57, 58^ Among the cardioviruses, Theiler's-like virus of rats and Theiler's encephalomyelitis virus are known to cause demyelination in mice, a condition resembling multiple sclerosis, which, among other symptoms, can lead to paralysis.^59^ The cardioviruses are also associated with respiratory illnesses, severe infections of the myocardium and brain, and sudden infant death syndrome in humans.^60, 61, 62^ The sequence similarity to other known rat (and rodent) picornaviruses and the proportions of identified reads combined, suggest that the identified sequences likely derive from viral species infecting the carrier animal. This suggests a wider presence of cardiovirus species in rats than has previously been recognized. In this regard it is noteworthy that the abundance of picornavirus sequences was relatively high in all samples, and that novel picornavirus species were not restricted to single locations. We found one contig that in SEPP analysis cluster with the clinically relevant rhino, polio and enterovirus B. Although interesting, the sequence was too short (108 bp) to support further sequence analysis. It is also interesting to note that the Boone cardiovirus together with the new rat cardioviruses identified here lack the DVETNPG/P motif at the junction of the 2A and 2B proteins. This motif is present in all the previously identified cardioviruses^63^ and is also present within multiple other genera within the picornavirus family (for example, aphthoviruses, teschoviruses, erboviruses) although not all (for example, enteroviruses, hepatoviruses). Thus, it appears that members of the cardiovirus genus can be sub-divided into those that have this motif (to separate the capsid protein precursor from the non-structural proteins) and those that do not. The presence of human pathogens like the Saffold virus could be indicative of zoonotic potential, but further studies are required to investigate this. The data support that rats, among other rodents, are an important vector to consider for transmission of pathogens to human and livestock. Besides picornaviruses, we identified reads from common gastric virus groups. Expectedly among these, Astroviridae and Adenoviridae (including enteric adenoviruses) sequences were abundant (Figure 3). However, considering their environmental stability, it was somewhat surprising that reads mapping to calicivirus were found in relatively small proportions and that rotavirus (reoviridae) was not detected. In our study, the possible bias that may be generated by bacterial or fungal growth during post-sampling storage that would reduce the proportion of viral reads, hence providing a conservative estimate of the viral proportion. We cannot exclude that taxon-specific degradation of virions post sampling may have biased the results. The low virome abundance and diversity in the sample from Hong Kong (Figures 1 and 3) may indicate suboptimal sample preservation and degradation of viral RNA.

The few existing studies on the diet of rats indicate varied food elements.^64, 65^ From our results it remains unclear if the investigated individuals forage on plant species also consumed by humans. Also it cannot be confirmed if the rats foraged on waste, stored human food or both sources.

We have shown that the virome of *R. norvegicus* faecal matter contains a plethora of viral species. Particularly, picornaviruses were found to be more diverse than previously recognized. In combination with their close proximity to humans and invasive behaviour, the potential of *R. norvegicus* for transmission of zoonotic viral diseases is considerable and should be investigated further.
