# Supplementary figures and images for: High diversity of picornaviruses in rats from different continents revealed by deep sequencing
Source: Emerg Microbes Infect. 2016 Aug 17;5(8):e90–. doi: 10.1038/emi.2016.90 (PMC5034103; doi:10.1038/emi.2016.90)

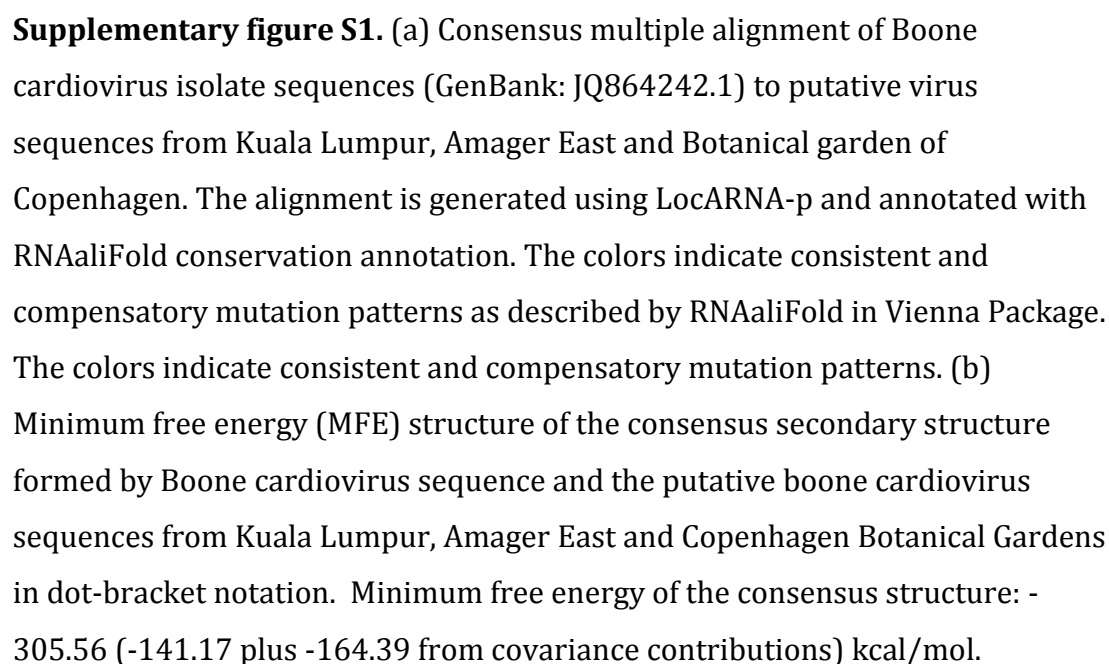

Supplement: Supplementary Figure 1 [file emi201690x1.pdf]
