# Supplementary material for: High diversity of picornaviruses in rats from different continents revealed by deep sequencing
Source: Emerg Microbes Infect. 2016 Aug 17;5(8):e90–. doi: 10.1038/emi.2016.90 (PMC5034103; doi:10.1038/emi.2016.90)
Supplement: Supplementary Figure 2 [file emi201690x2.pdf]

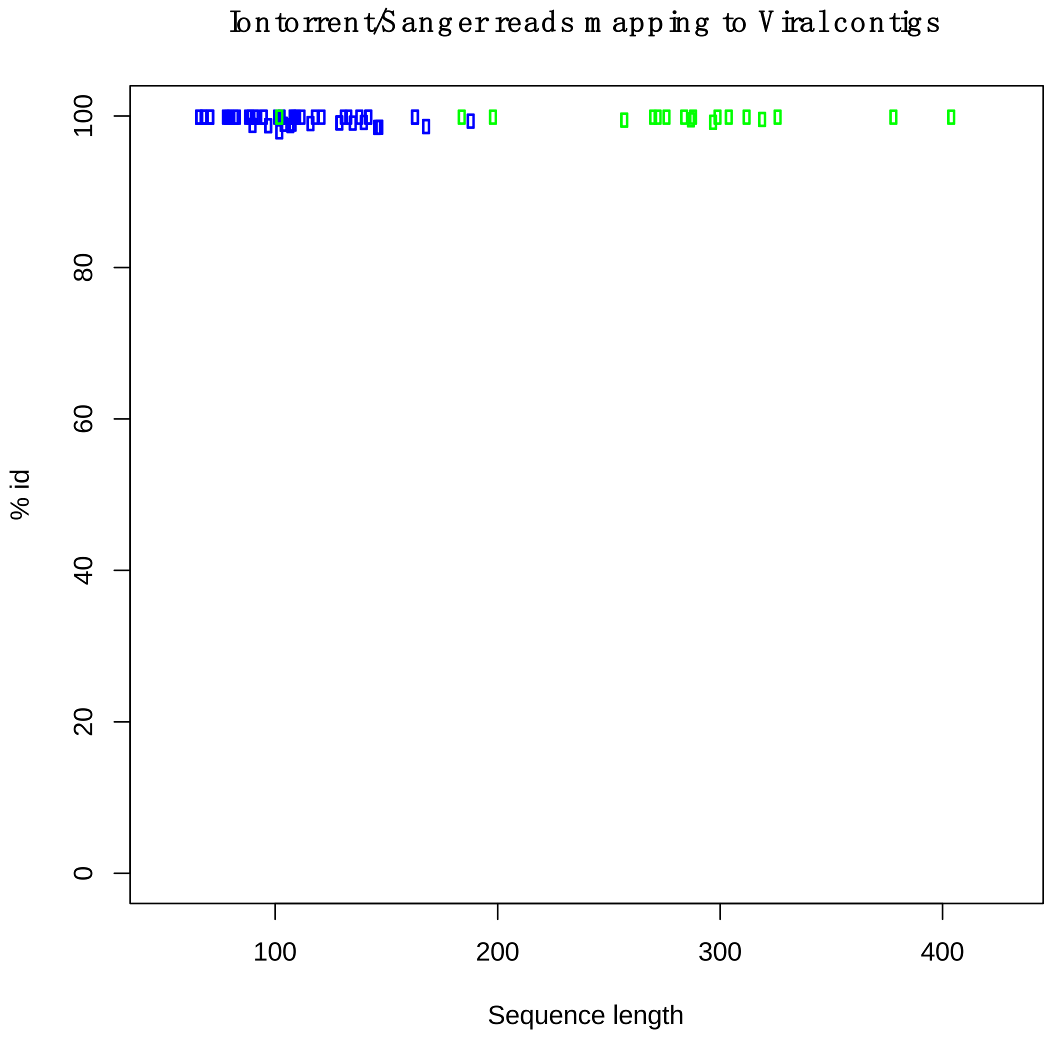

Supplementary figure S2. A subset of the contigs resembling viruses initially generated from Illumina HiSeq data were confirmed using a combination of traditional Sanger sequencing and an IonTorrent viral metagenomic approach with random amplification and sequencing of samples. Blue is IonTorrent sequences and green is Sanger sequences. All Ion Torrent reads mapping to the viral-like contigs had a similarity of 99.66 % and with a standard deviation of 0.53. The Sanger sequenced RT-PCR products showed 99.90 % similarity with contigs, with a standard deviation of 0.23.
