# Supplementary material for: High diversity of picornaviruses in rats from different continents revealed by deep sequencing
Source: Emerg Microbes Infect. 2016 Aug 17;5(8):e90–. doi: 10.1038/emi.2016.90 (PMC5034103; doi:10.1038/emi.2016.90)
Supplement: Supplementary Table 1 [file emi201690x3.pdf]

Supplementary Table S1 A: Virus-like contig found with RNA sequencing

location	Closest ressemblance	mean Per cent i	mean query leng	mean per cent c	Length of	Nur	total Per cent o	Lowest common ancestor
Faculty of Health and Medical Sciences 81	KC977570|KC977570.1 Pandoravirus dulcis, complete genome. [29-AUG-2014]	90.24	147	83.67	1908524	1	0.006444771	Pandoravirus dulcis:species
Faculty of Health and Medical Sciences 81	JQ898342|JQ898342.1 Kobuvirus sewage Kathmandu isolate KoV-SewKTM, complete genome. [29-AUG-201	100	114	100	6939	1	1.642888024	Kobuvirus sewage Kathmandu:no rank
Faculty of Health and Medical Sciences 81	KJ547630|KJ547630.1 Sewage-associated circular DNA virus-6 isolate SaCV-6 NZ-BS4017-2012, complete	90.32	103	90.29	2540	1	3.661417323	Sewage-associated circular DNA virus-6:species
Faculty of Health and Medical Sciences 81	EF101928|EF101928.1 Acanthocystis turfacea Chlorella virus 1, complete genome. [29-AUG-2014]	83.33	126	100	288047	1	0.043742861	Acanthocystis turfacea Chlorella virus 1:species
Faculty of Health and Medical Sciences 81	GQ918152|GQ918152.1 Wiseana iridescent virus, complete genome. [29-AUG-2014]	96.55	117	86.91	205791	2	0.0991297	Wiseana iridescent virus:no rank
Faculty of Health and Medical Sciences 81	KJ938717|KJ938717.1 Caribou feces-associated gemycircularvirus, complete genome. [14-SEP-2014]	80.85	144	97.92	2232	1	6.317204301	Caribou feces-associated gemycircularvirus:species
Faculty of Health and Medical Sciences 81	KF938901|KF938901.1 UNVERIFIED: Anopheles minimus irodovirus isolate AMIV, complete genome. [29-AUG	95	123	97.56	163023	1	0.073609245	Anopheles minimus irodovirus:species
Faculty of Health and Medical Sciences 81	FJ810126|FJ810126.1 Culex pipiens densovirus, complete genome. [29-AUG-2014]	78.79	101	98.02	5759	1	1.719048446	Culex pipiens densovirus:no rank
Faculty of Health and Medical Sciences 81	V01197|V01197.1 Rous sarcoma virus genome, strain Prague C (Pr-C). [29-AUG-2014]	90.77	228	88.732	9312	5	11.08247423	Rous sarcoma virus:species
Faculty of Health and Medical Sciences 81	JF755427|JF755427.1 Mouse kobuvirus M-5/USA/2010, complete genome. [29-AUG-2014]	93.62	142	99.3	8198	1	1.719931691	Mouse kobuvirus M-5/USA/2010:species
Faculty of Health and Medical Sciences 81	M37980|M37980.1 Avian leukosis virus - RSA, complete genome. [29-AUG-2014]	97.22	217	99.54	7286	1	2.964589624	Avian leukosis virus - RSA:no rank
Faculty of Health and Medical Sciences 81	KF170373|KF170373.1 Parvo-like hybrid virus UC1, complete genome. [29-AUG-2014]	100	770	99.74	3638	1	21.11050027	Parvo-like hybrid virus UC1:no rank
Faculty of Health and Medical Sciences 81	HQ425636|HQ425636.1 Avian leukemia virus isolate SCDY1, complete genome. [29-AUG-2014]	91.67	256	98.44	7489	1	3.364935238	Avian leukemia virus:no rank
Faculty of Health and Medical Sciences 81	KF214638|KF214638.1 Parvo-like hybrid virus UC11, complete genome. [29-AUG-2014]	100	583	95.71	3637	1	15.34231509	Parvo-like hybrid virus UC11:no rank
Faculty of Health and Medical Sciences 81	AY859497|AY859497.1 Pepper mild mottle virus isolate PMMoV-CN, complete genome. [29-AUG-2014]	100	119	98.32	6356	1	1.840780365	Pepper mild mottle virus:species
Faculty of Health and Medical Sciences 81	HM029238|HM029238.1 Avian nephritis virus 1 from China, complete genome. [29-AUG-2014]	94.12	153	100	6941	1	2.204293329	Avian nephritis virus 1:no rank
Faculty of Health and Medical Sciences 81	HF920637|HF920637.1 Armadillidium vulgare iridescent virus complete genome [25-JUN-2014]	95.12	128	96.09	220222	1	0.05585273	Armadillidium vulgare iridescent virus:no rank
Faculty of Health and Medical Sciences 81	D12505|D12505.1 Cucumber green mottle mosaic virus RNA, complete genome. [29-AUG-2014]	99.2	6388	99.61	6424	1	99.05043587	Cucumber green mottle mosaic virus:species
Amager East 46-55 v2	JQ898342|JQ898342.1 Kobuvirus sewage Kathmandu isolate KoV-SewKTM, complete genome. [29-AUG-201	94.63769231	203.3076923	98.83230769	6939	13	37.69995677	Kobuvirus sewage Kathmandu:no rank
Amager East 46-55 v2	AF332868|AF332868.1 Tomato mosaic virus isolate Queensland, complete genome. [29-AUG-2014]	99.37666667	2111	99.93666667	6383	3	92.68369105	Tomato mosaic virus:species
Amager East 46-55 v2	KF831027|KF831027.1 Feline kobuvirus strain FK-13, complete genome. [29-AUG-2014]	92.41	239	99.16	8219	1	2.883562477	Feline kobuvirus:no rank
Amager East 46-55 v2	AM491610|AM491610.1 Botryotinia fuckeliana partitivirus 1, complete segment 2, genomic RNA [29-AUG-2	69.23	163	95.71	1566	1	9.961685824	Botryotinia fuckeliana partitivirus 1:species
Amager East 46-55 v2	EF555644|EF555644.1 Human enterovirus 99, complete genome. [29-AUG-2014]	92.955	142	99.2775	7447	4	7.573519538	Human enterovirus C99:no rank
Amager East 46-55 v2	AJ243571|AJ243571.1 Tomato mosaic virus (K1 strain) complete RNA genome [29-AUG-2014]	99.58	726	97.93	6383	1	11.13896287	Tomato mosaic virus:species
Amager East 46-55 v2	AB078435|AB078435.1 Tobacco mild green mosaic virus complete genome, strain:Japanese. [29-AUG-2014	98.074	351.8	98.726	6356	5	27.37570799	Tobacco mild green mosaic virus:species
Amager East 46-55 v2	DQ355023|DQ355023.1 Bell pepper mottle virus, complete genome. [29-AUG-2014]	90.68333333	366.3333333	85.11166667	6375	6	30.4	Bell pepper mottle virus:species
Amager East 46-55 v2	U03387|U03387.1 Turnip vein-clearing virus strain OSU, complete genome. [29-AUG-2014]	92.68	406	99.405	6311	2	12.78719696	Turnip vein-clearing virus:species
Amager East 46-55 v2	KJ938717|KJ938717.1 Caribou feces-associated gemycircularvirus, complete genome. [14-SEP-2014]	86.765	127.5	96.57	2232	2	11.02150538	Caribou feces-associated gemycircularvirus:species
Amager East 46-55 v2	GU244497|GU244497.1 Cafeteria roenbergensis virus BV-PW1, complete genome. [29-AUG-2014]	85.37	127	96.85	617453	1	0.019920545	Cafeteria roenbergensis virus BV-PW1:no rank
Amager East 46-55 v2	KJ196292|KJ196292.1 Norovirus GI/Hu/JP/2007/GI.P3 GI.3/Shimizu/KK2866, complete genome. [11-AUG-	97.5	124	96.77	7745	1	1.549386701	GI.3/Shimizu/KK2866:no rank
Amager East 46-55 v2	AY388617|AY388617.1 Bovine adeno-associated virus, complete genome. [29-AUG-2014]	75.56	270	100	4693	1	5.753249521	Bovine adeno-associated virus:no rank
Amager East 46-55 v2	AB040749|AB040749.1 Aichi virus genomic RNA, complete genome. [29-AUG-2014]	98.04	310	98.71	8280	1	3.695652174	Aichivirus A:species
Amager East 46-55 v2	JN379039|JN379039.1 Salivirus CH, complete genome. [29-AUG-2014]	97.83	140	98.57	7798	1	1.769684534	Salivirus CH:no rank
Amager East 46-55 v2	EU082131|EU082131.1 Black raspberry virus F, complete genome. [29-AUG-2014]	48.03	751	99.87	5077	1	15.0088635	Black raspberry virus F:species
Amager East 46-55 v2	EU547739|EU547739.1 Diplodia scrobiculata RNA virus 1, complete genome. [29-AUG-2014]	78.38	122	90.98	5018	1	2.212036668	Diplodia scrobiculata RNA virus 1:species
Amager East 46-55 v2	DQ321720|DQ321720.2 Nora virus, complete genome. [29-AUG-2014]	81.415	147	97.975	11908	2	2.418542157	Nora virus:species
Amager East 46-55 v2	KC580640|KC580640.1 Canine bocavirus 3 isolate UCD, complete genome. [29-AUG-2014]	75	411	99.27	5278	1	7.730200834	Canine bocavirus 3:species
Amager East 46-55 v2	AF014388|AF014388.1 Drosophila C virus strain EB, complete genome. [29-AUG-2014]	85.96866667	193.0666667	97.87733333	9264	15	30.63471503	Drosophila C virus:species
Amager East 46-55 v2	EF198242|EF198242.1 Marine RNA virus JP-B, complete genome. [29-AUG-2014]	66.67	208	99.52	8926	1	2.319067892	Marine RNA virus JP-B:species
Amager East 46-55 v2	JN704610|JN704610.1 Meles meles fecal virus isolate VS4700006, complete genome. [29-AUG-2014]	94.59	113	98.23	2198	1	5.050045496	Meles meles fecal virus:species
Amager East 46-55 v2	V01197|V01197.1 Rous sarcoma virus genome, strain Prague C (Pr-C). [29-AUG-2014]	93.53333333	152.3333333	98.07	9312	3	4.832474227	Rous sarcoma virus:species
Amager East 46-55 v2	JF755427|JF755427.1 Mouse kobuvirus M-5/USA/2010, complete genome. [29-AUG-2014]	84.13	239.6153846	94.72846154	8198	13	36.04537692	Mouse kobuvirus M-5/USA/2010:species
Amager East 46-55 v2	AY509927|AY509927.1 Pepino mosaic virus strain US2, complete genome. [29-AUG-2014]	96.14	257.3333333	98.79166667	6410	6	23.86895476	Pepino mosaic virus:species
Amager East 46-55 v2	AB090161|AB090161.1 Theiler's-like virus of rats NGS910 genomic RNA, complete genome. [29-AUG-2014]	95.58285714	350.5714286	98.41285714	8021	7	30.22067074	Theiler's-like virus of rats:no rank
Amager East 46-55 v2	AY563023|AY563023.1 Duck picornavirus TW90A, complete genome. [29-AUG-2014]	66.67	295	99.66	8289	1	3.691639522	Avian sapelovirus:species
Amager East 46-55 v2	EU376394|EU376394.1 Human TMEV-like cardiovirus, complete genome. [29-AUG-2014]	49.59	6759	41.19	7961	1	35.04584851	Human TMEV-like cardiovirus:no rank
Amager East 46-55 v2	JQ814851|JQ814851.1 Miniopterus schreibersii picornavirus 1, complete genome. [29-AUG-2014]	46.11	2539	81.88	8468	1	24.94095418	Miniopterus schreibersii picornavirus 1:no rank
Amager East 46-55 v2	EU076703|EU076703.3 Citrus tristeza virus isolate B165, complete genome. [29-AUG-2014]	82.98	143	98.6	19247	1	0.732581701	Citrus tristeza virus:species
Amager East 46-55 v2	M20301|M20301.1 Theiler murine encephalomyelitis virus, complete genome. [29-AUG-2014]	83.33	201	98.51	8093	1	2.446558754	Theiler's encephalomyelitis virus:no rank
Amager East 46-55 v2	KJ194505|KJ194505.1 Human cosavirus isolate Cosavirus Amsterdam 1994, complete genome. [29-AUG-20	98.11	162	98.15	7802	1	2.03793899	Human cosavirus:species
Amager East 46-55 v2	GQ401365|GQ401365.1 Ribgrass mosaic virus strain RMV Actinidia-AC isolate M2/A4, complete genome. [2	98.48	199	99.5	6311	1	3.137379179	Ribgrass mosaic virus:species
Amager East 46-55 v2	AB010145|AB010145.1 Aichi virus genomic RNA, complete genome. [29-AUG-2014]	95.6	548	99.64	8251	1	6.617379712	Aichivirus A:species
Amager East 46-55 v2	KJ158169|KJ158169.1 Rosavirus 2 strain GA7403, complete genome. [29-AUG-2014]	62.896	573.2	97.946	8931	5	31.57541149	Rosavirus A2:subspecies
Amager East 46-55 v2	KJ547632|KJ547632.1 Sewage-associated circular DNA virus-8 isolate SaCV-8 NZ-BS4075-2012, complete	84.62	120	97.5	2577	1	4.54016298	Sewage-associated circular DNA virus-8:species
Amager East 46-55 v2	KF214638|KF214638.1 Parvo-like hybrid virus UC11, complete genome. [29-AUG-2014]	98.99	2392	99.83	3637	1	65.65850976	Parvo-like hybrid virus UC11:no rank
Amager East 46-55 v2	JF320811|JF320811.1 Shallot latent virus isolate WA-1, complete genome. [29-AUG-2014]	75.47	163	97.55	8371	1	1.899414646	Shallot latent virus:species
Amager East 46-55 v2	KC610514|KC610514.1 Scheffersomyces segobiensis virus L isolate NRRL Y-11571, complete genome. [29-	51.835	1029.75	96.4925	4590	4	79.1503268	Scheffersomyces segobiensis virus L:species
Amager East 46-55 v2	AB089381|AB089381.1 Paprika mild mottle virus complete genome, strain:Japanese. [29-AUG-2014]	99.51428571	299.5714286	99.62428571	6524	7	32.05088903	Paprika mild mottle virus:species
Amager East 46-55 v2	JQ941880|JQ941880.1 Bovine hungarovirus 1 strain BHUV1/2008/HUN, complete genome. [29-AUG-2014]	70.83	378	95.44	7583	4	19.06897006	Bovine hungarovirus 1:no rank
Amager East 46-55 v2	KF214645|KF214645.1 Parvo-like hybrid virus UC4, complete genome. [29-AUG-2014]	100	523	94.07	3638	1	13.52391424	Parvo-like hybrid virus UC4:no rank
Amager East 46-55 v2	X00925|X00925.1 Poliovirus type 3 leon 12 a1b sequence (P3/Leon 12 a1b) [29-AUG-2014]	100	108	100	7432	1	1.453175457	Human poliovirus 3:no rank
Amager East 46-55 v2	AM491609|AM491609.1 Botryotinia fuckeliana partitivirus 1, complete segment 1, genomic RNA [29-AUG-2	88.68	162	98.15	1793	1	8.867819297	Botryotinia fuckeliana partitivirus 1:species
Amager East 46-55 v2	X56019|X56019.1 Theilers murine encephalomyelitis virus (TMEV) complete genome [29-AUG-2014]	87.18	121	96.69	8101	1	1.44426614	Theiler's encephalomyelitis virus (STRAIN GDVII):no rank
Amager East 46-55 v2	AF254924|AF254924.1 Youcai mosaic virus isolate Shanghai, complete genome. [29-AUG-2014]	86.11	219	98.63	6301	1	3.428027297	Youcai mosaic virus:species
Amager East 46-55 v2	HE818438|HE818438.1 Tobacco mosaic virus isolate Pingtan-1, complete genome [29-AUG-2014]	98.41125	620.125	99.24125	6395	8	64.55043002	Tobacco mosaic virus:species
Amager East 46-55 v2	AY859497|AY859497.1 Pepper mild mottle virus isolate PMMoV-CN, complete genome. [29-AUG-2014]	99.832	1122.6	99.748	6356	5	84.0780365	Pepper mild mottle virus:species
Amager East 46-55 v2	M34077|M34077.1 Tobacco mild green mosaic virus, complete genome. [29-AUG-2014]	100	119	98.32	6355	1	1.841070024	Tobacco mild green mosaic virus:species
Amager East 46-55 v2	EF428566|EF428566.1 Solenopsis invicta virus 2, complete genome. [29-AUG-2014]	71.7	166	95.78	11319	1	1.404717731	Solenopsis invicta virus 2:species
Amager East 46-55 v2	JF755402|JF755402.1 Rodent stool-associated circular genome virus strain RodSCV M-89, complete genome	53.93	970	85.67	2069	1	40.30932818	Rodent stool-associated circular genome virus:species
Amager East 46-55 v2	EU815052|EU815052.1 Rat theilovirus 1, complete genome. [29-AUG-2014]	98.2	389.6666667	99.05666667	7996	3	14.48224112	Rat theilovirus 1:no rank
Amager East 46-55 v2	KJ547633|KJ547633.1 Sewage-associated circular DNA virus-9 isolate SaCV-9 NZ-BS3681-2012, complete	71.28	2486	34.03	2423	1	33.42963269	Sewage-associated circular DNA virus-9:species
Amager East 46-55 v2	AB017037|AB017037.1 Himetobi P virus genomic RNA, complete sequence. [29-AUG-2014]	44.44	2358	94.78	9275	1	23.80592992	Himetobi P virus:species
Amager East 46-55 v2	X06728|X06728.1 Potexvirus white clover mosaic virus (WC1MV) RNA genome [29-AUG-2014]	98.81	122.5	97.94	5845	2	4.106073567	White clover mosaic virus:species
Amager East 46-55 v2	U01060|U01060.1 Saccharomyces cerevisiae virus La, complete genome. [29-AUG-2014]	50.52	2017	99.5	4615	1	44.00866739	Saccharomyces cerevisiae virus L-BC (La):species
Amager East 46-55 v2	AB628188|AB628188.1 Rehmannia mosaic virus genomic RNA, complete genome, isolate: Japanese. [29-AU	93.94	102	97.06	6395	1	1.548084441	Rehmannia mosaic virus:species
Amager East 46-55 v2	HF920637|HF920637.1 Armadillidium vulgare iridescent virus complete genome [25-JUN-2014]	98.11	161	98.76	220222	1	0.072199871	Armadillidium vulgare iridescent virus:no rank
Amager East 46-55 v2	U16304|U16304.1 Citrus tristeza virus complete genome. [29-AUG-2014]	98.04	154	99.35	19296	1	0.792910448	Citrus tristeza virus:species
Amager East 46-55 v2	S73580|S73580.1 putative polymerase...capsid protein [potato aucuba mosaic potexvirus PAMV, Genomic RI	80.56	113	95.58	7059	1	1.529961751	Potato aucuba mosaic virus:species
Amager East 46-55 v2	D12505|D12505.1 Cucumber green mottle mosaic virus RNA, complete genome. [29-AUG-2014]	99.18	6234	99.76	6424	1	96.80884184	Cucumber green mottle mosaic virus:species
Amager East 46-55 v2	DQ365814|DQ365814.1 Avian leukosis virus strain MQNCSU, complete genome. [29-AUG-2014]	97.66	384	100	7704	1	4.984423676	Avian leukosis virus:species
Amager East 46-55 v2	DQ335246|DQ335246.2 Adeno-associated virus-Go.1, complete genome. [29-AUG-2014]	70.73	243	100	4636	1	5.176876618	Adeno-associated virus-Go.1:no rank
Amager East 46-55 v2	AJ292226|AJ292226.1 Garlic latent virus complete genomic RNA, isolate YH1 [29-AUG-2014]	97.675	138.75	98.3275	8363	4	6.528757623	Garlic latent virus:no rank
Amager East 46-55 v2	KF477193|KF477193.1 Tomato mottle mosaic virus isolate MX5, complete genome. [29-AUG-2014]	99.42727273	503.1818182	90.38090909	6398	11	75.38293217	Tomato mottle mosaic virus:species
egedal 21 v2	JQ898342|JQ898342.1 Kobuvirus sewage Kathmandu isolate KoV-SewKTM, complete genome. [29-AUG-201	92.775	104	99.515	6939	2	2.983138781	Kobuvirus sewage Kathmandu:no rank
egedal 21 v2	AF332868|AF332868.1 Tomato mosaic virus isolate Queensland, complete genome. [29-AUG-2014]	98.93666667	258.5	99.64166667	6383	6	23.09258969	Tomato mosaic virus:species
egedal 21 v2	AJ417701|AJ417701.1 Tomato mosaic virus camellia strain, complete genome [29-AUG-2014]	98.63	197.5	99.49	6383	2	6.156979477	Tomato mosaic virus:species
egedal 21 v2	KJ547631|KJ547631.1 Sewage-associated circular DNA virus-7 isolate SaCV-7 NZ-BS3976-2012, complete	74.515	288.5	99.31	2311	2	24.79446127	Sewage-associated circular DNA virus-7:species
egedal 21 v2	EF101928|EF101928.1 Acanthocystis turfacea Chlorella virus 1, complete genome. [29-AUG-2014]	72.0225	301.375	86.9325	288047	8	0.635312987	Acanthocystis turfacea Chlorella virus 1:species
egedal 21 v2	KF371635|KF371635.1 Faecal-associated gemycircularvirus 7, complete genome. [02-OCT-2014]	87.5	123	97.56	2290	1	5.240174672	Faecal-associated gemycircularvirus 7:species
egedal 21 v2	GQ918152|GQ918152.1 Wiseana iridescent virus, complete genome. [29-AUG-2014]	80.79	162	91.484	205791	5	0.345496159	Wiseana iridescent virus:no rank
egedal 21 v2	KF246569|KF246569.1 Fur seal faeces associated circular DNA virus isolate as50, complete genome. [29-AU	47.68	458	98.91	2925	1	15.48717949	Fur seal faeces associated circular DNA virus:species
egedal 21 v2	DQ355023|DQ355023.1 Bell pepper mottle virus, complete genome. [29-AUG-2014]	86.06166667	270.6666667	98.74333333	6375	6	25.50588235	Bell pepper mottle virus:species
egedal 21 v2	U03387|U03387.1 Turnip vein-clearing virus strain OSU, complete genome. [29-AUG-2014]	95.74333333	135.3333333	98.22	6311	3	6.322294407	Turnip vein-clearing virus:species
egedal 21 v2	KJ938717|KJ938717.1 Caribou feces-associated gemycircularvirus, complete genome. [14-SEP-2014]	79.57	688	68.925	2232	2	27.15053763	Caribou feces-associated gemycircularvirus:species
egedal 21 v2	FJ810126|FJ810126.1 Culex pipiens densovirus, complete genome. [29-AUG-2014]	73.81	133	94.74	5759	1	2.18787984	Culex pipiens densovirus:no rank
egedal 21 v2	JN704610|JN704610.1 Meles meles fecal virus isolate VS4700006, complete genome. [29-AUG-2014]	57.01	1749	51.63	2198	1	42.58416742	Meles meles fecal virus:species
egedal 21 v2	JF755427|JF755427.1 Mouse kobuvirus M-5/USA/2010, complete genome. [29-AUG-2014]	90.7	131	98.47	8198	1	1.573554525	Mouse kobuvirus M-5/USA/2010:species
egedal 21 v2	KJ547648|KJ547648.1 McMurdo Ice Shelf pond-associated circular DNA virus-3 isolate alg49-39, complete g	78.305	146	98.605	3120	2	9.230769231	McMurdo Ice Shelf pond-associated circular DNA virus-3:species
egedal 21 v2	EU376394|EU376394.1 Human TMEV-like cardiovirus, complete genome. [29-AUG-2014]	61.86	2214	37.53	7961	1	10.85290793	Human TMEV-like cardiovirus:no rank
egedal 21 v2	M37980|M37980.1 Avian leukosis virus - RSA, complete genome. [29-AUG-2014]	95.88	435.3333333	99.38	7286	3	17.8287126	Avian leukosis virus - RSA:no rank
egedal 21 v2	FN600414|FN600414.1 Ostreococcus tauri virus 2, complete genome [29-AUG-2014]	68	152	98.68	184409	1	0.081340932	Ostreococcus tauri virus 2:species
egedal 21 v2	EU309041|EU309041.1 Orgyia leucostigma NPV isolate CFS-77, complete genome. [29-AUG-2014]	87.88	110	90	156179	1	0.063388804	Orgyia leucostigma NPV:species
egedal 21 v2	HQ425636|HQ425636.1 Avian leukemia virus isolate SCDY1, complete genome. [29-AUG-2014]	98.55	211	98.1	7489	1	2.764053946	Avian leukemia virus:no rank
egedal 21 v2	AB017503|AB017503.1 Wasabi mottle virus genomic RNA, complete genome, isolate: Shizuoka. [29-AUG-20	76.47	162	94.44	6298	1	2.429342648	Wasabi mottle virus:species
egedal 21 v2	AF170722|AF170722.1 Rabbit fibroma virus, complete genome. [29-AUG-2014]	86.05	130	99.23	159857	1	0.080697123	Rabbit fibroma virus:species
egedal 21 v2	AB089381|AB089381.1 Paprika mild mottle virus complete genome, strain:Japanese. [29-AUG-2014]	99.128	582.1	99.379	6524	10	86.08215819	Paprika mild mottle virus:species
egedal 21 v2	KF738874|KF738874.1 Dragonfly larvae associated circular virus-2 isolate DflaCV-2 NZ-PG8-LS, complete ge	64.29	392	96.43	2398	1	15.76313595	Dragonfly larvae associated circular virus-2:species
egedal 21 v2	EU522111|EU522111.1 Musca domestica salivary gland hypertrophy virus, complete genome. [29-AUG-2014	76.09	140	98.57	124279	1	0.111040481	Musca domestica salivary gland hypertrophy virus:no rank
egedal 21 v2	AF254924|AF254924.1 Youcai mosaic virus isolate Shanghai, complete genome. [29-AUG-2014]	86.84	228	100	6301	1	3.618473258	Youcai mosaic virus:species
egedal 21 v2	HE818438|HE818438.1 Tobacco mosaic virus isolate Pingtan-1, complete genome [29-AUG-2014]	96.90142857	310.2857143	99.00571429	6395	7	33.30727131	Tobacco mosaic virus:species
egedal 21 v2	AY859497|AY859497.1 Pepper mild mottle virus isolate PMMoV-CN, complete genome. [29-AUG-2014]	99.78666667	243	99.14166667	6356	6	22.75015733	Pepper mild mottle virus:species
egedal 21 v2	KJ547633|KJ547633.1 Sewage-associated circular DNA virus-9 isolate SaCV-9 NZ-BS3681-2012, complete	90.7	132	97.73	2423	1	5.323978539	Sewage-associated circular DNA virus-9:species
egedal 21 v2	AB017037|AB017037.1 Himetobi P virus genomic RNA, complete sequence. [29-AUG-2014]	49.11	1533	97.65	9275	1	16.20485175	Himetobi P virus:species
egedal 21 v2	DQ174103|DQ174103.1 Atlantic salmon swim bladder sarcoma virus from USA, complete genome. [29-AUG-	78.05	123	100	10305	1	1.193595342	Atlantic salmon swim bladder sarcoma virus:species
egedal 21 v2	HQ335086|HQ335086.1 Mosquito VEM virus SDBVL G, complete genome. [29-AUG-2014]	97.56	125	98.4	2238	1	5.495978552	Mosquito VEM virus SDBVL G:species
egedal 21 v2	KF158713|KF158713.1 Hemileuca sp. nucleopolyhedrovirus, complete genome. [29-AUG-2014]	88.57	115	91.3	140633	1	0.074662419	Hemileuca sp. nucleopolyhedrovirus:species
egedal 21 v2	KF477193|KF477193.1 Tomato mottle mosaic virus isolate MX5, complete genome. [29-AUG-2014]	96.74	138	98.8275	6398	4	8.533916849	Tomato mottle mosaic virus:species
egedal 21 v2	KC977570|KC977570.1 Pandoravirus dulcis, complete genome. [29-AUG-2014]	86.43	106	91.92	1908524	2	0.01021732	Pandoravirus dulcis:species
egedal 21 v2	AY150217|AY150217.1 Ambystoma tigrinum stebbensi virus, complete genome. [29-AUG-2014]	61.35	487	98.56	106332	1	0.459880375	Ambystoma tigrinum stebbensi virus:no rank
egedal 21 v2	AJ243571|AJ243571.1 Tomato mosaic virus (K1 strain) complete RNA genome [29-AUG-2014]	99.08	328	99.7	6383	1	5.122982923	Tomato mosaic virus:species
egedal 21 v2	DQ643392|DQ643392.1 Aedes taeniorhynchus iridescent virus, complete genome. [29-AUG-2014]	74.51	155	98.71	191100	1	0.080062794	Aedes taeniorhynchus iridescent virus:no rank
egedal 21 v2	GU244497|GU244497.1 Cafeteria roenbergensis virus BV-PW1, complete genome. [29-AUG-2014]	70.4775	132	90.435	617453	4	0.078224577	Cafeteria roenbergensis virus BV-PW1:no rank
egedal 21 v2	KF938901|KF938901.1 UNVERIFIED: Anopheles minimus irodovirus isolate AMIV, complete genome. [29-AUG	85.85666667	121.3333333	98.03666667	163023	3	0.220827736	Anopheles minimus irodovirus:species
egedal 21 v2	FR878069|FR878069.1 Tobacco mosaic virus strain Ohio V, complete genome, genomic RNA [29-AUG-2014	100	219.5	98.785	6392	2	6.805381727	Tobacco mosaic virus strain Ohio V:no rank
egedal 21 v2	KC580640|KC580640.1 Canine bocavirus 3 isolate UCD, complete genome. [29-AUG-2014]	67.35	1502	48.93	5278	1	13.92572944	Canine bocavirus 3:species
egedal 21 v2	AF022937|AF022937.1 Rhopalosiphum padi virus complete genome. [29-AUG-2014]	68.705	282	95.615	10011	2	5.424033563	Rhopalosiphum padi virus:species
egedal 21 v2	V01197|V01197.1 Rous sarcoma virus genome, strain Prague C (Pr-C). [29-AUG-2014]	94.085	281.5	96.408	9312	10	27.95317869	Rous sarcoma virus:species
egedal 21 v2	KF371637|KF371637.1 Faecal-associated gemycircularvirus 5, complete genome. [02-OCT-2014]	95.24	130	96.92	2187	1	5.761316872	Faecal-associated gemycircularvirus 5:species
egedal 21 v2	KC991097|KC991097.1 Solenopsis invicta densovirus isolate SiDNV-Arg, complete genome. [29-AUG-2014]	75	158	98.73	5280	1	2.954545455	Solenopsis invicta densovirus:species
egedal 21 v2	KM035759|KM035759.1 Bovine astrovirus CH13, complete genome. [29-AUG-2014]	59.77	256	99.61	6530	1	3.996937213	Bovine astrovirus CH13:species
egedal 21 v2	AY563023|AY563023.1 Duck picornavirus TW90A, complete genome. [29-AUG-2014]	42.15	1522	47.31	8289	1	8.505247919	Avian sapelovirus:species
egedal 21 v2	JQ814851|JQ814851.1 Miniopterus schreibersii picornavirus 1, complete genome. [29-AUG-2014]	47.55	2109	90.9	8468	1	23.02786963	Miniopterus schreibersii picornavirus 1:no rank
egedal 21 v2	GU991616|GU991616.1 Providence virus isolate vFLM1, complete genome. [29-AUG-2014]	50.49	3362	62.73	6155	1	32.99756296	Providence virus:species
egedal 21 v2	CU469068|CU469068.1 Complete DpAV4 genome. [29-AUG-2014]	71.88	124	77.42	119343	1	0.077926648	Diadromus pulchellus ascovirus 4a:species
egedal 21 v2	JQ340310|JQ340310.1 Astrovirus wild boar/WBAstV-1/2011/HUN, complete genome. [29-AUG-2014]	61.375	399	95.36	6707	2	11.31653496	Astrovirus wild boar/WBAstV-1/2011/HUN:species
egedal 21 v2	KF214638|KF214638.1 Parvo-like hybrid virus UC11, complete genome. [29-AUG-2014]	99.855	810.5	98.555	3637	2	43.79983503	Parvo-like hybrid virus UC11:no rank
egedal 21 v2	GQ351275|GQ351275.1 Chimpanzee stool associated circular ssDNA virus isolate GM510, complete genome	68.85	183	100	2589	1	7.068366165	Chimpanzee stool associated circular ssDNA virus:species
egedal 21 v2	JX544744|JX544744.1 Murine astrovirus strain STL 2, complete genome. [29-AUG-2014]	61.54	454	79.565	6827	2	9.623553537	Murine astrovirus:species
egedal 21 v2	GQ365709|GQ365709.1 Sclerotinia sclerotiorum hypovirulence associated DNA virus 1, complete genome. [2	76.36333333	167.6666667	97.22666667	2166	3	22.29916898	Sclerotinia sclerotiorum hypovirulence associated DNA virus 1:species
egedal 21 v2	EF428566|EF428566.1 Solenopsis invicta virus 2, complete genome. [29-AUG-2014]	56.79	243	100	11319	1	2.146832759	Solenopsis invicta virus 2:species
egedal 21 v2	JF411744|JF411744.1 Paramecium bursaria Chlorella virus 1 (PBCV-1), complete genome. [29-AUG-2014]	71.43	131	96.18	330611	1	0.036296433	Paramecium bursaria Chlorella virus 1:species
egedal 21 v2	AB628188|AB628188.1 Rehmannia mosaic virus genomic RNA, complete genome, isolate: Japanese. [29-AU	97.92	146	98.63	6395	1	2.251759187	Rehmannia mosaic virus:species
egedal 21 v2	DQ890022|DQ890022.1 Paramecium bursaria Chlorella virus FR483, complete genome. [29-AUG-2014]	96.68875	118.625	98.91125	321240	8	0.292304819	Paramecium bursaria Chlorella virus FR483:no rank
egedal 21 v2	KJ547622|KJ547622.1 Sewage-associated circular DNA virus-11 isolate SaCV-11 NZ-BS3997-2012, complet	87.18	118	99.15	2336	1	5.008561644	Sewage-associated circular DNA virus-11:species
egedal 21 v2	V01170|V01170.1 Y73 sarcoma virus complete genome, genomic RNA [29-AUG-2014]	91.76	523	48.76	3718	1	6.858526089	Y73 sarcoma virus:species
egedal 21 v2	HF920633|HF920633.1 Invertebrate iridovirus 22 complete genome [29-AUG-2014]	92.8475	129	95.0775	197693	4	0.247353219	Invertebrate iridovirus 22:species
egedal 21 v2	D12505|D12505.1 Cucumber green mottle mosaic virus RNA, complete genome. [29-AUG-2014]	99.29	2131.333333	99.59	6424	3	98.10087173	Cucumber green mottle mosaic virus:species
egedal 21 v2	DQ365814|DQ365814.1 Avian leukosis virus strain MQNCSU, complete genome. [29-AUG-2014]	95.495	576	99.73	7704	2	14.91433022	Avian leukosis virus:species
egedal 21 v2	JX185429|JX185429.1 Dragonfly-associated circular virus 2 isolate FL2-5X-2010, complete genome. [29-AU	83.33	108	100	2236	1	4.830053667	Dragonfly-associated circular virus 2:species
egedal 21 v2	M73260|M73260.1 Mastadenovirus h5 gene, complete genome. [29-AUG-2014]	100	134.3333333	99.07666667	35935	3	1.11033811	Human adenovirus 5:no rank
egedal 21 v2	JF713713|JF713713.1 Porcine astrovirus 4 strain 35/USA, complete genome. [29-AUG-2014]	73.785	506.5	77.57	6639	2	10.84500678	Porcine astrovirus 4:species
egedal 21 v2	JN566124|JN566124.1 Clitoria yellow mottle virus isolate Larrimah, complete genome. [29-AUG-2014]	66.67	157	97.45	6514	1	2.302732576	Clitoria yellow mottle virus:species
egedal 21 v2	DQ335246|DQ335246.2 Adeno-associated virus-Go.1, complete genome. [29-AUG-2014]	61.34	4174	53.83	4636	1	48.27437446	Adeno-associated virus-Go.1:no rank
egedal 23 v2	JQ898342|JQ898342.1 Kobuvirus sewage Kathmandu isolate KoV-SewKTM, complete genome. [29-AUG-201	94.74	115	99.13	6939	1	1.642888024	Kobuvirus sewage Kathmandu:no rank
egedal 23 v2	AF332868|AF332868.1 Tomato mosaic virus isolate Queensland, complete genome. [29-AUG-2014]	99.674	229.4	99.228	6383	5	17.81294062	Tomato mosaic virus:species
egedal 23 v2	AJ417701|AJ417701.1 Tomato mosaic virus camellia strain, complete genome [29-AUG-2014]	98.75	158.5	98.41	6383	2	4.887983707	Tomato mosaic virus:species
egedal 23 v2	KJ547631|KJ547631.1 Sewage-associated circular DNA virus-7 isolate SaCV-7 NZ-BS3976-2012, complete	78.57	176	95.45	2311	1	7.269580268	Sewage-associated circular DNA virus-7:species
egedal 23 v2	EF101928|EF101928.1 Acanthocystis turfacea Chlorella virus 1, complete genome. [29-AUG-2014]	66.2	428	99.53	288047	1	0.146851035	Acanthocystis turfacea Chlorella virus 1:species

egedal 23 v2	JX962719|JX962719.1 Acanthamoeba polyphaga moumouvirus, complete genome. [29-AUG-2014]	83.72	131	98.47	1021348	1	0.012630367	Acanthamoeba polyphaga moumouvirus:no rank
egedal 23 v2	DQ355023|DQ355023.1 Bell pepper mottle virus, complete genome. [29-AUG-2014]	91.72714286	183.5714286	95.10428571	6375	7	19.38823529	Bell pepper mottle virus:species
egedal 23 v2	U03387|U03387.1 Turnip vein-clearing virus strain OSU, complete genome. [29-AUG-2014]	91.11	140	96.43	6311	1	2.139122168	Turnip vein-clearing virus:species
egedal 23 v2	KJ938717|KJ938717.1 Caribou feces-associated gemycircularvirus, complete genome. [14-SEP-2014]	82.63666667	190.8333333	97.99166667	2232	6	47.26702509	Caribou feces-associated gemycircularvirus:species
egedal 23 v2	X13744|X13744.1 Avian Retrovirus IC10 proviral genome [29-AUG-2014]	95.45	134	98.51	4213	1	3.133159269	Avian retrovirus IC10:species
egedal 23 v2	EU678671|EU678671.1 Pseudaletia unipuncta granulovirus strain Hawaiin, complete genome. [29-AUG-2014	100	164	40.24	176677	1	0.037356306	Pseudaletia unipuncta granulovirus:species
egedal 23 v2	JN704610|JN704610.1 Meles meles fecal virus isolate VS4700006, complete genome. [29-AUG-2014]	90.59	377	67.64	2198	1	11.60145587	Meles meles fecal virus:species
egedal 23 v2	KJ547648|KJ547648.1 McMurdo Ice Shelf pond-associated circular DNA virus-3 isolate alg49-39, complete g	72.34	142	99.3	3120	1	4.519230769	McMurdo Ice Shelf pond-associated circular DNA virus-3:species
egedal 23 v2	M81888|M81888.1 Parvovirus LuIII DNA sequence. [29-AUG-2014]	96.97	100	99	5135	1	1.927945472	LuIII virus:no rank
egedal 23 v2	M37980|M37980.1 Avian leukosis virus - RSA, complete genome. [29-AUG-2014]	100	341	57.695	7286	2	5.023332418	Avian leukosis virus - RSA:no rank
egedal 23 v2	AJ863510|AJ863510.1 Potato virus S complete genome, isolate Vltava [29-AUG-2014]	87.18	118	99.15	8464	1	1.382325142	Potato virus S:species
egedal 23 v2	DQ315364|DQ315364.2 Human adenovirus 41 isolate Tak, complete genome. [29-AUG-2014]	100	135	100	34188	1	0.394875395	Human adenovirus 41:no rank
egedal 23 v2	KF170373|KF170373.1 Parvo-like hybrid virus UC1, complete genome. [29-AUG-2014]	97.67	2615	78.36	3638	1	56.40461792	Parvo-like hybrid virus UC1:no rank
egedal 23 v2	GQ404855|GQ404855.1 Cyclovirus NG14, complete genome. [29-AUG-2014]	75.61	127	96.85	1795	1	6.852367688	Cyclovirus NG14:species
egedal 23 v2	KJ547632|KJ547632.1 Sewage-associated circular DNA virus-8 isolate SaCV-8 NZ-BS4075-2012, complete	60.38	161	98.76	2577	1	6.05355064	Sewage-associated circular DNA virus-8:species
egedal 23 v2	AB089381|AB089381.1 Paprika mild mottle virus complete genome, strain:Japanese. [29-AUG-2014]	98.8575	153.75	99.31166667	6524	12	28.09625996	Paprika mild mottle virus:species
egedal 23 v2	KJ859677|KJ859677.1 Penguinpox virus isolate PSan92, complete genome. [29-AUG-2014]	90	121	99.17	306862	1	0.039105526	Penguinpox virus:species
egedal 23 v2	HE818438|HE818438.1 Tobacco mosaic virus isolate Pingtan-1, complete genome [29-AUG-2014]	99.04142857	141.1428571	98.96857143	6395	7	15.07427678	Tobacco mosaic virus:species
egedal 23 v2	AY859497|AY859497.1 Pepper mild mottle virus isolate PMMoV-CN, complete genome. [29-AUG-2014]	100	117.5	99.595	6356	2	3.68156073	Pepper mild mottle virus:species
egedal 23 v2	HF920637|HF920637.1 Armadillidium vulgare iridescent virus complete genome [25-JUN-2014]	85.33	141.6666667	97.65	220222	3	0.187992117	Armadillidium vulgare iridescent virus:no rank
egedal 23 v2	HQ335086|HQ335086.1 Mosquito VEM virus SDBVL G, complete genome. [29-AUG-2014]	97.37	115	99.13	2238	1	5.09383378	Mosquito VEM virus SDBVL G:species
egedal 23 v2	AF166084|AF166084.1 Tobacco rattle virus RNA1 134 kDa protein, 194 kDa protein, 29 kDa protein, and 1	99.546	135.6	99.104	6791	5	8.982476808	Tobacco rattle virus:species
egedal 23 v2	KF477193|KF477193.1 Tomato mottle mosaic virus isolate MX5, complete genome. [29-AUG-2014]	95	181	99.45	6398	1	2.813379181	Tomato mottle mosaic virus:species
egedal 23 v2	AY150217|AY150217.1 Ambystoma tigrinum stebbensi virus, complete genome. [29-AUG-2014]	60.09666667	535.3333333	83.25	106332	3	1.024150773	Ambystoma tigrinum stebbensi virus:no rank
egedal 23 v2	KF792837|KF792837.1 Feline bocavirus 2 isolate POR1, complete genome. [29-AUG-2014]	91.67	111	97.3	5479	1	1.971162621	Feline bocavirus 2:species
egedal 23 v2	AJ243571|AJ243571.1 Tomato mosaic virus (K1 strain) complete RNA genome [29-AUG-2014]	98.1	634	99.84	6383	1	9.916966943	Tomato mosaic virus:species
egedal 23 v2	X00255|X00255.1 Gardner-Rasheed feline sarcoma virus (GR-FeSV) proviral genome coding for P70 gag-fgr p	100	121	99.17	2025	1	5.925925926	Feline sarcoma virus:no rank
egedal 23 v2	GU244497|GU244497.1 Cafeteria roenbergensis virus BV-PW1, complete genome. [29-AUG-2014]	60.56	222	95.95	617453	1	0.034010686	Cafeteria roenbergensis virus BV-PW1:no rank
egedal 23 v2	AY388617|AY388617.1 Bovine adeno-associated virus, complete genome. [29-AUG-2014]	67.94	901	69.26	4693	1	13.36032389	Bovine adeno-associated virus:no rank
egedal 23 v2	HM004429|HM004429.1 Micromonas sp. RCC1109 virus MpV1, complete genome. [29-AUG-2014]	61.11	168	96.43	184095	1	0.087998044	Micromonas sp. RCC1109 virus MpV1:species
egedal 23 v2	KF938901|KF938901.1 UNVERIFIED: Anopheles minimus irodovirus isolate AMIV, complete genome. [29-AUG	78.16	141.4	97.912	163023	5	0.425093392	Anopheles minimus irodovirus:species
egedal 23 v2	JF938082|JF938082.1 Bat circovirus ZS/China/2011 isolate YN-BtCV-5, complete genome. [29-AUG-2014]	94.29	107	98.13	1818	1	5.775577558	Bat circovirus ZS/China/2011:species
egedal 23 v2	KC580640|KC580640.1 Canine bocavirus 3 isolate UCD, complete genome. [29-AUG-2014]	68.48	558	98.92	5278	1	10.45850701	Canine bocavirus 3:species
egedal 23 v2	KF371633|KF371633.1 Faecal-associated gemycircularvirus 9, complete genome. [02-OCT-2014]	85.37	124	99.19	2130	1	5.774647887	Faecal-associated gemycircularvirus 9:species
egedal 23 v2	V01197|V01197.1 Rous sarcoma virus genome, strain Prague C (Pr-C). [29-AUG-2014]	96.645	296.75	98.68	9312	8	25.22551546	Rous sarcoma virus:species
egedal 23 v2	AY509927|AY509927.1 Pepino mosaic virus strain US2, complete genome. [29-AUG-2014]	100	118	96.61	6410	1	1.778471139	Pepino mosaic virus:species
egedal 23 v2	GU991616|GU991616.1 Providence virus isolate vFLM1, complete genome. [29-AUG-2014]	66.22	596.3333333	89.20666667	6155	3	25.24776604	Providence virus:species
egedal 23 v2	KF371640|KF371640.1 Faecal-associated gemycircularvirus 2, complete genome. [02-OCT-2014]	93.88	151	97.35	2175	1	6.75862069	Faecal-associated gemycircularvirus 2:species
egedal 23 v2	KF214638|KF214638.1 Parvo-like hybrid virus UC11, complete genome. [29-AUG-2014]	99.42	1036	99.9	3637	1	28.45751993	Parvo-like hybrid virus UC11:no rank
egedal 23 v2	EU780426|EU780426.1 Spodoptera litura nucleopolyhedrovirus II, complete genome. [29-AUG-2014]	100	141	42.55	148634	1	0.040367614	Spodoptera litura nucleopolyhedrovirus II:species
egedal 23 v2	JF411744|JF411744.1 Paramecium bursaria Chlorella virus 1 (PBCV-1), complete genome. [29-AUG-2014]	70.975	138	98.915	330611	2	0.084389207	Paramecium bursaria Chlorella virus 1:species
egedal 23 v2	DQ890022|DQ890022.1 Paramecium bursaria Chlorella virus FR483, complete genome. [29-AUG-2014]	93.445	121.8333333	90.785	321240	6	0.207321629	Paramecium bursaria Chlorella virus FR483:no rank
egedal 23 v2	KJ547622|KJ547622.1 Sewage-associated circular DNA virus-11 isolate SaCV-11 NZ-BS3997-2012, complet	97.87	143	98.6	2336	1	6.035958904	Sewage-associated circular DNA virus-11:species
egedal 23 v2	HF920633|HF920633.1 Invertebrate iridovirus 22 complete genome [29-AUG-2014]	85	192	93.75	197693	1	0.08953276	Invertebrate iridovirus 22:species
egedal 23 v2	D12505|D12505.1 Cucumber green mottle mosaic virus RNA, complete genome. [29-AUG-2014]	99.565	3342	99.905	6424	2	97.60273973	Cucumber green mottle mosaic virus:species
egedal 23 v2	DQ365814|DQ365814.1 Avian leukosis virus strain MQNCSU, complete genome. [29-AUG-2014]	87.08666667	436.6666667	98.02333333	7704	3	16.58878505	Avian leukosis virus:species
egedal 23 v2	M73260|M73260.1 Mastadenovirus h5 gene, complete genome. [29-AUG-2014]	100	108	99.07	35935	3	0.893279532	Human adenovirus 5:no rank
egedal 23 v2	DQ335246|DQ335246.2 Adeno-associated virus-Go.1, complete genome. [29-AUG-2014]	77.24	231.25	88.455	4636	4	16.04831752	Adeno-associated virus-Go.1:no rank
egedal 23 v2	KJ577815|KJ577815.1 Porcine stool-associated circular virus 7 isolate EP3-D, complete genome. [02-SEP-2(	79.41	104	98.08	2596	1	3.929121726	Porcine stool-associated circular virus 7:species
egedal 24 v2	JQ898342|JQ898342.1 Kobuvirus sewage Kathmandu isolate KoV-SewKTM, complete genome. [29-AUG-201	98.81	118.5	98.65	6939	2	3.372243839	Kobuvirus sewage Kathmandu:no rank
egedal 24 v2	AF332868|AF332868.1 Tomato mosaic virus isolate Queensland, complete genome. [29-AUG-2014]	99.42	222	98.986	6383	5	17.2489425	Tomato mosaic virus:species
egedal 24 v2	AY150217|AY150217.1 Ambystoma tigrinum stebbensi virus, complete genome. [29-AUG-2014]	59.03	303.5	99.32	106332	2	0.564270398	Ambystoma tigrinum stebbensi virus:no rank
egedal 24 v2	X01678|X01678.1 Brome mosaic virus (BMV) RNA2 [29-AUG-2014]	100	130	99.16	2865	2	9.005235602	Brome mosaic virus:species
egedal 24 v2	JN648103|JN648103.1 Canine bocavirus 1 isolate Con-161, complete genome. [29-AUG-2014]	53.74	441	99.32	5413	1	8.14705339	Canine bocavirus 1:no rank
egedal 24 v2	KF738873|KF738873.1 Dragonfly larvae associated circular virus-1 isolate DflaCV-1 NZ-PG11-LD, complete g	75	111	97.3	2668	1	4.047976012	Dragonfly larvae associated circular virus-1:species
egedal 24 v2	EF101928|EF101928.1 Acanthocystis turfacea Chlorella virus 1, complete genome. [29-AUG-2014]	69.01	136.6666667	98.3	288047	3	0.136436068	Acanthocystis turfacea Chlorella virus 1:species
egedal 24 v2	DQ658743|DQ658743.1 Turnip vein-clearing virus, complete genome. [29-AUG-2014]	97.3	113	98.23	6179	1	1.796407186	Turnip vein-clearing virus:species
egedal 24 v2	DQ090005|DQ090005.2 Human papillomavirus type 38b subtype FA125, complete genome. [29-AUG-2014]	100	128	98.44	7400	1	1.702702703	Human papillomavirus type 38b:no rank
egedal 24 v2	GQ918152|GQ918152.1 Wiseana iridescent virus, complete genome. [29-AUG-2014]	81.88	124	98.005	205791	2	0.118080966	Wiseana iridescent virus:no rank
egedal 24 v2	DQ355023|DQ355023.1 Bell pepper mottle virus, complete genome. [29-AUG-2014]	87.874	226.6	98.59	6375	5	17.78823529	Bell pepper mottle virus:species
egedal 24 v2	FJ959081|FJ959081.1 Circovirus-like genome RW-E, complete genome. [29-AUG-2014]	79.59	156	94.23	2782	1	5.283968368	Circovirus-like genome RW-E:species
egedal 24 v2	U03387|U03387.1 Turnip vein-clearing virus strain OSU, complete genome. [29-AUG-2014]	95.10333333	225.6666667	99.67333333	6311	3	10.69561084	Turnip vein-clearing virus:species
egedal 24 v2	KJ938717|KJ938717.1 Caribou feces-associated gemycircularvirus, complete genome. [14-SEP-2014]	74.696	369.8	82.686	2232	5	65.45698925	Caribou feces-associated gemycircularvirus:species
egedal 24 v2	GU244497|GU244497.1 Cafeteria roenbergensis virus BV-PW1, complete genome. [29-AUG-2014]	76.09	142	97.18	617453	1	0.022349879	Cafeteria roenbergensis virus BV-PW1:no rank
egedal 24 v2	AY388617|AY388617.1 Bovine adeno-associated virus, complete genome. [29-AUG-2014]	68.06	1094	58.96	4693	1	13.80779885	Bovine adeno-associated virus:no rank
egedal 24 v2	KJ577819|KJ577819.1 Porcine stool-associated circular virus 6 isolate XP1, complete genome. [02-SEP-201	68.58	1664	53	2603	1	33.76872839	Porcine stool-associated circular virus 6:species
egedal 24 v2	FR878069|FR878069.1 Tobacco mosaic virus strain Ohio V, complete genome, genomic RNA [29-AUG-2014	100	136	97.06	6392	1	2.065081352	Tobacco mosaic virus strain Ohio V:no rank
egedal 24 v2	JF938082|JF938082.1 Bat circovirus ZS/China/2011 isolate YN-BtCV-5, complete genome. [29-AUG-2014]	52.04	728	75.41	1818	1	32.17821782	Bat circovirus ZS/China/2011:species
egedal 24 v2	KC580640|KC580640.1 Canine bocavirus 3 isolate UCD, complete genome. [29-AUG-2014]	65.13	3296	17.75	5278	1	11.08374384	Canine bocavirus 3:species
egedal 24 v2	JN704610|JN704610.1 Meles meles fecal virus isolate VS4700006, complete genome. [29-AUG-2014]	80.43	522	71.22	2198	2	26.06915378	Meles meles fecal virus:species
egedal 24 v2	V01197|V01197.1 Rous sarcoma virus genome, strain Prague C (Pr-C). [29-AUG-2014]	94.81272727	328.3636364	86.86363636	9312	11	29.07001718	Rous sarcoma virus:species
egedal 24 v2	JF755427|JF755427.1 Mouse kobuvirus M-5/USA/2010, complete genome. [29-AUG-2014]	94.44	109	99.08	8198	1	1.317394486	Mouse kobuvirus M-5/USA/2010:species
egedal 24 v2	KJ547648|KJ547648.1 McMurdo Ice Shelf pond-associated circular DNA virus-3 isolate alg49-39, complete g	92.31	127	92.13	3120	1	3.75	McMurdo Ice Shelf pond-associated circular DNA virus-3:species
egedal 24 v2	KC991097|KC991097.1 Solenopsis invicta densovirus isolate SiDNV-Arg, complete genome. [29-AUG-2014]	65.18	380	88.42	5280	1	6.363636364	Solenopsis invicta densovirus:species
egedal 24 v2	JQ037754|JQ037754.1 Artibeus jamaicensis parvovirus 1, complete genome. [29-AUG-2014]	54.25	467	98.29	4595	1	9.858541893	Artibeus jamaicensis parvovirus 1:species
egedal 24 v2	M37980|M37980.1 Avian leukosis virus - RSA, complete genome. [29-AUG-2014]	97.11	239.3333333	99.36333333	7286	3	9.799615701	Avian leukosis virus - RSA:no rank
egedal 24 v2	JQ814851|JQ814851.1 Miniopterus schreibersii picornavirus 1, complete genome. [29-AUG-2014]	46.11	6303	32.98	8468	1	24.94095418	Miniopterus schreibersii picornavirus 1:no rank
egedal 24 v2	GU991616|GU991616.1 Providence virus isolate vFLM1, complete genome. [29-AUG-2014]	68.985	236.5	98.73	6155	2	7.554833469	Providence virus:species
egedal 24 v2	KF170373|KF170373.1 Parvo-like hybrid virus UC1, complete genome. [29-AUG-2014]	97.98	2978	79.58	3638	1	65.22814733	Parvo-like hybrid virus UC1:no rank
egedal 24 v2	JQ340310|JQ340310.1 Astrovirus wild boar/WBAstV-1/2011/HUN, complete genome. [29-AUG-2014]	54.44	299	90.3	6707	1	4.025644849	Astrovirus wild boar/WBAstV-1/2011/HUN:species
egedal 24 v2	HQ425636|HQ425636.1 Avian leukemia virus isolate SCDY1, complete genome. [29-AUG-2014]	100	183	100	7489	1	2.443583923	Avian leukemia virus:no rank
egedal 24 v2	GQ404855|GQ404855.1 Cyclovirus NG14, complete genome. [29-AUG-2014]	75.44	183	93.44	1795	1	9.526462396	Cyclovirus NG14:species
egedal 24 v2	GQ401365|GQ401365.1 Ribgrass mosaic virus strain RMV Actinidia-AC isolate M2/A4, complete genome. [2!	89.905	215	98.645	6311	2	6.75011884	Ribgrass mosaic virus:species
egedal 24 v2	AB017503|AB017503.1 Wasabi mottle virus genomic RNA, complete genome, isolate: Shizuoka. [29-AUG-2(	90.2	156	98.08	6298	1	2.429342648	Wasabi mottle virus:species
egedal 24 v2	KJ547632|KJ547632.1 Sewage-associated circular DNA virus-8 isolate SaCV-8 NZ-BS4075-2012, complete	75	127	94.49	2577	1	4.656577416	Sewage-associated circular DNA virus-8:species
egedal 24 v2	KF214638|KF214638.1 Parvo-like hybrid virus UC11, complete genome. [29-AUG-2014]	89.86	211	98.1	3637	1	5.691503987	Parvo-like hybrid virus UC11:no rank
egedal 24 v2	AB089381|AB089381.1 Paprika mild mottle virus complete genome, strain:Japanese. [29-AUG-2014]	99.0835	183.6	99.1935	6524	20	55.65603924	Paprika mild mottle virus:species
egedal 24 v2	X85215|X85215.3 Carnation Italian ringspot virus genomic sequence for ORF1-5 [29-AUG-2014]	100	122	98.36	4763	1	2.519420533	Carnation Italian ringspot virus:species
egedal 24 v2	EU522111|EU522111.1 Musca domestica salivary gland hypertrophy virus, complete genome. [29-AUG-2014	80.85	144	97.92	124279	1	0.113454405	Musca domestica salivary gland hypertrophy virus:no rank
egedal 24 v2	GQ365709|GQ365709.1 Sclerotinia sclerotiorum hypovirulence associated DNA virus 1, complete genome. [2	87.01	116.5	99.14	2166	2	10.66481994	Sclerotinia sclerotiorum hypovirulence associated DNA virus 1:species
egedal 24 v2	HE818438|HE818438.1 Tobacco mosaic virus isolate Pingtan-1, complete genome [29-AUG-2014]	97.644	156.4	98.352	6395	5	12.00938233	Tobacco mosaic virus:species
egedal 24 v2	AY859497|AY859497.1 Pepper mild mottle virus isolate PMMoV-CN, complete genome. [29-AUG-2014]	99.63833333	125.3333333	98.89166667	6356	6	11.53241032	Pepper mild mottle virus:species
egedal 24 v2	JF755402|JF755402.1 Rodent stool-associated circular genome virus strain RodSCV M-89, complete genome	63.49	684	55.26	2069	1	18.26969551	Rodent stool-associated circular genome virus:species
egedal 24 v2	KJ547633|KJ547633.1 Sewage-associated circular DNA virus-9 isolate SaCV-9 NZ-BS3681-2012, complete	94.44	349	77.36	2423	1	11.1432109	Sewage-associated circular DNA virus-9:species
egedal 24 v2	AB017037|AB017037.1 Himetobi P virus genomic RNA, complete sequence. [29-AUG-2014]	57.96	1021	97.85	9275	1	10.77088949	Himetobi P virus:species
egedal 24 v2	DQ890022|DQ890022.1 Paramecium bursaria Chlorella virus FR483, complete genome. [29-AUG-2014]	99.1	124.8	98.63	321240	5	0.191445648	Paramecium bursaria Chlorella virus FR483:no rank
egedal 24 v2	D12505|D12505.1 Cucumber green mottle mosaic virus RNA, complete genome. [29-AUG-2014]	98.904	1388.8	99.134	6424	5	98.34993773	Cucumber green mottle mosaic virus:species
egedal 24 v2	DQ365814|DQ365814.1 Avian leukosis virus strain MQNCSU, complete genome. [29-AUG-2014]	98.08	471	99.36	7704	1	6.074766355	Avian leukosis virus:species
egedal 24 v2	KJ547625|KJ547625.1 Sewage-associated circular DNA virus-14 isolate SaCV-14 NZ-BS4064-2012, complet	100	100	99	2335	1	4.239828694	Sewage-associated circular DNA virus-14:species
egedal 24 v2	JF713713|JF713713.1 Porcine astrovirus 4 strain 35/USA, complete genome. [29-AUG-2014]	79.91	156.5	99.73	6639	2	4.699502937	Porcine astrovirus 4:species
egedal 24 v2	DQ335246|DQ335246.2 Adeno-associated virus-Go.1, complete genome. [29-AUG-2014]	73.85	723.5	84.665	4636	2	28.08455565	Adeno-associated virus-Go.1:no rank
Botanical Garden of Copenhagen 60s v2	JQ898342|JQ898342.1 Kobuvirus sewage Kathmandu isolate KoV-SewKTM, complete genome. [29-AUG-201	93.88111111	290	97.46444444	6939	9	35.10592304	Kobuvirus sewage Kathmandu:no rank
Botanical Garden of Copenhagen 60s v2	AF332868|AF332868.1 Tomato mosaic virus isolate Queensland, complete genome. [29-AUG-2014]	99.65	5152	99.69	6383	1	80.46373179	Tomato mosaic virus:species
Botanical Garden of Copenhagen 60s v2	EU273816|EU273816.1 Beet curly top Iran virus-[Yazd], complete genome. [29-AUG-2014]	96.15	156	100	2844	1	5.485232068	Beet curly top Iran virus-[Yazd]:no rank
Botanical Garden of Copenhagen 60s v2	JN648103|JN648103.1 Canine bocavirus 1 isolate Con-161, complete genome. [29-AUG-2014]	85.45	165	100	5413	1	3.048217255	Canine bocavirus 1:no rank
Botanical Garden of Copenhagen 60s v2	KJ547631|KJ547631.1 Sewage-associated circular DNA virus-7 isolate SaCV-7 NZ-BS3976-2012, complete	68.9	259	97.87	2311	2	21.8087408	Sewage-associated circular DNA virus-7:species
Botanical Garden of Copenhagen 60s v2	U66304|U66304.1 Chicken anemia virus cloned isolate 10, complete genome. [29-AUG-2014]	95.65	142	97.18	2319	1	5.95084088	Chicken anemia virus:species
Botanical Garden of Copenhagen 60s v2	EF101928|EF101928.1 Acanthocystis turfacea Chlorella virus 1, complete genome. [29-AUG-2014]	81.784	118.8	93.116	288047	5	0.19267689	Acanthocystis turfacea Chlorella virus 1:species
Botanical Garden of Copenhagen 60s v2	GQ918152|GQ918152.1 Wiseana iridescent virus, complete genome. [29-AUG-2014]	92.86	143	88.11	205791	1	0.061227167	Wiseana iridescent virus:no rank
Botanical Garden of Copenhagen 60s v2	AM398843|AM398843.1 Spodoptera frugiperda ascovirus 1a complete genome [29-AUG-2014]	62.5	132	68.18	156922	1	0.06117689	Spodoptera frugiperda ascovirus 1a:species
Botanical Garden of Copenhagen 60s v2	AB078435|AB078435.1 Tobacco mild green mosaic virus complete genome, strain:Japanese. [29-AUG-2014	99.1	269	98.456	6356	5	20.81497797	Tobacco mild green mosaic virus:species
Botanical Garden of Copenhagen 60s v2	DQ355023|DQ355023.1 Bell pepper mottle virus, complete genome. [29-AUG-2014]	90.7925	978.375	72.90375	6375	8	84.69019608	Bell pepper mottle virus:species
Botanical Garden of Copenhagen 60s v2	U03387|U03387.1 Turnip vein-clearing virus strain OSU, complete genome. [29-AUG-2014]	100	104	98.08	6311	1	1.616225638	Turnip vein-clearing virus:species
Botanical Garden of Copenhagen 60s v2	KJ938717|KJ938717.1 Caribou feces-associated gemycircularvirus, complete genome. [14-SEP-2014]	72.73333333	1230	58.54	2232	3	87.5	Caribou feces-associated gemycircularvirus:species
Botanical Garden of Copenhagen 60s v2	X13744|X13744.1 Avian Retrovirus IC10 proviral genome [29-AUG-2014]	97.73	135	97.78	4213	1	3.133159269	Avian retrovirus IC10:species
Botanical Garden of Copenhagen 60s v2	KF738875|KF738875.1 Dragonfly larvae associated circular virus-3 isolate DflaCV-3 NZ-PG1-LG, complete ge	100	129	97.67	2249	1	5.602489996	Dragonfly larvae associated circular virus-3:species
Botanical Garden of Copenhagen 60s v2	KJ577819|KJ577819.1 Porcine stool-associated circular virus 6 isolate XP1, complete genome. [02-SEP-201	63.49	2659	34.86	2603	1	35.95850941	Porcine stool-associated circular virus 6:species
Botanical Garden of Copenhagen 60s v2	AF190123|AF190123.1 Tobacco vein-clearing virus, complete genome. [29-AUG-2014]	86.05	131	98.47	7767	1	1.660872924	Tobacco vein clearing virus:species
Botanical Garden of Copenhagen 60s v2	KF791042|KF791042.1 Ustilaginoidea virens RNA virus 3, complete genome. [29-AUG-2014]	43.36	1390	96.47	5075	1	26.66009852	Ustilaginoidea virens RNA virus 3:species
Botanical Garden of Copenhagen 60s v2	AF014388|AF014388.1 Drosophila C virus strain EB, complete genome. [29-AUG-2014]	84.95363636	345.2727273	94.17636364	9264	22	71.66450777	Drosophila C virus:species
Botanical Garden of Copenhagen 60s v2	JN704610|JN704610.1 Meles meles fecal virus isolate VS4700006, complete genome. [29-AUG-2014]	82.52	1543.333333	46.78333333	2198	3	63.5577798	Meles meles fecal virus:species
Botanical Garden of Copenhagen 60s v2	JF755427|JF755427.1 Mouse kobuvirus M-5/USA/2010, complete genome. [29-AUG-2014]	89.56	179.2	97.936	8198	5	10.68553306	Mouse kobuvirus M-5/USA/2010:species
Botanical Garden of Copenhagen 60s v2	KJ547648|KJ547648.1 McMurdo Ice Shelf pond-associated circular DNA virus-3 isolate alg49-39, complete g	75.86	177	98.31	3120	1	5.576923077	McMurdo Ice Shelf pond-associated circular DNA virus-3:species
Botanical Garden of Copenhagen 60s v2	AF395114|AF395114.1 Chicken anemia virus strain BD-3, complete genome. [29-AUG-2014]	100	142	98.55	2298	3	18.2767624	Chicken anemia virus:species
Botanical Garden of Copenhagen 60s v2	HE572591|HE572591.1 Beauveria bassiana RNA virus 1, complete genome [29-AUG-2014]	47.65	1262	99.84	5228	1	23.986228	Beauveria bassiana RNA virus 1:species
Botanical Garden of Copenhagen 60s v2	EU376394|EU376394.1 Human TMEV-like cardiovirus, complete genome. [29-AUG-2014]	70.9	407	98.77	7961	1	5.049616882	Human TMEV-like cardiovirus:no rank
Botanical Garden of Copenhagen 60s v2	M37980|M37980.1 Avian leukosis virus - RSA, complete genome. [29-AUG-2014]	100	231	100	7286	1	3.170463903	Avian leukosis virus - RSA:no rank
Botanical Garden of Copenhagen 60s v2	AF033807|AF033807.1 Mouse mammary tumor virus complete proviral genome. [29-AUG-2014]	77.80666667	151	97.05	8805	3	4.974446337	Mouse mammary tumor virus:species
Botanical Garden of Copenhagen 60s v2	KF170373|KF170373.1 Parvo-like hybrid virus UC1, complete genome. [29-AUG-2014]	100	239	99.16	3638	1	6.514568444	Parvo-like hybrid virus UC1:no rank
Botanical Garden of Copenhagen 60s v2	X02449|X02449.1 Hamster polyomavirus (HapV) genome [29-AUG-2014]	73.60333333	1023.666667	60.15833333	5366	6	59.03838986	Hamster polyomavirus:species
Botanical Garden of Copenhagen 60s v2	HQ425636|HQ425636.1 Avian leukemia virus isolate SCDY1, complete genome. [29-AUG-2014]	100	162.5	98.75	7489	2	4.286286554	Avian leukemia virus:no rank
Botanical Garden of Copenhagen 60s v2	KJ938716|KJ938716.1 Ancient caribou feces associated virus, complete genome. [14-SEP-2014]	86.96	141	97.87	2231	1	6.18556701	Ancient caribou feces associated virus:species
Botanical Garden of Copenhagen 60s v2	GQ404855|GQ404855.1 Cyclovirus NG14, complete genome. [29-AUG-2014]	54.85	1416	96.82	1795	1	78.88579387	Cyclovirus NG14:species
Botanical Garden of Copenhagen 60s v2	AB010145|AB010145.1 Aichi virus genomic RNA, complete genome. [29-AUG-2014]	97.37	115	99.13	8251	1	1.381650709	Aichivirus A:species
Botanical Garden of Copenhagen 60s v2	AB017503|AB017503.1 Wasabi mottle virus genomic RNA, complete genome, isolate: Shizuoka. [29-AUG-2(	97.22	111.5	99.54	6298	2	3.524928549	Wasabi mottle virus:species
Botanical Garden of Copenhagen 60s v2	KJ547632|KJ547632.1 Sewage-associated circular DNA virus-8 isolate SaCV-8 NZ-BS4075-2012, complete	63.68	1378	41.36	2577	1	22.11874272	Sewage-associated circular DNA virus-8:species
Botanical Garden of Copenhagen 60s v2	U01834|U01834.1 Multimammate rat papillomavirus, complete genome. [29-AUG-2014]	84.334	164.6	97.922	7687	5	10.381163	Iotapapillomavirus 1:species
Botanical Garden of Copenhagen 60s v2	AB089381|AB089381.1 Paprika mild mottle virus complete genome, strain:Japanese. [29-AUG-2014]	98.57	6498	99.95	6524	1	99.55548743	Paprika mild mottle virus:species
Botanical Garden of Copenhagen 60s v2	AY037928|AY037928.1 Human endogenous retrovirus K113 complete genome. [29-AUG-2014]	89.63	126	98.83	9472	2	2.628800676	Human endogenous retrovirus K113:no rank
Botanical Garden of Copenhagen 60s v2	EF198241|EF198241.1 Marine RNA virus JP-A, complete genome. [29-AUG-2014]	67.42	267	98.88	9236	1	2.890861845	Marine RNA virus JP-A:species
Botanical Garden of Copenhagen 60s v2	KF738874|KF738874.1 Dragonfly larvae associated circular virus-2 isolate DflaCV-2 NZ-PG8-LS, complete ge	73.9	349	71.355	2398	2	15.8882402	Dragonfly larvae associated circular virus-2:species
Botanical Garden of Copenhagen 60s v2	KC771281|KC771281.1 Human cyclovirus VS5700009, complete genome. [29-AUG-2014]	51.14	259	99.61	1832	1	14.41048035	Human cyclovirus VS5700009:species
Botanical Garden of Copenhagen 60s v2	M23385|M23385.1 Squirrel monkey retrovirus-H, complete genome. [29-AUG-2014]	87.8	125	98.4	8785	1	1.40011383	Squirrel monkey retrovirus-H:no rank
Botanical Garden of Copenhagen 60s v2	HE818438|HE818438.1 Tobacco mosaic virus isolate Pingtan-1, complete genome [29-AUG-2014]	93.504	1044.8	98.122	6395	5	80.0938233	Tobacco mosaic virus:species
Botanical Garden of Copenhagen 60s v2	AY859497|AY859497.1 Pepper mild mottle virus isolate PMMoV-CN, complete genome. [29-AUG-2014]	99.23833333	751.6666667	99.04833333	6356	6	69.08432977	Pepper mild mottle virus:species
Botanical Garden of Copenhagen 60s v2	JQ814852|JQ814852.1 Ia io picornavirus 1, complete genome. [29-AUG-2014]	63	815	99.39	7543	1	10.85774891	Ia io picornavirus 1:species
Botanical Garden of Copenhagen 60s v2	KJ547633|KJ547633.1 Sewage-associated circular DNA virus-9 isolate SaCV-9 NZ-BS3681-2012, complete	49.81	2767	28.3	2423	1	28.84853487	Sewage-associated circular DNA virus-9:species
Botanical Garden of Copenhagen 60s v2	HF920637|HF920637.1 Armadillidium vulgare iridescent virus complete genome [25-JUN-2014]	81.41	155.75	95.68	220222	4	0.272452344	Armadillidium vulgare iridescent virus:no rank
Botanical Garden of Copenhagen 60s v2	JX827169|JX827169.1 Rat parvovirus NTU1, complete genome. [29-AUG-2014]	99.0525	184.125	98.90875	4759	8	30.27947048	Rat parvovirus NTU1:species
Botanical Garden of Copenhagen 60s v2	KJ547625|KJ547625.1 Sewage-associated circular DNA virus-14 isolate SaCV-14 NZ-BS4064-2012, complet	100	125	98.395	2335	2	10.53533191	Sewage-associated circular DNA virus-14:species
Botanical Garden of Copenhagen 60s v2	JF729316|JF729316.1 Rabbit astrovirus TN/2208/2010, complete genome. [29-AUG-2014]	57.02	746	45.84	7353	1	4.610363117	Rabbit astrovirus TN/2208/2010:species
Botanical Garden of Copenhagen 60s v2	KF477193|KF477193.1 Tomato mottle mosaic virus isolate MX5, complete genome. [29-AUG-2014]	99.66	959.1428571	85.28428571	6398	7	89.1216005	Tomato mottle mosaic virus:species
Botanical Garden of Copenhagen 60s v2	KC977570|KC977570.1 Pandoravirus dulcis, complete genome. [29-AUG-2014]	78.79	105	94.29	1908524	1	0.005187255	Pandoravirus dulcis:species
Botanical Garden of Copenhagen 60s v2	HM004432|HM004432.1 Bathycoccus sp. RCC1105 virus BpV1, complete genome. [29-AUG-2014]	89.29	102	82.35	198519	1	0.04231333	Bathycoccus sp. RCC1105 virus BpV1:no rank
Botanical Garden of Copenhagen 60s v2	KF831027|KF831027.1 Feline kobuvirus strain FK-13, complete genome. [29-AUG-2014]	64.37	341	74.78	8219	1	3.139068013	Feline kobuvirus:no rank
Botanical Garden of Copenhagen 60s v2	KF413620|KF413620.1 Hypericum japonicum associated circular DNA virus isolate VNHJ1W, complete genor	60.94	2967	12.54	2200	1	17.45454545	Hypericum japonicum associated circular DNA virus:species
Botanical Garden of Copenhagen 60s v2	HM049560|HM049560.1 Murine adenovirus 2 isolate K87, complete genome. [29-AUG-2014]	91.44636364	131.0909091	98.38727273	35203	11	4.030906457	Murine adenovirus 2:no rank
Botanical Garden of Copenhagen 60s v2	KF738873|KF738873.1 Dragonfly larvae associated circular virus-1 isolate DflaCV-1 NZ-PG11-LD, complete g	58.3	3571	23.44	2668	1	31.14692654	Dragonfly larvae associated circular virus-1:species
Botanical Garden of Copenhagen 60s v2	JQ085285|JQ085285.1 Meles meles circovirus-like virus, complete genome. [29-AUG-2014]	55.34	1364	99.85	2218	1	61.54192967	Meles meles circovirus-like virus:species
Botanical Garden of Copenhagen 60s v2	JX645345|JX645345.1 Rat minute virus isolate NTU2, complete genome. [29-AUG-2014]	95.12	123	97.62	4780	2	5.020920502	Rat minute virus:species

Botanical Garden of Copenhagen 60s v2	DQ643392|DQ643392.1 Aedes taeniorhynchus iridescent virus, complete genome. [29-AUG-2014]	72.22	122	88.52	191100	1	0.056514914	Aedes taeniorhynchus iridescent virus:no rank
Botanical Garden of Copenhagen 60s v2	X00255|X00255.1 Gardner-Rasheed feline sarcoma virus (GR-FeSV) proviral genome coding for P70 gag-fgr p	93.18	135	97.78	2025	1	6.518518519	Feline sarcoma virus:no rank
Botanical Garden of Copenhagen 60s v2	M29671|M29671.1 Melon necrotic spot virus (clones pMNS17A and pMNS01A) p29, replicase (p89), p7A, p7	98.964	248	96.976	4266	5	28.34036568	Melon necrotic spot virus:species
Botanical Garden of Copenhagen 60s v2	FJ959081|FJ959081.1 Circovirus-like genome RW-E, complete genome. [29-AUG-2014]	69.16	1040	30.87	2782	1	11.53846154	Circovirus-like genome RW-E:species
Botanical Garden of Copenhagen 60s v2	GQ222416|GQ222416.1 Orcinus orca endogenous retrovirus, complete genome. [29-AUG-2014]	97.22	109	99.08	8273	1	1.305451469	Orcinus orca:species
Botanical Garden of Copenhagen 60s v2	GU244497|GU244497.1 Cafeteria roenbergensis virus BV-PW1, complete genome. [29-AUG-2014]	71.115	156.5	91.945	617453	2	0.046643226	Cafeteria roenbergensis virus BV-PW1:no rank
Botanical Garden of Copenhagen 60s v2	JX310702|JX310702.1 Gyrovirus 4 strain D137, complete genome. [29-AUG-2014]	100	105	97.14	2034	1	5.014749263	Gyrovirus 4:species
Botanical Garden of Copenhagen 60s v2	U60315|U60315.1 Molluscum contagiosum virus subtype 1, complete genome. [29-AUG-2014]	97.87	142	99.3	190289	1	0.07409782	Molluscum contagiosum virus subtype 1:no rank
Botanical Garden of Copenhagen 60s v2	HM004429|HM004429.1 Micromonas sp. RCC1109 virus MpV1, complete genome. [29-AUG-2014]	79.49	118	99.15	184095	1	0.063554143	Micromonas sp. RCC1109 virus MpV1:species
Botanical Garden of Copenhagen 60s v2	KF938901|KF938901.1 UNVERIFIED: Anopheles minimus irodovirus isolate AMIV, complete genome. [29-AUG	86.485	112.5	98.67	163023	2	0.136177104	Anopheles minimus irodovirus:species
Botanical Garden of Copenhagen 60s v2	AF303741|AF303741.1 Chilo iridescent virus complete genome. [29-AUG-2014]	89.19	115	96.59	212482	2	0.104479438	Invertebrate iridescent virus 6:species
Botanical Garden of Copenhagen 60s v2	FR878069|FR878069.1 Tobacco mosaic virus strain Ohio V, complete genome, genomic RNA [29-AUG-2014	100	131	98.47	6392	1	2.018147685	Tobacco mosaic virus strain Ohio V:no rank
Botanical Garden of Copenhagen 60s v2	JX185418|JX185418.1 Dragonfly cyclicusvirus isolate FL1-NZ37-2010, complete genome. [29-AUG-2014]	50	2338	7.7	1831	1	8.847624249	Dragonfly cyclicusvirus:species
Botanical Garden of Copenhagen 60s v2	KC580640|KC580640.1 Canine bocavirus 3 isolate UCD, complete genome. [29-AUG-2014]	51.02	5093	33.46	5278	1	32.56915498	Canine bocavirus 3:species
Botanical Garden of Copenhagen 60s v2	V01197|V01197.1 Rous sarcoma virus genome, strain Prague C (Pr-C). [29-AUG-2014]	91.60666667	542.5	75.95666667	9312	6	21.89647766	Rous sarcoma virus:species
Botanical Garden of Copenhagen 60s v2	KF371637|KF371637.1 Faecal-associated gemycircularvirus 5, complete genome. [02-OCT-2014]	51.55	2158	26.83	2187	1	26.47462277	Faecal-associated gemycircularvirus 5:species
Botanical Garden of Copenhagen 60s v2	KC991097|KC991097.1 Solenopsis invicta densovirus isolate SiDNV-Arg, complete genome. [29-AUG-2014]	64.29	2781	19.63	5280	1	10.22727273	Solenopsis invicta densovirus:species
Botanical Garden of Copenhagen 60s v2	AY563023|AY563023.1 Duck picornavirus TW90A, complete genome. [29-AUG-2014]	42.15	6045	11.91	8289	1	8.505247919	Avian sapelovirus:species
Botanical Garden of Copenhagen 60s v2	EU030001|EU030001.1 RD114 retrovirus isolate SC3C, complete genome. [29-AUG-2014]	86.67	170	52.94	8406	1	1.070663812	RD114 retrovirus:species
Botanical Garden of Copenhagen 60s v2	JQ814851|JQ814851.1 Miniopterus schreibersii picornavirus 1, complete genome. [29-AUG-2014]	46.25	2680	77.57	8468	1	24.94095418	Miniopterus schreibersii picornavirus 1:no rank
Botanical Garden of Copenhagen 60s v2	GU991616|GU991616.1 Providence virus isolate vFLM1, complete genome. [29-AUG-2014]	100	104	98.08	6155	1	1.657189277	Providence virus:species
Botanical Garden of Copenhagen 60s v2	GQ401365|GQ401365.1 Ribgrass mosaic virus strain RMV Actinidia-AC isolate M2/A4, complete genome. [2	97.56	126	97.62	6311	1	1.948977975	Ribgrass mosaic virus:species
Botanical Garden of Copenhagen 60s v2	KJ158169|KJ158169.1 Rosavirus 2 strain GA7403, complete genome. [29-AUG-2014]	75.93	168	96.43	8931	1	1.813906617	Rosavirus A2:subspecies
Botanical Garden of Copenhagen 60s v2	JQ941880|JQ941880.1 Bovine hungarovirus 1 strain BHUV1/2008/HUN, complete genome. [29-AUG-2014]	81.58	156.6	97.574	7583	5	9.494922854	Bovine hungarovirus 1:no rank
Botanical Garden of Copenhagen 60s v2	JX544744|JX544744.1 Murine astrovirus strain STL 2, complete genome. [29-AUG-2014]	59.765	2338	73.585	6827	2	48.33748352	Murine astrovirus:species
Botanical Garden of Copenhagen 60s v2	GQ365709|GQ365709.1 Sclerotinia sclerotiorum hypovirulence associated DNA virus 1, complete genome. [2	51.24	2291	25.53	2166	1	27.70083102	Sclerotinia sclerotiorum hypovirulence associated DNA virus 1:species
Botanical Garden of Copenhagen 60s v2	GQ180114|GQ180114.1 Rattus norvegicus papillomavirus 1 EES-2009, complete genome. [29-AUG-2014]	100	130	99.23	7378	1	1.748441312	Rattus norvegicus papillomavirus 1 EES-2009:no rank
Botanical Garden of Copenhagen 60s v2	JN572118|JN572118.1 Feline picornavirus strain 661F, complete genome. [29-AUG-2014]	52.07	851	99.06	7420	1	11.40161725	Feline picornavirus:species
Botanical Garden of Copenhagen 60s v2	M34077|M34077.1 Tobacco mild green mosaic virus, complete genome. [29-AUG-2014]	99.52	316	99.68	6355	2	9.913453973	Tobacco mild green mosaic virus:species
Botanical Garden of Copenhagen 60s v2	DQ008355|DQ008355.1 Human herpesvirus 3 strain VariVax, complete genome. [29-AUG-2014]	55.56	130	90	124815	1	0.10575652	Human herpesvirus 3:species
Botanical Garden of Copenhagen 60s v2	JF411744|JF411744.1 Paramecium bursaria Chlorella virus 1 (PBCV-1), complete genome. [29-AUG-2014]	79.63	166	97.59	330611	1	0.049000185	Paramecium bursaria Chlorella virus 1:species
Botanical Garden of Copenhagen 60s v2	JX413107|JX413107.1 Human papillomavirus type 163 isolate KC3, complete genome. [29-AUG-2014]	92.31	121	96.69	7233	1	1.617586064	Human papillomavirus type 163:no rank
Botanical Garden of Copenhagen 60s v2	AF311939|AF311939.1 Human coxsackievirus B4 strain E2 variant, complete genome. [29-AUG-2014]	100	129	100	7397	1	1.74395025	Coxsackievirus B4:no rank
Botanical Garden of Copenhagen 60s v2	DQ890022|DQ890022.1 Paramecium bursaria Chlorella virus FR483, complete genome. [29-AUG-2014]	93.81722222	148.1666667	98.11333333	321240	18	0.812476653	Paramecium bursaria Chlorella virus FR483:no rank
Botanical Garden of Copenhagen 60s v2	KJ547622|KJ547622.1 Sewage-associated circular DNA virus-11 isolate SaCV-11 NZ-BS3997-2012, complet	93.18	134	98.51	2336	1	5.650684932	Sewage-associated circular DNA virus-11:species
Botanical Garden of Copenhagen 60s v2	HF920633|HF920633.1 Invertebrate iridovirus 22 complete genome [29-AUG-2014]	62.79	149	86.58	197693	1	0.063735185	Invertebrate iridovirus 22:species
Botanical Garden of Copenhagen 60s v2	D12505|D12505.1 Cucumber green mottle mosaic virus RNA, complete genome. [29-AUG-2014]	94.63333333	2602.333333	99.94	6424	3	97.63387298	Cucumber green mottle mosaic virus:species
Botanical Garden of Copenhagen 60s v2	DQ365814|DQ365814.1 Avian leukosis virus strain MQNCSU, complete genome. [29-AUG-2014]	95.325	690	99.245	7704	2	17.79595016	Avian leukosis virus:species
Botanical Garden of Copenhagen 60s v2	JX185429|JX185429.1 Dragonfly-associated circular virus 2 isolate FL2-5X-2010, complete genome. [29-AU	56.65	1496	46.52	2236	1	30.59033989	Dragonfly-associated circular virus 2:species
Botanical Garden of Copenhagen 60s v2	DQ335246|DQ335246.2 Adeno-associated virus-Go.1, complete genome. [29-AUG-2014]	61.34	8034	27.97	4636	1	48.27437446	Adeno-associated virus-Go.1:no rank
Botanical Garden of Copenhagen 61s v2	JQ898342|JQ898342.1 Kobuvirus sewage Kathmandu isolate KoV-SewKTM, complete genome. [29-AUG-201	92.7	121.5	97.52	6939	2	3.415477735	Kobuvirus sewage Kathmandu:no rank
Botanical Garden of Copenhagen 61s v2	AF332868|AF332868.1 Tomato mosaic virus isolate Queensland, complete genome. [29-AUG-2014]	100	213	98.355	6383	2	6.579978067	Tomato mosaic virus:species
Botanical Garden of Copenhagen 61s v2	AJ417701|AJ417701.1 Tomato mosaic virus camellia strain, complete genome [29-AUG-2014]	98.86	265	99.62	6383	1	4.135986213	Tomato mosaic virus:species
Botanical Garden of Copenhagen 61s v2	JN648103|JN648103.1 Canine bocavirus 1 isolate Con-161, complete genome. [29-AUG-2014]	65.57	648	98.15	5413	1	11.74949196	Canine bocavirus 1:no rank
Botanical Garden of Copenhagen 61s v2	KJ547631|KJ547631.1 Sewage-associated circular DNA virus-7 isolate SaCV-7 NZ-BS3976-2012, complete	70.11	504	70.46	2311	2	22.71743834	Sewage-associated circular DNA virus-7:species
Botanical Garden of Copenhagen 61s v2	KF371636|KF371636.1 Faecal-associated gemycircularvirus 6, complete genome. [02-OCT-2014]	76	157	95.54	2288	1	6.555944056	Faecal-associated gemycircularvirus 6:species
Botanical Garden of Copenhagen 61s v2	EF101928|EF101928.1 Acanthocystis turfacea Chlorella virus 1, complete genome. [29-AUG-2014]	83.155	137	97.455	288047	2	0.092693206	Acanthocystis turfacea Chlorella virus 1:species
Botanical Garden of Copenhagen 61s v2	GQ918152|GQ918152.1 Wiseana iridescent virus, complete genome. [29-AUG-2014]	74.55	159	98.11	205791	1	0.080178433	Wiseana iridescent virus:no rank
Botanical Garden of Copenhagen 61s v2	DQ355023|DQ355023.1 Bell pepper mottle virus, complete genome. [29-AUG-2014]	88.1	206.3333333	98.14555556	6375	9	28.37647059	Bell pepper mottle virus:species
Botanical Garden of Copenhagen 61s v2	KJ938717|KJ938717.1 Caribou feces-associated gemycircularvirus, complete genome. [14-SEP-2014]	73.44	1126.666667	61.69333333	2232	3	68.5483871	Caribou feces-associated gemycircularvirus:species
Botanical Garden of Copenhagen 61s v2	X13744|X13744.1 Avian Retrovirus IC10 proviral genome [29-AUG-2014]	97.33	452	99.56	4213	1	10.68122478	Avian retrovirus IC10:species
Botanical Garden of Copenhagen 61s v2	KJ577819|KJ577819.1 Porcine stool-associated circular virus 6 isolate XP1, complete genome. [02-SEP-201	86.27	157	97.45	2603	1	5.877833269	Porcine stool-associated circular virus 6:species
Botanical Garden of Copenhagen 61s v2	EU918736|EU918736.1 Human bocavirus 3 strain W471, complete genome. [29-AUG-2014]	100	161	98.76	5242	1	3.033193438	Human bocavirus 3:no rank
Botanical Garden of Copenhagen 61s v2	AF014388|AF014388.1 Drosophila C virus strain EB, complete genome. [29-AUG-2014]	94.87	121	96.69	9264	1	1.262953368	Drosophila C virus:species
Botanical Garden of Copenhagen 61s v2	JN704610|JN704610.1 Meles meles fecal virus isolate VS4700006, complete genome. [29-AUG-2014]	73.78	2253	28.495	2198	2	58.78070974	Meles meles fecal virus:species
Botanical Garden of Copenhagen 61s v2	M81888|M81888.1 Parvovirus Lull DNA sequence. [29-AUG-2014]	93.25	211	99.18	5135	2	8.17916261	LuIII virus:no rank
Botanical Garden of Copenhagen 61s v2	U44978|U44978.1 Porcine parvovirus strain Kresse, complete genome. [29-AUG-2014]	100	131	98.47	4948	1	2.607113985	Porcine parvovirus:no rank
Botanical Garden of Copenhagen 61s v2	EU376394|EU376394.1 Human TMEV-like cardiovirus, complete genome. [29-AUG-2014]	66.46	488	99.59	7961	1	6.067077	Human TMEV-like cardiovirus:no rank
Botanical Garden of Copenhagen 61s v2	M37980|M37980.1 Avian leukosis virus - RSA, complete genome. [29-AUG-2014]	93.90666667	909.6666667	88.94666667	7286	3	35.69860005	Avian leukosis virus - RSA:no rank
Botanical Garden of Copenhagen 61s v2	AF033807|AF033807.1 Mouse mammary tumor virus complete proviral genome. [29-AUG-2014]	79.485	117	97.56	8805	2	2.65758092	Mouse mammary tumor virus:species
Botanical Garden of Copenhagen 61s v2	X02449|X02449.1 Hamster polyomavirus (HapV) genome [29-AUG-2014]	79.575	218.5	90.13333333	5366	6	22.36302646	Hamster polyomavirus:species
Botanical Garden of Copenhagen 61s v2	X01457|X01457.1 Parvovirus h-1, complete genome [29-AUG-2014]	96.44666667	369.3333333	98.81	5176	3	21.09737249	H-1 parvovirus:no rank
Botanical Garden of Copenhagen 61s v2	HQ425636|HQ425636.1 Avian leukemia virus isolate SCDY1, complete genome. [29-AUG-2014]	100	142	99.3	7489	1	1.882761383	Avian leukemia virus:no rank
Botanical Garden of Copenhagen 61s v2	GQ404855|GQ404855.1 Cyclovirus NG14, complete genome. [29-AUG-2014]	52.17	1446	95.85	1795	1	79.72144847	Cyclovirus NG14:species
Botanical Garden of Copenhagen 61s v2	KJ547632|KJ547632.1 Sewage-associated circular DNA virus-8 isolate SaCV-8 NZ-BS4075-2012, complete	61.34	953	84.68	2577	1	31.08265425	Sewage-associated circular DNA virus-8:species
Botanical Garden of Copenhagen 61s v2	U01834|U01834.1 Multimammate rat papillomavirus, complete genome. [29-AUG-2014]	76.51333333	517.1666667	92.66	7687	6	33.60218551	Iotapapillomavirus 1:species
Botanical Garden of Copenhagen 61s v2	AB089381|AB089381.1 Paprika mild mottle virus complete genome, strain:Japanese. [29-AUG-2014]	99.14545455	541.5454545	99.49636364	6524	11	89.3776824	Paprika mild mottle virus:species
Botanical Garden of Copenhagen 61s v2	KF738874|KF738874.1 Dragonfly larvae associated circular virus-2 isolate DflaCV-2 NZ-PG8-LS, complete ge	66.42	1044	75.86	2398	1	32.90241868	Dragonfly larvae associated circular virus-2:species
Botanical Garden of Copenhagen 61s v2	KC771281|KC771281.1 Human cyclovirus VS5700009, complete genome. [29-AUG-2014]	52.78	351	90.6	1832	1	17.68558952	Human cyclovirus VS5700009:species
Botanical Garden of Copenhagen 61s v2	HE818438|HE818438.1 Tobacco mosaic virus isolate Pingtan-1, complete genome [29-AUG-2014]	98.39846154	275.4615385	98.96461538	6395	13	55.12118843	Tobacco mosaic virus:species
Botanical Garden of Copenhagen 61s v2	AY859497|AY859497.1 Pepper mild mottle virus isolate PMMoV-CN, complete genome. [29-AUG-2014]	99.79818182	148.2727273	98.42545455	6356	11	25.25173065	Pepper mild mottle virus:species
Botanical Garden of Copenhagen 61s v2	KJ547633|KJ547633.1 Sewage-associated circular DNA virus-9 isolate SaCV-9 NZ-BS3681-2012, complete	62.03	625	89.76	2423	1	22.41023525	Sewage-associated circular DNA virus-9:species
Botanical Garden of Copenhagen 61s v2	HF920637|HF920637.1 Armadillidium vulgare iridescent virus complete genome [25-JUN-2014]	72.215	153.5	94.795	220222	2	0.132139387	Armadillidium vulgare iridescent virus:no rank
Botanical Garden of Copenhagen 61s v2	JX827169|JX827169.1 Rat parvovirus NTU1, complete genome. [29-AUG-2014]	100	106	99.06	4759	1	2.206345871	Rat parvovirus NTU1:species
Botanical Garden of Copenhagen 61s v2	KJ547625|KJ547625.1 Sewage-associated circular DNA virus-14 isolate SaCV-14 NZ-BS4064-2012, complet	95.56	137	98.54	2335	1	5.781584582	Sewage-associated circular DNA virus-14:species
Botanical Garden of Copenhagen 61s v2	EF028290|EF028290.1 Macaca fascicularis papillomavirus type 1, complete genome. [29-AUG-2014]	84.91	159	100	7588	1	2.095413811	Macaca fascicularis papillomavirus type 1:no rank
Botanical Garden of Copenhagen 61s v2	KF477193|KF477193.1 Tomato mottle mosaic virus isolate MX5, complete genome. [29-AUG-2014]	98.505	182	98.885	6398	2	5.626758362	Tomato mottle mosaic virus:species
Botanical Garden of Copenhagen 61s v2	KF831027|KF831027.1 Feline kobuvirus strain FK-13, complete genome. [29-AUG-2014]	89.19	115	96.52	8219	1	1.350529261	Feline kobuvirus:no rank
Botanical Garden of Copenhagen 61s v2	HM049560|HM049560.1 Murine adenovirus 2 isolate K87, complete genome. [29-AUG-2014]	88.465	124	79.395	35203	2	0.553930063	Murine adenovirus 2:no rank
Botanical Garden of Copenhagen 61s v2	X02396|X02396.1 Black beetle virus (BBV) RNA1 and RNA3 for protein A and B [29-AUG-2014]	100	109	99.08	3106	1	3.477141017	Black beetle virus:species
Botanical Garden of Copenhagen 61s v2	KF738873|KF738873.1 Dragonfly larvae associated circular virus-1 isolate DflaCV-1 NZ-PG11-LD, complete g	58.3	3905	21.43	2668	1	31.14692654	Dragonfly larvae associated circular virus-1:species
Botanical Garden of Copenhagen 61s v2	JQ085285|JQ085285.1 Meles meles circovirus-like virus, complete genome. [29-AUG-2014]	58.04	2312	59.04	2218	1	61.40667268	Meles meles circovirus-like virus:species
Botanical Garden of Copenhagen 61s v2	JQ303225|JQ303225.1 Rhinolophus ferrumequinum retrovirus isolate RfRV, complete genome. [02-JUL-2014	83.64	166	99.4	8389	1	1.966861366	Rhinolophus ferrumequinum retrovirus:no rank
Botanical Garden of Copenhagen 61s v2	JX645345|JX645345.1 Rat minute virus isolate NTU2, complete genome. [29-AUG-2014]	100	180	98.33	4780	1	3.70292887	Rat minute virus:species
Botanical Garden of Copenhagen 61s v2	AJ243571|AJ243571.1 Tomato mosaic virus (K1 strain) complete RNA genome [29-AUG-2014]	100	547	99.82	6383	1	8.553971487	Tomato mosaic virus:species
Botanical Garden of Copenhagen 61s v2	FJ959081|FJ959081.1 Circovirus-like genome RW-E, complete genome. [29-AUG-2014]	70.65	780.5	51.305	2782	2	28.57656362	Circovirus-like genome RW-E:species
Botanical Garden of Copenhagen 61s v2	KF938901|KF938901.1 UNVERIFIED: Anopheles minimus irodovirus isolate AMIV, complete genome. [29-AUG	80.56	108	100	163023	1	0.066248321	Anopheles minimus irodovirus:species
Botanical Garden of Copenhagen 61s v2	AF303741|AF303741.1 Chilo iridescent virus complete genome. [29-AUG-2014]	84.09	134	98.51	212482	1	0.062122909	Invertebrate iridescent virus 6:species
Botanical Garden of Copenhagen 61s v2	AF053745|AF053745.1 Mus dunni endogenous virus complete genome. [29-AUG-2014]	81.58	117	97.44	8655	1	1.317157712	Mus dunni endogenous virus:no rank
Botanical Garden of Copenhagen 61s v2	JF930327|JF930327.1 Pitaya virus X isolate P37, complete genome. [02-JUL-2014]	100	176	98.86	6677	1	2.605960761	Pitaya virus X:species
Botanical Garden of Copenhagen 61s v2	KC580640|KC580640.1 Canine bocavirus 3 isolate UCD, complete genome. [29-AUG-2014]	51.02	3736	45.61	5278	1	32.56915498	Canine bocavirus 3:species
Botanical Garden of Copenhagen 61s v2	V01197|V01197.1 Rous sarcoma virus genome, strain Prague C (Pr-C). [29-AUG-2014]	96.2075	421	78.75	9312	4	10.56701031	Rous sarcoma virus:species
Botanical Garden of Copenhagen 61s v2	KF371637|KF371637.1 Faecal-associated gemycircularvirus 5, complete genome. [02-OCT-2014]	92.2	153	99.125	2187	2	13.85459534	Faecal-associated gemycircularvirus 5:species
Botanical Garden of Copenhagen 61s v2	KC991097|KC991097.1 Solenopsis invicta densovirus isolate SiDNV-Arg, complete genome. [29-AUG-2014]	64.29	5092	10.72	5280	1	10.22727273	Solenopsis invicta densovirus:species
Botanical Garden of Copenhagen 61s v2	AY563023|AY563023.1 Duck picornavirus TW90A, complete genome. [29-AUG-2014]	52.59	356	96.91	8289	1	4.198335143	Avian sapelovirus:species
Botanical Garden of Copenhagen 61s v2	JQ340310|JQ340310.1 Astrovirus wild boar/WBAstV-1/2011/HUN, complete genome. [29-AUG-2014]	66.67	297	96.97	6707	1	4.294021172	Astrovirus wild boar/WBAstV-1/2011/HUN:species
Botanical Garden of Copenhagen 61s v2	JX185430|JX185430.1 Dragonfly-associated circular virus 1 isolate FL1-2X-2010, complete genome. [29-AU	63.87	3410	10.47	2225	1	16.04494382	Dragonfly-associated circular virus 1:species
Botanical Garden of Copenhagen 61s v2	FJ959079|FJ959079.1 Circovirus-like genome RW-C, complete genome. [29-AUG-2014]	62.5	350	63.43	1803	1	13.31114809	Circovirus-like genome RW-C:species
Botanical Garden of Copenhagen 61s v2	JX544744|JX544744.1 Murine astrovirus strain STL 2, complete genome. [29-AUG-2014]	60.63	468.5	83.55	6827	2	11.07367804	Murine astrovirus:species
Botanical Garden of Copenhagen 61s v2	GQ365709|GQ365709.1 Sclerotinia sclerotiorum hypovirulence associated DNA virus 1, complete genome. [2	81.405	401.5	91.135	2166	2	31.99445983	Sclerotinia sclerotiorum hypovirulence associated DNA virus 1:species
Botanical Garden of Copenhagen 61s v2	HQ113105|HQ113105.1 Lausannevirus isolate 7715, complete genome. [29-AUG-2014]	100	121	76.86	346754	1	0.026820166	Lausannevirus:species
Botanical Garden of Copenhagen 61s v2	M34077|M34077.1 Tobacco mild green mosaic virus, complete genome. [29-AUG-2014]	100	111	100	6355	1	1.746656176	Tobacco mild green mosaic virus:species
Botanical Garden of Copenhagen 61s v2	HQ738636|HQ738636.1 Cyclovirus PKgoat11/PAK/2009 isolate PKgoat11, complete genome. [29-AUG-201	60.27	230	95.22	1751	1	12.50713878	Cyclovirus PKgoat11/PAK/2009:species
Botanical Garden of Copenhagen 61s v2	JF411744|JF411744.1 Paramecium bursaria Chlorella virus 1 (PBCV-1), complete genome. [29-AUG-2014]	54.35	143	96.5	330611	1	0.039926076	Paramecium bursaria Chlorella virus 1:species
Botanical Garden of Copenhagen 61s v2	JX413107|JX413107.1 Human papillomavirus type 163 isolate KC3, complete genome. [29-AUG-2014]	91.81666667	124.3333333	97.91333333	7233	3	5.06014102	Human papillomavirus type 163:no rank
Botanical Garden of Copenhagen 61s v2	DQ890022|DQ890022.1 Paramecium bursaria Chlorella virus FR483, complete genome. [29-AUG-2014]	88.63	171	94.78294118	321240	17	0.87224505	Paramecium bursaria Chlorella virus FR483:no rank
Botanical Garden of Copenhagen 61s v2	D12505|D12505.1 Cucumber green mottle mosaic virus RNA, complete genome. [29-AUG-2014]	99.49	1329.8	99.32	6424	5	95.68804483	Cucumber green mottle mosaic virus:species
Botanical Garden of Copenhagen 61s v2	JQ412057|JQ412057.1 Cassava associated cicular DNA virus isolate G5, complete genome. [29-AUG-2014]	69.7	640	60.94	2220	1	17.83783784	Cassava associated cicular DNA virus:species
Botanical Garden of Copenhagen 61s v2	DQ365814|DQ365814.1 Avian leukosis virus strain MQNCSU, complete genome. [29-AUG-2014]	80.155	701	95.475	7704	2	17.13395639	Avian leukosis virus:species
Botanical Garden of Copenhagen 61s v2	M73260|M73260.1 Mastadenovirus h5 gene, complete genome. [29-AUG-2014]	100	140	97.48333333	35935	3	1.135383331	Human adenovirus 5:no rank
Botanical Garden of Copenhagen 61s v2	JF713713|JF713713.1 Porcine astrovirus 4 strain 35/USA, complete genome. [29-AUG-2014]	64.65	583	90.03	6639	2	15.09263443	Porcine astrovirus 4:species
Botanical Garden of Copenhagen 61s v2	JF755418|JF755418.1 Peromyscus papillomavirus type 1 strain M-14 PmPV1, complete genome. [29-AUG-2	70.08	608.5	90.5	7704	2	13.55140187	Peromyscus papillomavirus type 1:species
Botanical Garden of Copenhagen 61s v2	DQ335246|DQ335246.2 Adeno-associated virus-Go.1, complete genome. [29-AUG-2014]	61.34	9099	24.7	4636	1	48.27437446	Adeno-associated virus-Go.1:no rank
Hong kong 71-73 pool	JQ898342|JQ898342.1 Kobuvirus sewage Kathmandu isolate KoV-SewKTM, complete genome. [29-AUG-201	94.69769231	245.0769231	98.72307692	6939	13	44.74708171	Kobuvirus sewage Kathmandu:no rank
Hong kong 71-73 pool	AF332868|AF332868.1 Tomato mosaic virus isolate Queensland, complete genome. [29-AUG-2014]	99.41	443.5	99.53	6383	2	13.86495378	Tomato mosaic virus:species
Hong kong 71-73 pool	AY150217|AY150217.1 Ambystoma tigrinum stebbensi virus, complete genome. [29-AUG-2014]	75	169	99.41	106332	1	0.157995712	Ambystoma tigrinum stebbensi virus:no rank
Hong kong 71-73 pool	AJ417701|AJ417701.1 Tomato mosaic virus camellia strain, complete genome [29-AUG-2014]	100	138	100	6383	1	2.161992793	Tomato mosaic virus:species
Hong kong 71-73 pool	X02396|X02396.1 Black beetle virus (BBV) RNA1 and RNA3 for protein A and B [29-AUG-2014]	100	119.5	97.92	3106	2	7.533805538	Black beetle virus:species
Hong kong 71-73 pool	EF101928|EF101928.1 Acanthocystis turfacea Chlorella virus 1, complete genome. [29-AUG-2014]	75.58	122.6666667	97.68	288047	3	0.124979604	Acanthocystis turfacea Chlorella virus 1:species
Hong kong 71-73 pool	AJ243571|AJ243571.1 Tomato mosaic virus (K1 strain) complete RNA genome [29-AUG-2014]	99.61	768	99.61	6383	1	11.98496005	Tomato mosaic virus:species
Hong kong 71-73 pool	AB078435|AB078435.1 Tobacco mild green mosaic virus complete genome, strain:Japanese. [29-AUG-2014	96.31666667	183.3333333	98.66333333	6356	3	8.543108874	Tobacco mild green mosaic virus:species
Hong kong 71-73 pool	DQ355023|DQ355023.1 Bell pepper mottle virus, complete genome. [29-AUG-2014]	90.92333333	180.6666667	99.05666667	6375	3	8.423529412	Bell pepper mottle virus:species
Hong kong 71-73 pool	KJ938717|KJ938717.1 Caribou feces-associated gemycircularvirus, complete genome. [14-SEP-2014]	98.75	125	97.2	2232	2	10.88709677	Caribou feces-associated gemycircularvirus:species
Hong kong 71-73 pool	FR878069|FR878069.1 Tobacco mosaic virus strain Ohio V, complete genome, genomic RNA [29-AUG-2014	94.74	115	99.13	6392	1	1.783479349	Tobacco mosaic virus strain Ohio V:no rank
Hong kong 71-73 pool	V01197|V01197.1 Rous sarcoma virus genome, strain Prague C (Pr-C). [29-AUG-2014]	87.88	268	98.99	9312	2	5.702319588	Rous sarcoma virus:species
Hong kong 71-73 pool	JF755427|JF755427.1 Mouse kobuvirus M-5/USA/2010, complete genome. [29-AUG-2014]	85.165	234.5	98.01	8198	4	11.60039034	Mouse kobuvirus M-5/USA/2010:species
Hong kong 71-73 pool	KF371637|KF371637.1 Faecal-associated gemycircularvirus 5, complete genome. [02-OCT-2014]	82.93	125	98.4	2187	1	5.624142661	Faecal-associated gemycircularvirus 5:species
Hong kong 71-73 pool	AB090161|AB090161.1 Theiler's-like virus of rats NGS910 genomic RNA, complete genome. [29-AUG-2014]	89.47	118	96.61	8021	1	1.421269168	Theiler's-like virus of rats:no rank
Hong kong 71-73 pool	M37980|M37980.1 Avian leukosis virus - RSA, complete genome. [29-AUG-2014]	98.23	201	98.98	7286	3	8.193796322	Avian leukosis virus - RSA:no rank
Hong kong 71-73 pool	M20301|M20301.1 Theiler murine encephalomyelitis virus, complete genome. [29-AUG-2014]	84	151	99.34	8093	1	1.853453602	Theiler's encephalomyelitis virus:no rank
Hong kong 71-73 pool	JX185430|JX185430.1 Dragonfly-associated circular virus 1 isolate FL1-2X-2010, complete genome. [29-AU	78.72	141	100	2225	1	6.337078652	Dragonfly-associated circular virus 1:species
Hong kong 71-73 pool	AB089381|AB089381.1 Paprika mild mottle virus complete genome, strain:Japanese. [29-AUG-2014]	100	130.5	100	6524	2	4.000613121	Paprika mild mottle virus:species
Hong kong 71-73 pool	JQ941880|JQ941880.1 Bovine hungarovirus 1 strain BHUV1/2008/HUN, complete genome. [29-AUG-2014]	100	118	99.15	7583	1	1.542924964	Bovine hungarovirus 1:no rank
Hong kong 71-73 pool	KF214645|KF214645.1 Parvo-like hybrid virus UC4, complete genome. [29-AUG-2014]	97.58	547	94.91	3638	2	22.70478285	Parvo-like hybrid virus UC4:no rank
Hong kong 71-73 pool	KC771281|KC771281.1 Human cyclovirus VS5700009, complete genome. [29-AUG-2014]	86.54	160	97.5	1832	1	8.515283843	Human cyclovirus VS5700009:species
Hong kong 71-73 pool	HE818438|HE818438.1 Tobacco mosaic virus isolate Pingtan-1, complete genome [29-AUG-2014]	98.54916667	348.0833333	98.34166667	6395	12	62.6426896	Tobacco mosaic virus:species
Hong kong 71-73 pool	AY859497|AY859497.1 Pepper mild mottle virus isolate PMMoV-CN, complete genome. [29-AUG-2014]	99.174	138.6	99.098	6356	5	10.80868471	Pepper mild mottle virus:species
Hong kong 71-73 pool	U20824|U20824.1 Equid herpesvirus 2, complete genome. [29-AUG-2014]	68.29	153	80.39	184427	1	0.066693055	Equid herpesvirus 2:species
Hong kong 71-73 pool	M34077|M34077.1 Tobacco mild green mosaic virus, complete genome. [29-AUG-2014]	93.48	160.5	98.935	6355	2	5.00393391	Tobacco mild green mosaic virus:species
Hong kong 71-73 pool	DQ890022|DQ890022.1 Paramecium bursaria Chlorella virus FR483, complete genome. [29-AUG-2014]	100	113	98.23	321240	1	0.034553605	Paramecium bursaria Chlorella virus FR483:no rank
Hong kong 71-73 pool	HF920633|HF920633.1 Invertebrate iridovirus 22 complete genome [29-AUG-2014]	76.92	133	87.97	197693	1	0.059182672	Invertebrate iridovirus 22:species
Hong kong 71-73 pool	D12505|D12505.1 Cucumber green mottle mosaic virus RNA, complete genome. [29-AUG-2014]	99.38846154	161.1538462	98.67615385	6424	13	31.80261519	Cucumber green mottle mosaic virus:species
Kuala Lumpur 68s v2	JQ898342|JQ898342.1 Kobuvirus sewage Kathmandu isolate KoV-SewKTM, complete genome. [29-AUG-201	98.99	114.3333333	97.77333333	6939	3	4.842196282	Kobuvirus sewage Kathmandu:no rank
Kuala Lumpur 68s v2	AF332868|AF332868.1 Tomato mosaic virus isolate Queensland, complete genome. [29-AUG-2014]	96.64	358	99.72	6383	1	5.592981357	Tomato mosaic virus:species
Kuala Lumpur 68s v2	KF371634|KF371634.1 Faecal-associated gemycircularvirus 8, complete genome. [02-OCT-2014]	62.47	2633	45.69	2174	1	55.61177553	Faecal-associated gemycircularvirus 8:species
Kuala Lumpur 68s v2	AJ417701|AJ417701.1 Tomato mosaic virus camellia strain, complete genome [29-AUG-2014]	100	476	99.58	6383	1	7.425975247	Tomato mosaic virus:species
Kuala Lumpur 68s v2	JN648103|JN648103.1 Canine bocavirus 1 isolate Con-161, complete genome. [29-AUG-2014]	46.45	2879	64.61	5413	1	34.97136523	Canine bocavirus 1:no rank
Kuala Lumpur 68s v2	KJ547631|KJ547631.1 Sewage-associated circular DNA virus-7 isolate SaCV-7 NZ-BS3976-2012, complete	79.59	152	96.71	2311	1	6.360882735	Sewage-associated circular DNA virus-7:species
Kuala Lumpur 68s v2	U66304|U66304.1 Chicken anemia virus cloned isolate 10, complete genome. [29-AUG-2014]	98.63	1534	99.74	2319	1	65.9767141	Chicken anemia virus:species
Kuala Lumpur 68s v2	EF101928|EF101928.1 Acanthocystis turfacea Chlorella virus 1, complete genome. [29-AUG-2014]	76.18	144.75	97.7825	288047	4	0.191635393	Acanthocystis turfacea Chlorella virus 1:species
Kuala Lumpur 68s v2	AB078435|AB078435.1 Tobacco mild green mosaic virus complete genome, strain:Japanese. [29-AUG-2014	99.6925	227.25	98.085	6356	4	14.01825047	Tobacco mild green mosaic virus:species
Kuala Lumpur 68s v2	DQ355023|DQ355023.1 Bell pepper mottle virus, complete genome. [29-AUG-2014]	90.16857143	192.5714286	99.16428571	6375	7	21.50588235	Bell pepper mottle virus:species
Kuala Lumpur 68s v2	KJ938717|KJ938717.1 Caribou feces-associated gemycircularvirus, complete genome. [14-SEP-2014]	73.155	1523.5	46.96	2232	2	55.82437276	Caribou feces-associated gemycircularvirus:species
Kuala Lumpur 68s v2	AF063497|AF063497.1 Adeno-associated virus 1, complete genome. [29-AUG-2014]	66.87	496	98.59	4718	1	10.36456125	Adeno-associated virus - 1:no rank
Kuala Lumpur 68s v2	AB040749|AB040749.1 Aichi virus genomic RNA, complete genome. [29-AUG-2014]	92.45	161	98.76	8280	1	1.920289855	Aichivirus A:species
Kuala Lumpur 68s v2	JN704610|JN704610.1 Meles meles fecal virus isolate VS4700006, complete genome. [29-AUG-2014]	74.84	5228	11.62	2198	2	55.41401274	Meles meles fecal virus:species
Kuala Lumpur 68s v2	JF755427|JF755427.1 Mouse kobuvirus M-5/USA/2010, complete genome. [29-AUG-2014]	92.2	269	28.995	8198	2	1.902903147	Mouse kobuvirus M-5/USA/2010:species
Kuala Lumpur 68s v2	JQ692586|JQ692586.1 Feline bocavirus strain HK797U, complete genome. [29-AUG-2014]	82.19	222	98.65	5179	1	4.228615563	Feline bocavirus:no rank
Kuala Lumpur 68s v2	M81888|M81888.1 Parvovirus Lull DNA sequence. [29-AUG-2014]	93.75	128	98.405	5135	2	4.907497566	LuIII virus:no rank
Kuala Lumpur 68s v2	AF395114|AF395114.1 Chicken anemia virus strain BD-3, complete genome. [29-AUG-2014]	100	399	96.99	2298	1	16.84073107	Chicken anemia virus:species

Kuala Lumpur 68s v2	AB090161|AB090161.1 Theiler's-like virus of rats NGS910 genomic RNA, complete genome. [29-AUG-2014]	84.04	7625	88.37	8021	1	84.07929186	Theiler's-like virus of rats:no rank
Kuala Lumpur 68s v2	EU376394|EU376394.1 Human TMEV-like cardiovirus, complete genome. [29-AUG-2014]	52.46	8485	26.34	7961	1	27.77289285	Human TMEV-like cardiovirus:no rank
Kuala Lumpur 68s v2	AF174533|AF174533.1 Nodamura virus RNA1 segment, complete sequence. [29-AUG-2014]	74.58	193	91.71	3204	1	5.524344569	Nodamura virus:species
Kuala Lumpur 68s v2	M37980|M37980.1 Avian leukosis virus - RSA, complete genome. [29-AUG-2014]	98.73	592	99.23666667	7286	3	24.29316497	Avian leukosis virus - RSA:no rank
Kuala Lumpur 68s v2	AF033807|AF033807.1 Mouse mammary tumor virus complete proviral genome. [29-AUG-2014]	85.71	108	97.22	8805	1	1.192504259	Mouse mammary tumor virus:species
Kuala Lumpur 68s v2	KF170373|KF170373.1 Parvo-like hybrid virus UC1, complete genome. [29-AUG-2014]	100	794	99.75	3638	1	21.77020341	Parvo-like hybrid virus UC1:no rank
Kuala Lumpur 68s v2	X02449|X02449.1 Hamster polyomavirus (HapV) genome [29-AUG-2014]	78.39	2110	51.8825	5366	4	67.08907939	Hamster polyomavirus:species
Kuala Lumpur 68s v2	X01457|X01457.1 Parvovirus h-1, complete genome [29-AUG-2014]	81.76	430	99.77	5176	1	8.520092736	H-1 parvovirus:no rank
Kuala Lumpur 68s v2	HQ425636|HQ425636.1 Avian leukemia virus isolate SCDY1, complete genome. [29-AUG-2014]	97.92	144	100	7489	1	1.922820136	Avian leukemia virus:no rank
Kuala Lumpur 68s v2	GQ404855|GQ404855.1 Cyclovirus NG14, complete genome. [29-AUG-2014]	68.41	1970	51.02	1795	1	57.49303621	Cyclovirus NG14:species
Kuala Lumpur 68s v2	AB089381|AB089381.1 Paprika mild mottle virus complete genome, strain:Japanese. [29-AUG-2014]	98.980625	364.5	99.1975	6524	16	87.06315144	Paprika mild mottle virus:species
Kuala Lumpur 68s v2	X00956|X00956.1 Black beetle virus (BBV) RNA2 sequence for coat protein precursor [29-AUG-2014]	93.75333333	276	98.96	1399	3	58.3273767	Black beetle virus:species
Kuala Lumpur 68s v2	KF738874|KF738874.1 Dragonfly larvae associated circular virus-2 isolate DflaCV-2 NZ-PG8-LS, complete ge	72.285	887.5	52.09	2398	2	32.90241868	Dragonfly larvae associated circular virus-2:species
Kuala Lumpur 68s v2	HE818438|HE818438.1 Tobacco mosaic virus isolate Pingtan-1, complete genome [29-AUG-2014]	96.8325	163.75	98.675	6395	4	10.13291634	Tobacco mosaic virus:species
Kuala Lumpur 68s v2	AY859497|AY859497.1 Pepper mild mottle virus isolate PMMoV-CN, complete genome. [29-AUG-2014]	99.70166667	776.3333333	99.41666667	6356	6	68.25047199	Pepper mild mottle virus:species
Kuala Lumpur 68s v2	HM029238|HM029238.1 Avian nephritis virus 1 from China, complete genome. [29-AUG-2014]	80	152	98.68	6941	1	1.98818614	Avian nephritis virus 1:no rank
Kuala Lumpur 68s v2	KJ547633|KJ547633.1 Sewage-associated circular DNA virus-9 isolate SaCV-9 NZ-BS3681-2012, complete	65.045	410	99.035	2423	2	29.71522905	Sewage-associated circular DNA virus-9:species
Kuala Lumpur 68s v2	S47266|S47266.1 orf1...orf4 [Junonia coenia densovirus JcDNV, Genomic Complete, 4 genes, 5908 nt]. [29	85.89666667	1414	82.44	5908	3	54.73933649	Junonia coenia densovirus:no rank
Kuala Lumpur 68s v2	JX827169|JX827169.1 Rat parvovirus NTU1, complete genome. [29-AUG-2014]	89.45	159	93.15	4759	3	9.077537298	Rat parvovirus NTU1:species
Kuala Lumpur 68s v2	KJ547625|KJ547625.1 Sewage-associated circular DNA virus-14 isolate SaCV-14 NZ-BS4064-2012, complet	97.5	121	99.17	2335	1	5.139186296	Sewage-associated circular DNA virus-14:species
Kuala Lumpur 68s v2	KF413620|KF413620.1 Hypericum japonicum associated circular DNA virus isolate VNHJ1W, complete genor	71.795	226.5	99.125	2200	2	19.90909091	Hypericum japonicum associated circular DNA virus:species
Kuala Lumpur 68s v2	AY461507|AY461507.1 Mythimna loreyi densovirus, complete genome. [29-AUG-2014]	89.6175	610.75	89.015	6034	4	37.68644349	Mythimna loreyi densovirus:no rank
Kuala Lumpur 68s v2	X02396|X02396.1 Black beetle virus (BBV) RNA1 and RNA3 for protein A and B [29-AUG-2014]	95.155	368.5	98.95	3106	6	70.7984546	Black beetle virus:species
Kuala Lumpur 68s v2	KJ547630|KJ547630.1 Sewage-associated circular DNA virus-6 isolate SaCV-6 NZ-BS4017-2012, complete	66.32	330	86.36	2540	1	11.22047244	Sewage-associated circular DNA virus-6:species
Kuala Lumpur 68s v2	KF738873|KF738873.1 Dragonfly larvae associated circular virus-1 isolate DflaCV-1 NZ-PG11-LD, complete (	59.9	1702	35.43	2668	1	22.03898051	Dragonfly larvae associated circular virus-1:species
Kuala Lumpur 68s v2	JQ085285|JQ085285.1 Meles meles circovirus-like virus, complete genome. [29-AUG-2014]	51.88	2353	60.05	2218	1	63.30027051	Meles meles circovirus-like virus:species
Kuala Lumpur 68s v2	JQ303225|JQ303225.1 Rhinolophus ferrumequinum retrovirus isolate RfRV, complete genome. [02-JUL-201	73.58	163	97.55	8389	1	1.895339135	Rhinolophus ferrumequinum retrovirus:no rank
Kuala Lumpur 68s v2	JX645345|JX645345.1 Rat minute virus isolate NTU2, complete genome. [29-AUG-2014]	92.67666667	649.8333333	99.23	4780	6	78.76569038	Rat minute virus:species
Kuala Lumpur 68s v2	DQ100461|DQ100461.1 Beilong virus, complete genome. [29-AUG-2014]	96.44833333	141.1666667	98.13666667	19212	6	4.325421611	Beilong virus:species
Kuala Lumpur 68s v2	FJ445512|FJ445512.1 Mouse parvovirus 4b, complete genome. [29-AUG-2014]	87.18	237	98.73	4794	1	4.755944931	Mouse parvovirus 4b:no rank
Kuala Lumpur 68s v2	FJ959081|FJ959081.1 Circovirus-like genome RW-E, complete genome. [29-AUG-2014]	59.16	1257	62.53	2782	1	28.14521927	Circovirus-like genome RW-E:species
Kuala Lumpur 68s v2	JQ308210|JQ308210.1 Gyrovirus GyV3, complete genome. [29-AUG-2014]	68.41	129.5	98.45	2359	2	13.48028826	Gyrovirus GyV3:species
Kuala Lumpur 68s v2	GU244497|GU244497.1 Cafeteria roenbergensis virus BV-PW1, complete genome. [29-AUG-2014]	83.33	148	97.3	617453	1	0.023321613	Cafeteria roenbergensis virus BV-PW1:no rank
Kuala Lumpur 68s v2	JX310702|JX310702.1 Gyrovirus 4 strain D137, complete genome. [29-AUG-2014]	95.865	170.5	99.315	2034	2	16.66666667	Gyrovirus 4:species
Kuala Lumpur 68s v2	U60315|U60315.1 Molluscum contagiosum virus subtype 1, complete genome. [29-AUG-2014]	79.25	161	98.76	190289	1	0.083557116	Molluscum contagiosum virus subtype 1:no rank
Kuala Lumpur 68s v2	HM004429|HM004429.1 Micromonas sp. RCC1109 virus MpV1, complete genome. [29-AUG-2014]	71.88	196	97.96	184095	1	0.104293979	Micromonas sp. RCC1109 virus MpV1:species
Kuala Lumpur 68s v2	KF938901|KF938901.1 UNVERIFIED: Anopheles minimus irodovirus isolate AMIV, complete genome. [29-AUG	84	152	98.68	163023	1	0.092011557	Anopheles minimus irodovirus:species
Kuala Lumpur 68s v2	AF303741|AF303741.1 Chilo iridescent virus complete genome. [29-AUG-2014]	85	123	97.56	212482	1	0.056475372	Invertebrate iridescent virus 6:species
Kuala Lumpur 68s v2	FR878069|FR878069.1 Tobacco mosaic virus strain Ohio V, complete genome, genomic RNA [29-AUG-2014	98.08	156	100	6392	1	2.440550688	Tobacco mosaic virus strain Ohio V:no rank
Kuala Lumpur 68s v2	KC580640|KC580640.1 Canine bocavirus 3 isolate UCD, complete genome. [29-AUG-2014]	49.89	1739	76.08	5278	1	25.35051156	Canine bocavirus 3:species
Kuala Lumpur 68s v2	KF371633|KF371633.1 Faecal-associated gemycircularvirus 9, complete genome. [02-OCT-2014]	61.24	774	50	2130	1	16.76056338	Faecal-associated gemycircularvirus 9:species
Kuala Lumpur 68s v2	V01197|V01197.1 Rous sarcoma virus genome, strain Prague C (Pr-C). [29-AUG-2014]	95.7	237.6666667	97.57666667	9312	3	7.506443299	Rous sarcoma virus:species
Kuala Lumpur 68s v2	DQ196318|DQ196318.1 Mouse parvovirus 3, complete genome. [29-AUG-2014]	87.47	1585	79.87	4787	1	26.50929601	Mouse parvovirus 3:species
Kuala Lumpur 68s v2	KF371637|KF371637.1 Faecal-associated gemycircularvirus 5, complete genome. [02-OCT-2014]	51	3105	19.13	2187	1	27.16049383	Faecal-associated gemycircularvirus 5:species
Kuala Lumpur 68s v2	KC991097|KC991097.1 Solenopsis invicta densovirus isolate SiDNV-Arg, complete genome. [29-AUG-2014]	51.19	1059	83.29	5280	1	16.13636364	Solenopsis invicta densovirus:species
Kuala Lumpur 68s v2	JQ815363|JQ815363.1 Cyprinid herpesvirus 1 strain NG-J1, complete genome. [29-AUG-2014]	69.23	511	30.53	291144	1	0.042247135	Cyprinid herpesvirus 1:species
Kuala Lumpur 68s v2	GU991616|GU991616.1 Providence virus isolate vFLM1, complete genome. [29-AUG-2014]	100	134.3333333	96.53	6155	3	6.336311942	Providence virus:species
Kuala Lumpur 68s v2	JQ340310|JQ340310.1 Astrovirus wild boar/WBAstV-1/2011/HUN, complete genome. [29-AUG-2014]	51.3	476	96.43	6707	1	5.77009095	Astrovirus wild boar/WBAstV-1/2011/HUN:species
Kuala Lumpur 68s v2	DQ333351|DQ333351.1 Choristoneura occidentalis granulovirus, complete genome. [29-AUG-2014]	92.31	364	21.43	104710	1	0.074491453	Choristoneura occidentalis granulovirus:species
Kuala Lumpur 68s v2	JX185430|JX185430.1 Dragonfly-associated circular virus 1 isolate FL1-2X-2010, complete genome. [29-AU	68.73	2249	21.745	2225	2	44.08988764	Dragonfly-associated circular virus 1:species
Kuala Lumpur 68s v2	AB261167|AB261167.1 Cucumber mottle virus genomic RNA, complete genome. [29-AUG-2014]	91.38	137	97.455	6485	2	4.117193524	Cucumber mottle virus:species
Kuala Lumpur 68s v2	KF214638|KF214638.1 Parvo-like hybrid virus UC11, complete genome. [29-AUG-2014]	99.81	699.5	99.725	3637	2	38.35578774	Parvo-like hybrid virus UC11:no rank
Kuala Lumpur 68s v2	JX544744|JX544744.1 Murine astrovirus strain STL 2, complete genome. [29-AUG-2014]	63.19666667	681.6666667	94.45666667	6827	3	27.94785411	Murine astrovirus:species
Kuala Lumpur 68s v2	GQ365709|GQ365709.1 Sclerotinia sclerotiorum hypovirulence associated DNA virus 1, complete genome. [2	82.69	157	99.36	2166	1	7.202216066	Sclerotinia sclerotiorum hypovirulence associated DNA virus 1:species
Kuala Lumpur 68s v2	JX185428|JX185428.1 Dragonfly-associated circular virus 3 isolate TO-DFS3B2-2010, complete genome. [2	56.58	2729	16.6	2208	1	19.70108696	Dragonfly-associated circular virus 3:species
Kuala Lumpur 68s v2	M34077|M34077.1 Tobacco mild green mosaic virus, complete genome. [29-AUG-2014]	100	171	100	6355	1	2.69079465	Tobacco mild green mosaic virus:species
Kuala Lumpur 68s v2	JF411744|JF411744.1 Paramecium bursaria Chlorella virus 1 (PBCV-1), complete genome. [29-AUG-2014]	80.43	142	97.18	330611	1	0.041740898	Paramecium bursaria Chlorella virus 1:species
Kuala Lumpur 68s v2	AB628188|AB628188.1 Rehmannia mosaic virus genomic RNA, complete genome, isolate: Japanese. [29-AU	100	109	99.08	6395	1	1.68881939	Rehmannia mosaic virus:species
Kuala Lumpur 68s v2	DQ890022|DQ890022.1 Paramecium bursaria Chlorella virus FR483, complete genome. [29-AUG-2014]	91.8575	128.5625	99.048125	321240	16	0.634105342	Paramecium bursaria Chlorella virus FR483:no rank
Kuala Lumpur 68s v2	KJ547622|KJ547622.1 Sewage-associated circular DNA virus-11 isolate SaCV-11 NZ-BS3997-2012, comple	89.34	113	98.22	2336	2	9.503424658	Sewage-associated circular DNA virus-11:species
Kuala Lumpur 68s v2	V01170|V01170.1 Y73 sarcoma virus complete genome, genomic RNA [29-AUG-2014]	92.05	337	78.34	3718	1	7.100591716	Y73 sarcoma virus:species
Kuala Lumpur 68s v2	D12505|D12505.1 Cucumber green mottle mosaic virus RNA, complete genome. [29-AUG-2014]	99.36	1519	99.7475	6424	4	94.38044832	Cucumber green mottle mosaic virus:species
Kuala Lumpur 68s v2	DQ365814|DQ365814.1 Avian leukosis virus strain MQNCSU, complete genome. [29-AUG-2014]	87.42333333	516.6666667	98.90666667	7704	3	19.58722741	Avian leukosis virus:species
Kuala Lumpur 68s v2	JF713713|JF713713.1 Porcine astrovirus 4 strain 35/USA, complete genome. [29-AUG-2014]	76	151	99.34	6639	1	2.259376412	Porcine astrovirus 4:species
Kuala Lumpur 68s v2	FR823283|FR823283.1 Human gyrovirus type 1 complete genome, isolate 915 F 06 007 FD [29-AUG-2014]	100	140	98.57	2315	1	5.96112311	Human gyrovirus type 1:species
Kuala Lumpur 68s v2	DQ335246|DQ335246.2 Adeno-associated virus-Go.1, complete genome. [29-AUG-2014]	61.34	3031	74.13	4636	1	48.27437446	Adeno-associated virus-Go.1:no rank
Kuala Lumpur 69s	JQ898342|JQ898342.1 Kobuvirus sewage Kathmandu isolate KoV-SewKTM, complete genome. [29-AUG-201	93.09285714	886.5714286	99.67714286	6939	7	84.34932987	Kobuvirus sewage Kathmandu:no rank
Kuala Lumpur 69s	AF332868|AF332868.1 Tomato mosaic virus isolate Queensland, complete genome. [29-AUG-2014]	100	133	99.25	6383	1	2.067993107	Tomato mosaic virus:species
Kuala Lumpur 69s	KF831027|KF831027.1 Feline kobuvirus strain FK-13, complete genome. [29-AUG-2014]	54.3	977	63.56	8219	1	7.774668451	Feline kobuvirus:no rank
Kuala Lumpur 69s	KF738873|KF738873.1 Dragonfly larvae associated circular virus-1 isolate DflaCV-1 NZ-PG11-LD, complete (	59.69	969	78.64	2668	1	29.01049475	Dragonfly larvae associated circular virus-1:species
Kuala Lumpur 69s	JQ085285|JQ085285.1 Meles meles circovirus-like virus, complete genome. [29-AUG-2014]	70.91	163	99.39	2218	1	7.439134355	Meles meles circovirus-like virus:species
Kuala Lumpur 69s	JX645345|JX645345.1 Rat minute virus isolate NTU2, complete genome. [29-AUG-2014]	83.93	170	98.82	4780	1	3.514644351	Rat minute virus:species
Kuala Lumpur 69s	KF500001|KF500001.1 Formica exsecta virus 1 isolate Fex1, complete genome. [29-AUG-2014]	78	150	100	9554	1	1.570023027	Formica exsecta virus 1:species
Kuala Lumpur 69s	KJ577817|KJ577817.1 Porcine stool-associated circular virus 8 isolate GP2, complete genome. [02-SEP-201	49.69	2228	39.32	2477	1	38.75656036	Porcine stool-associated circular virus 8:species
Kuala Lumpur 69s	AB078435|AB078435.1 Tobacco mild green mosaic virus complete genome, strain:Japanese. [29-AUG-2014	84.21	115	99.13	6356	1	1.793580868	Tobacco mild green mosaic virus:species
Kuala Lumpur 69s	KJ938717|KJ938717.1 Caribou feces-associated gemycircularvirus, complete genome. [14-SEP-2014]	78.6	388.2	98.31	2232	5	83.2437276	Caribou feces-associated gemycircularvirus:species
Kuala Lumpur 69s	GU244497|GU244497.1 Cafeteria roenbergensis virus BV-PW1, complete genome. [29-AUG-2014]	55.2	849	41.34	617453	1	0.058304033	Cafeteria roenbergensis virus BV-PW1:no rank
Kuala Lumpur 69s	AF033810|AF033810.1 Fujinami sarcoma virus, complete genome. [29-AUG-2014]	97.37	115	99.13	4788	1	2.380952381	Fujinami sarcoma virus:species
Kuala Lumpur 69s	AY388617|AY388617.1 Bovine adeno-associated virus, complete genome. [29-AUG-2014]	84.04	282	100	4693	1	6.008949499	Bovine adeno-associated virus:no rank
Kuala Lumpur 69s	AF303741|AF303741.1 Chilo iridescent virus complete genome. [29-AUG-2014]	49.58	359	97.77	212482	1	0.149659736	Invertebrate iridescent virus 6:species
Kuala Lumpur 69s	FR878069|FR878069.1 Tobacco mosaic virus strain Ohio V, complete genome, genomic RNA [29-AUG-2014	97.118	190	98.446	6392	5	14.6902378	Tobacco mosaic virus strain Ohio V:no rank
Kuala Lumpur 69s	JF938082|JF938082.1 Bat circovirus ZS/China/2011 isolate YN-BtCV-5, complete genome. [29-AUG-2014]	64.29	485	99.59	1818	1	27.72277228	Bat circovirus ZS/China/2011:species
Kuala Lumpur 69s	KC580640|KC580640.1 Canine bocavirus 3 isolate UCD, complete genome. [29-AUG-2014]	51.02	5002	34.07	5278	1	32.56915498	Canine bocavirus 3:species
Kuala Lumpur 69s	JN704610|JN704610.1 Meles meles fecal virus isolate VS4700006, complete genome. [29-AUG-2014]	61.63	1193.5	60.705	2198	2	68.24385805	Meles meles fecal virus:species
Kuala Lumpur 69s	V01197|V01197.1 Rous sarcoma virus genome, strain Prague C (Pr-C). [29-AUG-2014]	92.1225	327.75	94.3625	9312	8	24.25902062	Rous sarcoma virus:species
Kuala Lumpur 69s	JF755427|JF755427.1 Mouse kobuvirus M-5/USA/2010, complete genome. [29-AUG-2014]	91.18	113	90.27	8198	1	1.244205904	Mouse kobuvirus M-5/USA/2010:species
Kuala Lumpur 69s	JQ692586|JQ692586.1 Feline bocavirus strain HK797U, complete genome. [29-AUG-2014]	81.93	249	100	5179	1	4.807877969	Feline bocavirus:no rank
Kuala Lumpur 69s	DQ196318|DQ196318.1 Mouse parvovirus 3, complete genome. [29-AUG-2014]	84.15	249	98.8	4787	1	5.138917903	Mouse parvovirus 3:species
Kuala Lumpur 69s	KF371637|KF371637.1 Faecal-associated gemycircularvirus 5, complete genome. [02-OCT-2014]	48.47	2678	24.65	2187	1	30.58984911	Faecal-associated gemycircularvirus 5:species
Kuala Lumpur 69s	KC991097|KC991097.1 Solenopsis invicta densovirus isolate SiDNV-Arg, complete genome. [29-AUG-2014]	83.08	198	98.48	5280	1	3.693181818	Solenopsis invicta densovirus:species
Kuala Lumpur 69s	AB090161|AB090161.1 Theiler's-like virus of rats NGS910 genomic RNA, complete genome. [29-AUG-2014]	93.48	2088.5	99.815	8021	2	52.01346466	Theiler's-like virus of rats:no rank
Kuala Lumpur 69s	AF150629|AF150629.1 Acute bee paralysis virus, complete genome. [29-AUG-2014]	84.85	201	98.51	9491	1	2.086186914	Acute bee paralysis virus:species
Kuala Lumpur 69s	JQ037754|JQ037754.1 Artibeus jamaicensis parvovirus 1, complete genome. [29-AUG-2014]	47.5	1351	35.53	4595	1	10.31556039	Artibeus jamaicensis parvovirus 1:species
Kuala Lumpur 69s	EU376394|EU376394.1 Human TMEV-like cardiovirus, complete genome. [29-AUG-2014]	71.01	420	98.57	7961	1	5.200351715	Human TMEV-like cardiovirus:no rank
Kuala Lumpur 69s	M37980|M37980.1 Avian leukosis virus - RSA, complete genome. [29-AUG-2014]	98.55	209	99.04	7286	1	2.841065056	Avian leukosis virus - RSA:no rank
Kuala Lumpur 69s	M20301|M20301.1 Theiler murine encephalomyelitis virus, complete genome. [29-AUG-2014]	92.11	381	29.92	8093	1	1.408624737	Theiler's encephalomyelitis virus:no rank
Kuala Lumpur 69s	JQ340310|JQ340310.1 Astrovirus wild boar/WBAstV-1/2011/HUN, complete genome. [29-AUG-2014]	53.1	350	96.86	6707	1	4.115103623	Astrovirus wild boar/WBAstV-1/2011/HUN:species
Kuala Lumpur 69s	HQ425636|HQ425636.1 Avian leukemia virus isolate SCDY1, complete genome. [29-AUG-2014]	97.92	147	97.96	7489	1	1.922820136	Avian leukemia virus:no rank
Kuala Lumpur 69s	GQ404855|GQ404855.1 Cyclovirus NG14, complete genome. [29-AUG-2014]	75	158	98.73	1795	1	8.690807799	Cyclovirus NG14:species
Kuala Lumpur 69s	JX185430|JX185430.1 Dragonfly-associated circular virus 1 isolate FL1-2X-2010, complete genome. [29-AU	60.45	4040	9.95	2225	1	17.66292135	Dragonfly-associated circular virus 1:species
Kuala Lumpur 69s	AB261167|AB261167.1 Cucumber mottle virus genomic RNA, complete genome. [29-AUG-2014]	95.99625	139.25	98.205	6485	8	16.88511951	Cucumber mottle virus:species
Kuala Lumpur 69s	AB010145|AB010145.1 Aichi virus genomic RNA, complete genome. [29-AUG-2014]	74.22	880	42.27	8251	1	4.653981336	Aichivirus A:species
Kuala Lumpur 69s	KJ158169|KJ158169.1 Rosavirus 2 strain GA7403, complete genome. [29-AUG-2014]	70.69	176	98.86	8931	1	1.948270071	Rosavirus A2:subspecies
Kuala Lumpur 69s	KJ547632|KJ547632.1 Sewage-associated circular DNA virus-8 isolate SaCV-8 NZ-BS4075-2012, complete	82.86	107	98.13	2577	1	4.074505239	Sewage-associated circular DNA virus-8:species
Kuala Lumpur 69s	AB089381|AB089381.1 Paprika mild mottle virus complete genome, strain:Japanese. [29-AUG-2014]	99.04466667	322.6	98.958	6524	15	73.2066217	Paprika mild mottle virus:species
Kuala Lumpur 69s	AY037928|AY037928.1 Human endogenous retrovirus K113 complete genome. [29-AUG-2014]	97.22	110	98.18	9472	1	1.140202703	Human endogenous retrovirus K113:no rank
Kuala Lumpur 69s	JQ941880|JQ941880.1 Bovine hungarovirus 1 strain BHUV1/2008/HUN, complete genome. [29-AUG-2014]	72.73	169	97.63	7583	1	2.175919821	Bovine hungarovirus 1:no rank
Kuala Lumpur 69s	KF738874|KF738874.1 Dragonfly larvae associated circular virus-2 isolate DflaCV-2 NZ-PG8-LS, complete ge	65.67	584	34.42	2398	1	8.381984987	Dragonfly larvae associated circular virus-2:species
Kuala Lumpur 69s	JX544744|JX544744.1 Murine astrovirus strain STL 2, complete genome. [29-AUG-2014]	74.96666667	240.3333333	99.33333333	6827	3	10.50241687	Murine astrovirus:species
Kuala Lumpur 69s	KF958461|KF958461.1 Mosavirus A2 strain SZAL6-MoV/2011/HUN, complete genome. [29-AUG-2014]	79.59	147	100	8398	1	1.750416766	Mosavirus A2:no rank
Kuala Lumpur 69s	KC543331|KC543331.1 Acheta domesticus volvovirus isolate IAF, complete genome. [29-AUG-2014]	67.31	163	95.71	2517	1	6.197854589	Acheta domesticus volvovirus:species
Kuala Lumpur 69s	JX185428|JX185428.1 Dragonfly-associated circular virus 3 isolate TO-DFS3B2-2010, complete genome. [2	55.84	230	99.13	2208	1	10.46195652	Dragonfly-associated circular virus 3:species
Kuala Lumpur 69s	HE818438|HE818438.1 Tobacco mosaic virus isolate Pingtan-1, complete genome [29-AUG-2014]	99.38	720.5	93.29833333	6395	6	59.82799062	Tobacco mosaic virus:species
Kuala Lumpur 69s	AY859497|AY859497.1 Pepper mild mottle virus isolate PMMoV-CN, complete genome. [29-AUG-2014]	99.566	127	98.734	6356	5	9.864694777	Pepper mild mottle virus:species
Kuala Lumpur 69s	KJ547620|KJ547620.1 Sewage-associated circular DNA virus-1 isolate SaCV-1 NZ-BS3349-2012, complete	76.6	155	90.97	2777	1	5.077421678	Sewage-associated circular DNA virus-1:species
Kuala Lumpur 69s	M34077|M34077.1 Tobacco mild green mosaic virus, complete genome. [29-AUG-2014]	94.12	103	99.03	6355	1	1.605035405	Tobacco mild green mosaic virus:species
Kuala Lumpur 69s	EU815052|EU815052.1 Rat theilovirus 1, complete genome. [29-AUG-2014]	86.445	1311	99.575	7996	2	32.52876438	Rat theilovirus 1:no rank
Kuala Lumpur 69s	AB628188|AB628188.1 Rehmannia mosaic virus genomic RNA, complete genome, isolate: Japanese. [29-AU	98.19666667	165.6666667	96.44333333	6395	3	7.552775606	Rehmannia mosaic virus:species
Kuala Lumpur 69s	HF920633|HF920633.1 Invertebrate iridovirus 22 complete genome [29-AUG-2014]	86.96	139	99.28	197693	1	0.069805203	Invertebrate iridovirus 22:species
Kuala Lumpur 69s	JX827169|JX827169.1 Rat parvovirus NTU1, complete genome. [29-AUG-2014]	85.665	250.75	98.2925	4759	4	20.991805	Rat parvovirus NTU1:species
Kuala Lumpur 69s	D12505|D12505.1 Cucumber green mottle mosaic virus RNA, complete genome. [29-AUG-2014]	99.295	3345.5	99.705	6424	2	98.95703611	Cucumber green mottle mosaic virus:species
Kuala Lumpur 69s	DQ365814|DQ365814.1 Avian leukosis virus strain MQNCSU, complete genome. [29-AUG-2014]	99.42666667	368.3333333	99.20333333	7704	3	13.83696781	Avian leukosis virus:species
Kuala Lumpur 69s	M73260|M73260.1 Mastadenovirus h5 gene, complete genome. [29-AUG-2014]	100	158	95.5975	35935	4	1.686378183	Human adenovirus 5:no rank
Kuala Lumpur 69s	JF713713|JF713713.1 Porcine astrovirus 4 strain 35/USA, complete genome. [29-AUG-2014]	69.92	369	100	6639	1	5.558065974	Porcine astrovirus 4:species
Kuala Lumpur 69s	DQ335246|DQ335246.2 Adeno-associated virus-Go.1, complete genome. [29-AUG-2014]	59.765	1199	72.785	4636	2	43.80931838	Adeno-associated virus-Go.1:no rank
Kuala Lumpur 70	JQ898342|JQ898342.1 Kobuvirus sewage Kathmandu isolate KoV-SewKTM, complete genome. [29-AUG-201	94.44625	504.25	99.51625	6939	8	58.14958928	Kobuvirus sewage Kathmandu:no rank
Kuala Lumpur 70	AF332868|AF332868.1 Tomato mosaic virus isolate Queensland, complete genome. [29-AUG-2014]	100	150	100	6383	1	2.349992167	Tomato mosaic virus:species
Kuala Lumpur 70	KF831027|KF831027.1 Feline kobuvirus strain FK-13, complete genome. [29-AUG-2014]	91.18	103	99.03	8219	1	1.241026889	Feline kobuvirus:no rank
Kuala Lumpur 70	AY150217|AY150217.1 Ambystoma tigrinum stebbensi virus, complete genome. [29-AUG-2014]	61.4	173	98.84	106332	1	0.146710304	Ambystoma tigrinum stebbensi virus:no rank
Kuala Lumpur 70	AJ417701|AJ417701.1 Tomato mosaic virus camellia strain, complete genome [29-AUG-2014]	99.25	401	99.5	6383	1	6.250979163	Tomato mosaic virus:species
Kuala Lumpur 70	X02396|X02396.1 Black beetle virus (BBV) RNA1 and RNA3 for protein A and B [29-AUG-2014]	100	120	96.26	3106	2	7.437218287	Black beetle virus:species
Kuala Lumpur 70	AJ243571|AJ243571.1 Tomato mosaic virus (K1 strain) complete RNA genome [29-AUG-2014]	98.525	561	99.635	6383	2	17.53094156	Tomato mosaic virus:species
Kuala Lumpur 70	GQ918152|GQ918152.1 Wiseana iridescent virus, complete genome. [29-AUG-2014]	91.67	148	97.3	205791	1	0.069973906	Wiseana iridescent virus:no rank
Kuala Lumpur 70	AB078435|AB078435.1 Tobacco mild green mosaic virus complete genome, strain:Japanese. [29-AUG-2014	98.9	175	99.5	6356	3	8.212712398	Tobacco mild green mosaic virus:species
Kuala Lumpur 70	DQ355023|DQ355023.1 Bell pepper mottle virus, complete genome. [29-AUG-2014]	87.36666667	139	97.86333333	6375	3	6.4	Bell pepper mottle virus:species
Kuala Lumpur 70	FJ959081|FJ959081.1 Circovirus-like genome RW-E, complete genome. [29-AUG-2014]	83.33	142	88.73	2782	1	4.529115744	Circovirus-like genome RW-E:species
Kuala Lumpur 70	X13744|X13744.1 Avian Retrovirus IC10 proviral genome [29-AUG-2014]	97.7	262	99.62	4213	1	6.195110373	Avian retrovirus IC10:species
Kuala Lumpur 70	GU244497|GU244497.1 Cafeteria roenbergensis virus BV-PW1, complete genome. [29-AUG-2014]	86.67	139	97.12	617453	1	0.021864012	Cafeteria roenbergensis virus BV-PW1:no rank
Kuala Lumpur 70	FR878069|FR878069.1 Tobacco mosaic virus strain Ohio V, complete genome, genomic RNA [29-AUG-2014	98.98	136	98.18	6392	2	4.17709637	Tobacco mosaic virus strain Ohio V:no rank
Kuala Lumpur 70	V01197|V01197.1 Rous sarcoma virus genome, strain Prague C (Pr-C). [29-AUG-2014]	90.015	278.75	96.05	9312	4	11.37242268	Rous sarcoma virus:species
Kuala Lumpur 70	JF755427|JF755427.1 Mouse kobuvirus M-5/USA/2010, complete genome. [29-AUG-2014]	88.98857143	208.4285714	83.74714286	8198	7	15.66235667	Mouse kobuvirus M-5/USA/2010:species
Kuala Lumpur 70	KF371637|KF371637.1 Faecal-associated gemycircularvirus 5, complete genome. [02-OCT-2014]	65.31	153	96.08	2187	1	6.721536351	Faecal-associated gemycircularvirus 5:species
Kuala Lumpur 70	KC991097|KC991097.1 Solenopsis invicta densovirus isolate SiDNV-Arg, complete genome. [29-AUG-2014]	81.0725	195	96.38	5280	4	14.43181818	Solenopsis invicta densovirus:species
Kuala Lumpur 70	AB090161|AB090161.1 Theiler's-like virus of rats NGS910 genomic RNA, complete genome. [29-AUG-2014]	92.635	159	99.02	8021	2	3.927191123	Theiler's-like virus of rats:no rank
Kuala Lumpur 70	M20301|M20301.1 Theiler murine encephalomyelitis virus, complete genome. [29-AUG-2014]	85	242	99.17	8093	1	2.965525763	Theiler's encephalomyelitis virus:no rank
Kuala Lumpur 70	HQ425636|HQ425636.1 Avian leukemia virus isolate SCDY1, complete genome. [29-AUG-2014]	100	169	95.86	7489	1	2.163172653	Avian leukemia virus:no rank
Kuala Lumpur 70	AB261167|AB261167.1 Cucumber mottle virus genomic RNA, complete genome. [29-AUG-2014]	90.40727273	149.7272727	97.59454545	6485	11	24.61063994	Cucumber mottle virus:species
Kuala Lumpur 70	J02275|J02275.1 Minute virus of mice, complete genome. [29-AUG-2014]	97.14	108	97.22	5149	1	2.039230919	Minute virus of mice:no rank
Kuala Lumpur 70	KF214638|KF214638.1 Parvo-like hybrid virus UC11, complete genome. [29-AUG-2014]	100	581	97.07	3637	1	15.50728622	Parvo-like hybrid virus UC11:no rank
Kuala Lumpur 70	KC008572|KC008572.1 UNVERIFIED: Moumouvirus goulette, complete genome. [29-AUG-2014]	90.48	127	99.21	1016844	1	0.012391281	Moumouvirus goulette:no rank
Kuala Lumpur 70	AB089381|AB089381.1 Paprika mild mottle virus complete genome, strain:Japanese. [29-AUG-2014]	100	112	99.11	6524	1	1.701410178	Paprika mild mottle virus:species
Kuala Lumpur 70	X00956|X00956.1 Black beetle virus (BBV) RNA2 sequence for coat protein precursor [29-AUG-2014]	97.06	104	98.08	1399	1	7.290922087	Black beetle virus:species
Kuala Lumpur 70	KF214645|KF214645.1 Parvo-like hybrid virus UC4, complete genome. [29-AUG-2014]	98.285	1236.5	99.82	3638	2	67.86695987	Parvo-like hybrid virus UC4:no rank
Kuala Lumpur 70	HE818438|HE818438.1 Tobacco mosaic virus isolate Pingtan-1, complete genome [29-AUG-2014]	97.81333333	1083.666667	99.65166667	6395	6	94.8084441	Tobacco mosaic virus:species
Kuala Lumpur 70	AY859497|AY859497.1 Pepper mild mottle virus isolate PMMoV-CN, complete genome. [29-AUG-2014]	98.865	154.5	94	6356	2	4.578351164	Pepper mild mottle virus:species
Kuala Lumpur 70	AF308158|AF308158.2 Cactus virus X, complete genome. [29-AUG-2014]	100	137	98.54	6614	1	2.041124887	Cactus virus X:species
Kuala Lumpur 70	M34077|M34077.1 Tobacco mild green mosaic virus, complete genome. [29-AUG-2014]	100	113	99.555	6355	2	3.540519276	Tobacco mild green mosaic virus:species
Kuala Lumpur 70	AB628188|AB628188.1 Rehmannia mosaic virus genomic RNA, complete genome, isolate: Japanese. [29-AU	96.056	128.2	98.298	6395	5	9.851446443	Rehmannia mosaic virus:species
Kuala Lumpur 70	DQ890022|DQ890022.1 Paramecium bursaria Chlorella virus FR483, complete genome. [29-AUG-2014]	85.76333333	138	96.38	321240	3	0.124206201	Paramecium bursaria Chlorella virus FR483:no rank
Kuala Lumpur 70	HF920633|HF920633.1 Invertebrate iridovirus 22 complete genome [29-AUG-2014]	82.69	158	98.73	197693	1	0.078910229	Invertebrate iridovirus 22:species

Kuala Lumpur 70		D12505|D12505.1 Cucumber green mottle mosaic virus RNA, complete genome. [29-AUG-2014]	99.22153846	262.5384615	98.39692308	6424	13	52.5373599	Cucumber green mottle mosaic virus:species
Kuala Lumpur 70		DQ365814|DQ365814.1 Avian leukosis virus strain MQNCSU, complete genome. [29-AUG-2014]	96.265	281.5	99.11	7704	2	7.242990654	Avian leukosis virus:species
Copenhagen University Hospital	56-DNAse	JQ898342|JQ898342.1 Kobuvirus sewage Kathmandu isolate KoV-SewKTM, complete genome. [29-AUG-201	87.126	1791.2	80.538	6939	5	85.58870154	Kobuvirus sewage Kathmandu:no rank
Copenhagen University Hospital	56-DNAse	AF332868|AF332868.1 Tomato mosaic virus isolate Queensland, complete genome. [29-AUG-2014]	99.5775	668.5	99.23	6383	4	41.64186119	Tomato mosaic virus:species
Copenhagen University Hospital	56-DNAse	KF371634|KF371634.1 Faecal-associated gemycircularvirus 8, complete genome. [02-OCT-2014]	84.85	101	98.02	2174	1	4.553817847	Faecal-associated gemycircularvirus 8:species
Copenhagen University Hospital	56-DNAse	KF274499|KF274499.1 Alcelaphine herpesvirus 2 isolate topi-AlHV-2, complete genome. [29-AUG-2014]	91.67	109	99.08	137090	1	0.078780363	Alcelaphine herpesvirus 2:species
Copenhagen University Hospital	56-DNAse	AY150217|AY150217.1 Ambystoma tigrinum stebbensi virus, complete genome. [29-AUG-2014]	80	139	97.12	106332	1	0.12696084	Ambystoma tigrinum stebbensi virus:no rank
Copenhagen University Hospital	56-DNAse	KF738873|KF738873.1 Dragonfly larvae associated circular virus-1 isolate DflaCV-1 NZ-PG11-LD, complete g	70.365	279.5	97.385	2668	2	20.57721139	Dragonfly larvae associated circular virus-1:species
Copenhagen University Hospital	56-DNAse	KJ547631|KJ547631.1 Sewage-associated circular DNA virus-7 isolate SaCV-7 NZ-BS3976-2012, complete g	80	155	96.77	2311	1	6.490696668	Sewage-associated circular DNA virus-7:species
Copenhagen University Hospital	56-DNAse	EF101928|EF101928.1 Acanthocystis turfacea Chlorella virus 1, complete genome. [29-AUG-2014]	74.516	131.4	97.192	288047	5	0.221838797	Acanthocystis turfacea Chlorella virus 1:species
Copenhagen University Hospital	56-DNAse	AJ243571|AJ243571.1 Tomato mosaic virus (K1 strain) complete RNA genome [29-AUG-2014]	99.37333333	897.6666667	99.4	6383	3	42.06485978	Tomato mosaic virus:species
Copenhagen University Hospital	56-DNAse	DQ090005|DQ090005.2 Human papillomavirus type 38b subtype FA125, complete genome. [29-AUG-2014]	99.71	3012.5	88.39	7400	2	73.5	Human papillomavirus type 38b:no rank
Copenhagen University Hospital	56-DNAse	GQ918152|GQ918152.1 Wiseana iridescent virus, complete genome. [29-AUG-2014]	88.89	111	97.3	205791	1	0.052480429	Wiseana iridescent virus:no rank
Copenhagen University Hospital	56-DNAse	AB597230|AB597230.1 Bell pepper endornavirus genomic RNA, complete genome, host: Capsicum annuum c	100	102	100	14727	1	0.692605419	Bell pepper endornavirus:species
Copenhagen University Hospital	56-DNAse	AB078435|AB078435.1 Tobacco mild green mosaic virus complete genome, strain:Japanese. [29-AUG-2014	100	149	98.74	6356	2	4.625550661	Tobacco mild green mosaic virus:species
Copenhagen University Hospital	56-DNAse	DQ355023|DQ355023.1 Bell pepper mottle virus, complete genome. [29-AUG-2014]	89.26	213.5	96.9975	6375	4	13.08235294	Bell pepper mottle virus:species
Copenhagen University Hospital	56-DNAse	M29671|M29671.1 Melon necrotic spot virus (clones pMNS17A and pMNS01A) p29, replicase (p89), p7A, p7	100	121	99.17	4266	1	2.812939522	Melon necrotic spot virus:species
Copenhagen University Hospital	56-DNAse	FJ959081|FJ959081.1 Circovirus-like genome RW-E, complete genome. [29-AUG-2014]	52	306	98.04	2782	1	10.78360891	Circovirus-like genome RW-E:species
Copenhagen University Hospital	56-DNAse	KJ938717|KJ938717.1 Caribou feces-associated gemycircularvirus, complete genome. [14-SEP-2014]	77.845	329.5	98.4875	2232	4	57.25806452	Caribou feces-associated gemycircularvirus:species
Copenhagen University Hospital	56-DNAse	GU244497|GU244497.1 Cafeteria roenbergensis virus BV-PW1, complete genome. [29-AUG-2014]	80	120	100	617453	1	0.019434678	Cafeteria roenbergensis virus BV-PW1:no rank
Copenhagen University Hospital	56-DNAse	KF938901|KF938901.1 UNVERIFIED: Anopheles minimus irodovirus isolate AMIV, complete genome. [29-AUG	46.43	587	98.13	163023	1	0.360685302	Anopheles minimus irodovirus:species
Copenhagen University Hospital	56-DNAse	AF303741|AF303741.1 Chilo iridescent virus complete genome. [29-AUG-2014]	100	102	97.06	212482	1	0.046592182	Invertebrate iridescent virus 6:species
Copenhagen University Hospital	56-DNAse	JF938082|JF938082.1 Bat circovirus ZS/China/2011 isolate YN-BtCV-5, complete genome. [29-AUG-2014]	48.55	625	63.36	1818	1	22.60726073	Bat circovirus ZS/China/2011:species
Copenhagen University Hospital	56-DNAse	KC580640|KC580640.1 Canine bocavirus 3 isolate UCD, complete genome. [29-AUG-2014]	51.02	2854	59.71	5278	1	32.56915498	Canine bocavirus 3:species
Copenhagen University Hospital	56-DNAse	JN704610|JN704610.1 Meles meles fecal virus isolate VS4700006, complete genome. [29-AUG-2014]	73.78	2202	29.155	2198	2	58.78070974	Meles meles fecal virus:species
Copenhagen University Hospital	56-DNAse	V01197|V01197.1 Rous sarcoma virus genome, strain Prague C (Pr-C). [29-AUG-2014]	96.38166667	422.3333333	77.69	9312	6	18.17010309	Rous sarcoma virus:species
Copenhagen University Hospital	56-DNAse	JF755427|JF755427.1 Mouse kobuvirus M-5/USA/2010, complete genome. [29-AUG-2014]	65.98	1594	34.065	8198	2	13.53988778	Mouse kobuvirus M-5/USA/2010:species
Copenhagen University Hospital	56-DNAse	AF225954|AF225954.1 Broad bean wilt virus 2 segment RNA2, complete sequence. [29-AUG-2014]	77.5	122	98.36	3607	1	3.32686443	Broad bean wilt virus 2:species
Copenhagen University Hospital	56-DNAse	M81888|M81888.1 Parvovirus LuIII DNA sequence. [29-AUG-2014]	98.04	155	98.71	5135	1	2.979552093	LuIII virus:no rank
Copenhagen University Hospital	56-DNAse	AB090161|AB090161.1 Theiler's-like virus of rats NGS910 genomic RNA, complete genome. [29-AUG-2014]	91.7075	240.375	98.03875	8021	8	23.56314674	Theiler's-like virus of rats:no rank
Copenhagen University Hospital	56-DNAse	EU376394|EU376394.1 Human TMEV-like cardiovirus, complete genome. [29-AUG-2014]	76.34	280	99.64	7961	1	3.504584851	Human TMEV-like cardiovirus:no rank
Copenhagen University Hospital	56-DNAse	M37980|M37980.1 Avian leukosis virus - RSA, complete genome. [29-AUG-2014]	97.765	660	99.495	7286	2	18.03458688	Avian leukosis virus - RSA:no rank
Copenhagen University Hospital	56-DNAse	KJ577816|KJ577816.1 Porcine stool-associated circular virus 9 isolate FP1, complete genome. [02-SEP-201	72.13	1795	10.19	2564	1	7.137285491	Porcine stool-associated circular virus 9:species
Copenhagen University Hospital	56-DNAse	AY437609|AY437609.1 Watermelon mosaic virus strain WMV-Fr, complete genome. [29-AUG-2014]	97.3	115	96.52	10035	1	1.10612855	Watermelon mosaic virus:species
Copenhagen University Hospital	56-DNAse	KJ194505|KJ194505.1 Human cosavirus isolate Cosavirus Amsterdam 1994, complete genome. [29-AUG-2(	72.97333333	221.3333333	98.44666667	7802	3	8.305562676	Human cosavirus:species
Copenhagen University Hospital	56-DNAse	JQ340310|JQ340310.1 Astrovirus wild boar/WBAstV-1/2011/HUN, complete genome. [29-AUG-2014]	63.06	370	90	6707	1	4.96496198	Astrovirus wild boar/WBAstV-1/2011/HUN:species
Copenhagen University Hospital	56-DNAse	DQ333351|DQ333351.1 Choristoneura occidentalis granulovirus, complete genome. [29-AUG-2014]	92.31	398	19.6	104710	1	0.074491453	Choristoneura occidentalis granulovirus:species
Copenhagen University Hospital	56-DNAse	HQ425636|HQ425636.1 Avian leukemia virus isolate SCDY1, complete genome. [29-AUG-2014]	95.38666667	235.3333333	84.40666667	7489	3	8.252103085	Avian leukemia virus:no rank
Copenhagen University Hospital	56-DNAse	GQ404855|GQ404855.1 Cyclovirus NG14, complete genome. [29-AUG-2014]	73	601	99.83	1795	1	33.25905292	Cyclovirus NG14:species
Copenhagen University Hospital	56-DNAse	GQ401365|GQ401365.1 Ribgrass mosaic virus strain RMV Actinidia-AC isolate M2/A4, complete genome. [2!	96.97	101	98.02	6311	1	1.56868959	Ribgrass mosaic virus:species
Copenhagen University Hospital	56-DNAse	JX185430|JX185430.1 Dragonfly-associated circular virus 1 isolate FL1-2X-2010, complete genome. [29-AU	74.7	252	98.81	2225	1	11.19101124	Dragonfly-associated circular virus 1:species
Copenhagen University Hospital	56-DNAse	KJ158169|KJ158169.1 Rosavirus 2 strain GA7403, complete genome. [29-AUG-2014]	83.73333333	177.6666667	98.4	8931	3	5.878401075	Rosavirus A2:subspecies
Copenhagen University Hospital	56-DNAse	KJ547632|KJ547632.1 Sewage-associated circular DNA virus-8 isolate SaCV-8 NZ-BS4075-2012, complete g	79.07	131	98.47	2577	1	5.005820722	Sewage-associated circular DNA virus-8:species
Copenhagen University Hospital	56-DNAse	JF320811|JF320811.1 Shallot latent virus isolate WA-1, complete genome. [29-AUG-2014]	92.425	101	98.02	8371	2	2.365308804	Shallot latent virus:species
Copenhagen University Hospital	56-DNAse	KC610514|KC610514.1 Scheffersomyces segobiensis virus L isolate NRRL Y-11571, complete genome. [29-	65.23	250	98.395	4590	2	10.71895425	Scheffersomyces segobiensis virus L:species
Copenhagen University Hospital	56-DNAse	AB089381|AB089381.1 Paprika mild mottle virus complete genome, strain:Japanese. [29-AUG-2014]	99.47125	636	99.78125	6524	8	74.69343961	Paprika mild mottle virus:species
Copenhagen University Hospital	56-DNAse	AY037928|AY037928.1 Human endogenous retrovirus K113 complete genome. [29-AUG-2014]	82.22	137	98.54	9472	1	1.425253378	Human endogenous retrovirus K113:no rank
Copenhagen University Hospital	56-DNAse	JQ941880|JQ941880.1 Bovine hungarovirus 1 strain BHUV1/2008/HUN, complete genome. [29-AUG-2014]	79.46	365.25	97.32	7583	12	54.47711987	Bovine hungarovirus 1:no rank
Copenhagen University Hospital	56-DNAse	KF214645|KF214645.1 Parvo-like hybrid virus UC4, complete genome. [29-AUG-2014]	100	591	99.745	3638	2	32.40791644	Parvo-like hybrid virus UC4:no rank
Copenhagen University Hospital	56-DNAse	JX544744|JX544744.1 Murine astrovirus strain STL 2, complete genome. [29-AUG-2014]	66.99	358	99.65	6827	2	10.45847371	Murine astrovirus:species
Copenhagen University Hospital	56-DNAse	AM491609|AM491609.1 Botryotinia fuckeliana partitivirus 1, complete segment 1, genomic RNA [29-AUG-2(	77.55	149	98.66	1793	1	8.198549916	Botryotinia fuckeliana partitivirus 1:species
Copenhagen University Hospital	56-DNAse	KF793927|KF793927.1 Caprine kobuvirus isolate 12Q108, complete genome. [29-AUG-2014]	81.82	133	99.25	8305	1	1.589403974	Caprine kobuvirus:species
Copenhagen University Hospital	56-DNAse	HE818438|HE818438.1 Tobacco mosaic virus isolate Pingtan-1, complete genome [29-AUG-2014]	98.53	114.5	98.935	6395	4	7.083659109	Tobacco mosaic virus:species
Copenhagen University Hospital	56-DNAse	AY859497|AY859497.1 Pepper mild mottle virus isolate PMMoV-CN, complete genome. [29-AUG-2014]	99.008	1270	99.812	6356	5	91.92888609	Pepper mild mottle virus:species
Copenhagen University Hospital	56-DNAse	M34077|M34077.1 Tobacco mild green mosaic virus, complete genome. [29-AUG-2014]	99.57666667	188.6666667	99.52	6355	3	8.874901652	Tobacco mild green mosaic virus:species
Copenhagen University Hospital	56-DNAse	EU815052|EU815052.1 Rat theilovirus 1, complete genome. [29-AUG-2014]	91.16	289.2857143	98.48571429	7996	7	24.08704352	Rat theilovirus 1:no rank
Copenhagen University Hospital	56-DNAse	KJ547633|KJ547633.1 Sewage-associated circular DNA virus-9 isolate SaCV-9 NZ-BS3681-2012, complete g	61.415	2837.5	36.78	2423	2	83.32645481	Sewage-associated circular DNA virus-9:species
Copenhagen University Hospital	56-DNAse	U01060|U01060.1 Saccharomyces cerevisiae virus La, complete genome. [29-AUG-2014]	63.74	582	88.14	4615	1	11.11592633	Saccharomyces cerevisiae virus L-BC (La):species
Copenhagen University Hospital	56-DNAse	HF920637|HF920637.1 Armadillidium vulgare iridescent virus complete genome [25-JUN-2014]	98	151	99.34	220222	1	0.068113086	Armadillidium vulgare iridescent virus:no rank
Copenhagen University Hospital	56-DNAse	DQ890022|DQ890022.1 Paramecium bursaria Chlorella virus FR483, complete genome. [29-AUG-2014]	94.25	123	96.49666667	321240	3	0.111131864	Paramecium bursaria Chlorella virus FR483:no rank
Copenhagen University Hospital	56-DNAse	D12505|D12505.1 Cucumber green mottle mosaic virus RNA, complete genome. [29-AUG-2014]	99.2	6418	99.24	6424	1	99.14383562	Cucumber green mottle mosaic virus:species
Copenhagen University Hospital	56-DNAse	DQ365814|DQ365814.1 Avian leukosis virus strain MQNCSU, complete genome. [29-AUG-2014]	91.718	590.6	99.34	7704	5	37.92834891	Avian leukosis virus:species
Copenhagen University Hospital	56-DNAse	JX185429|JX185429.1 Dragonfly-associated circular virus 2 isolate FL2-5X-2010, complete genome. [29-AU	69.39	152	94.74	2236	1	6.574239714	Dragonfly-associated circular virus 2:species
Copenhagen University Hospital	56-DNAse	M73260|M73260.1 Mastadenovirus h5 gene, complete genome. [29-AUG-2014]	100	179.6666667	98.085	35935	6	2.93863921	Human adenovirus 5:no rank
Copenhagen University Hospital	56-DNAse	JF713713|JF713713.1 Porcine astrovirus 4 strain 35/USA, complete genome. [29-AUG-2014]	84.76	316	99.68	6639	1	4.744690465	Porcine astrovirus 4:species
Copenhagen University Hospital	56-DNAse	AB193726|AB193726.1 Aurantiochytrium single-stranded RNA virus genomic RNA, complete genome. [29-A	73.68	129	88.37	9035	1	1.261759823	Aurantiochytrium single-stranded RNA virus 01:species
Copenhagen University Hospital	56-DNAse	DQ335246|DQ335246.2 Adeno-associated virus-Go.1, complete genome. [29-AUG-2014]	61.34	4374	51.37	4636	1	48.27437446	Adeno-associated virus-Go.1:no rank
Copenhagen University Hospital	56-DNAse	KJ577815|KJ577815.1 Porcine stool-associated circular virus 7 isolate EP3-D, complete genome. [02-SEP-2(	83.304	304.4	78.814	2596	5	39.75346687	Porcine stool-associated circular virus 7:species
Copenhagen University Hospital	56-DNAse	KF477193|KF477193.1 Tomato mottle mosaic virus isolate MX5, complete genome. [29-AUG-2014]	100	148	99.28	6398	3	6.892778993	Tomato mottle mosaic virus:species
Copenhagen University Hospital	-57s	KC977570|KC977570.1 Pandoravirus dulcis, complete genome. [29-AUG-2014]	58.62	764	22.77	1908524	1	0.009116993	Pandoravirus dulcis:species
Copenhagen University Hospital	-57s	JQ898342|JQ898342.1 Kobuvirus sewage Kathmandu isolate KoV-SewKTM, complete genome. [29-AUG-201	86.1275	3243	65.5575	6939	4	99.51001585	Kobuvirus sewage Kathmandu:no rank
Copenhagen University Hospital	-57s	DQ286292|DQ286292.1 Rosy apple aphid virus, complete genome. [29-AUG-2014]	89.13	139	99.28	9992	1	1.381104884	Rosy apple aphid virus:no rank
Copenhagen University Hospital	-57s	AF332868|AF332868.1 Tomato mosaic virus isolate Queensland, complete genome. [29-AUG-2014]	99.525	423.6666667	99.755	6383	6	39.76186746	Tomato mosaic virus:species
Copenhagen University Hospital	-57s	JN648103|JN648103.1 Canine bocavirus 1 isolate Con-161, complete genome. [29-AUG-2014]	88.64	135	97.78	5413	1	2.438573804	Canine bocavirus 1:no rank
Copenhagen University Hospital	-57s	KF738873|KF738873.1 Dragonfly larvae associated circular virus-1 isolate DflaCV-1 NZ-PG11-LD, complete g	60.32	895	84.13	2668	1	27.66116942	Dragonfly larvae associated circular virus-1:species
Copenhagen University Hospital	-57s	EF101928|EF101928.1 Acanthocystis turfacea Chlorella virus 1, complete genome. [29-AUG-2014]	78.09	146.3333333	97.78333333	288047	3	0.148934028	Acanthocystis turfacea Chlorella virus 1:species
Copenhagen University Hospital	-57s	KJ417134|KJ417134.1 Porcine stool-associated circular virus 4 isolate CP2, complete genome. [29-AUG-201	89.81	157.25	97.3625	2980	4	20.63758389	Porcine stool-associated circular virus 4:species
Copenhagen University Hospital	-57s	JX645345|JX645345.1 Rat minute virus isolate NTU2, complete genome. [29-AUG-2014]	95	182	98.9	4780	1	3.765690377	Rat minute virus:species
Copenhagen University Hospital	-57s	AJ243571|AJ243571.1 Tomato mosaic virus (K1 strain) complete RNA genome [29-AUG-2014]	99.735	669.5	99.415	6383	2	20.91493028	Tomato mosaic virus:species
Copenhagen University Hospital	-57s	GQ918152|GQ918152.1 Wiseana iridescent virus, complete genome. [29-AUG-2014]	82.5	127	94.49	205791	1	0.058311588	Wiseana iridescent virus:no rank
Copenhagen University Hospital	-57s	DQ355023|DQ355023.1 Bell pepper mottle virus, complete genome. [29-AUG-2014]	93.918	281.1	98.472	6375	10	42.91764706	Bell pepper mottle virus:species
Copenhagen University Hospital	-57s	M29671|M29671.1 Melon necrotic spot virus (clones pMNS17A and pMNS01A) p29, replicase (p89), p7A, p7	96.72	119.6	98.31	4266	5	13.40834505	Melon necrotic spot virus:species
Copenhagen University Hospital	-57s	KJ938717|KJ938717.1 Caribou feces-associated gemycircularvirus, complete genome. [14-SEP-2014]	78.338	260.8	95.402	2232	5	56.58602151	Caribou feces-associated gemycircularvirus:species
Copenhagen University Hospital	-57s	GU244497|GU244497.1 Cafeteria roenbergensis virus BV-PW1, complete genome. [29-AUG-2014]	91.11	492	26.83	617453	1	0.021864012	Cafeteria roenbergensis virus BV-PW1:no rank
Copenhagen University Hospital	-57s	AF033810|AF033810.1 Fujinami sarcoma virus, complete genome. [29-AUG-2014]	91.67	220	65.45	4788	1	3.007518797	Fujinami sarcoma virus:species
Copenhagen University Hospital	-57s	AY388617|AY388617.1 Bovine adeno-associated virus, complete genome. [29-AUG-2014]	67.11	1093	61.48	4693	1	14.3831238	Bovine adeno-associated virus:no rank
Copenhagen University Hospital	-57s	JX185418|JX185418.1 Dragonfly cyclicusvirus isolate FL1-NZ37-2010, complete genome. [29-AUG-2014]	50	757	23.78	1831	1	8.847624249	Dragonfly cyclicusvirus:species
Copenhagen University Hospital	-57s	JF938082|JF938082.1 Bat circovirus ZS/China/2011 isolate YN-BtCV-5, complete genome. [29-AUG-2014]	81.43	212	99.06	1818	1	11.55115512	Bat circovirus ZS/China/2011:species
Copenhagen University Hospital	-57s	KC580640|KC580640.1 Canine bocavirus 3 isolate UCD, complete genome. [29-AUG-2014]	67.12	3587	18.32	5278	1	12.44789693	Canine bocavirus 3:species
Copenhagen University Hospital	-57s	EF198242|EF198242.1 Marine RNA virus JP-B, complete genome. [29-AUG-2014]	43.6	4967	32.31	8926	1	18.04839794	Marine RNA virus JP-B:species
Copenhagen University Hospital	-57s	JN704610|JN704610.1 Meles meles fecal virus isolate VS4700006, complete genome. [29-AUG-2014]	70.33	1105.666667	53.29	2198	3	82.39308462	Meles meles fecal virus:species
Copenhagen University Hospital	-57s	V01197|V01197.1 Rous sarcoma virus genome, strain Prague C (Pr-C). [29-AUG-2014]	91.456	398.8	84.63	9312	5	16.63445017	Rous sarcoma virus:species
Copenhagen University Hospital	-57s	AY509927|AY509927.1 Pepino mosaic virus strain US2, complete genome. [29-AUG-2014]	97.61647059	137.6470588	98.77764706	6410	17	35.5850234	Pepino mosaic virus:species
Copenhagen University Hospital	-57s	M81888|M81888.1 Parvovirus LuIII DNA sequence. [29-AUG-2014]	94.49	164.5	99.395	5135	2	6.368062317	LuIII virus:no rank
Copenhagen University Hospital	-57s	KC991097|KC991097.1 Solenopsis invicta densovirus isolate SiDNV-Arg, complete genome. [29-AUG-2014]	75	145	99.31	5280	1	2.727272727	Solenopsis invicta densovirus:species
Copenhagen University Hospital	-57s	AB090161|AB090161.1 Theiler's-like virus of rats NGS910 genomic RNA, complete genome. [29-AUG-2014]	88.058	598	94.398	8021	5	35.41952375	Theiler's-like virus of rats:no rank
Copenhagen University Hospital	-57s	U44978|U44978.1 Porcine parvovirus strain Kresse, complete genome. [29-AUG-2014]	100	110	98.18	4948	1	2.182700081	Porcine parvovirus:no rank
Copenhagen University Hospital	-57s	M37980|M37980.1 Avian leukosis virus - RSA, complete genome. [29-AUG-2014]	98.3	600	99.75	7286	2	16.4287675	Avian leukosis virus - RSA:no rank
Copenhagen University Hospital	-57s	JQ814851|JQ814851.1 Miniopterus schreibersii picornavirus 1, complete genome. [29-AUG-2014]	73.91	141	97.87	8468	1	1.62966462	Miniopterus schreibersii picornavirus 1:no rank
Copenhagen University Hospital	-57s	JQ340310|JQ340310.1 Astrovirus wild boar/WBAstV-1/2011/HUN, complete genome. [29-AUG-2014]	54.575	526.5	96.77	6707	2	12.39004026	Astrovirus wild boar/WBAstV-1/2011/HUN:species
Copenhagen University Hospital	-57s	X01457|X01457.1 Parvovirus h-1, complete genome [29-AUG-2014]	100	160	97.5	5176	1	3.013910355	H-1 parvovirus:no rank
Copenhagen University Hospital	-57s	HQ425636|HQ425636.1 Avian leukemia virus isolate SCDY1, complete genome. [29-AUG-2014]	95.285	766.5	99.01	7489	2	20.22967018	Avian leukemia virus:no rank
Copenhagen University Hospital	-57s	KJ158169|KJ158169.1 Rosavirus 2 strain GA7403, complete genome. [29-AUG-2014]	70.77	702.3333333	98.11	8931	6	42.3580786	Rosavirus A2:subspecies
Copenhagen University Hospital	-57s	KJ547632|KJ547632.1 Sewage-associated circular DNA virus-8 isolate SaCV-8 NZ-BS4075-2012, complete g	67.14	214	98.13	2577	1	8.149010477	Sewage-associated circular DNA virus-8:species
Copenhagen University Hospital	-57s	AB089381|AB089381.1 Paprika mild mottle virus complete genome, strain:Japanese. [29-AUG-2014]	99.32272727	513.7272727	99.42272727	6524	11	82.48007357	Paprika mild mottle virus:species
Copenhagen University Hospital	-57s	JQ941880|JQ941880.1 Bovine hungarovirus 1 strain BHUV1/2008/HUN, complete genome. [29-AUG-2014]	78.09384615	255.3846154	97.20538462	7583	13	41.93590927	Bovine hungarovirus 1:no rank
Copenhagen University Hospital	-57s	KF214645|KF214645.1 Parvo-like hybrid virus UC4, complete genome. [29-AUG-2014]	92.31	120	97.5	3638	1	3.216052776	Parvo-like hybrid virus UC4:no rank
Copenhagen University Hospital	-57s	KF738874|KF738874.1 Dragonfly larvae associated circular virus-2 isolate DflaCV-2 NZ-PG8-LS, complete ge	75	750	35.2	2398	1	11.00917431	Dragonfly larvae associated circular virus-2:species
Copenhagen University Hospital	-57s	JX544744|JX544744.1 Murine astrovirus strain STL 2, complete genome. [29-AUG-2014]	49.68	7403	20.81	6827	2	41.49699722	Murine astrovirus:species
Copenhagen University Hospital	-57s	GQ404845|GQ404845.1 Cyclovirus PK5034, complete genome. [29-AUG-2014]	55.56	243	100	1780	1	13.48314607	Cyclovirus PK5034:species
Copenhagen University Hospital	-57s	HE818438|HE818438.1 Tobacco mosaic virus isolate Pingtan-1, complete genome [29-AUG-2014]	100	166	99.4	6395	1	2.580140735	Tobacco mosaic virus:species
Copenhagen University Hospital	-57s	AY859497|AY859497.1 Pepper mild mottle virus isolate PMMoV-CN, complete genome. [29-AUG-2014]	99.64142857	729.7142857	99.50428571	6356	7	76.65198238	Pepper mild mottle virus:species
Copenhagen University Hospital	-57s	KJ547620|KJ547620.1 Sewage-associated circular DNA virus-1 isolate SaCV-1 NZ-BS3349-2012, complete g	78.26	181	76.24	2777	1	4.96939143	Sewage-associated circular DNA virus-1:species
Copenhagen University Hospital	-57s	M34077|M34077.1 Tobacco mild green mosaic virus, complete genome. [29-AUG-2014]	100	152	98.68	6355	1	2.360346184	Tobacco mild green mosaic virus:species
Copenhagen University Hospital	-57s	JQ814852|JQ814852.1 Ia io picornavirus 1, complete genome. [29-AUG-2014]	52.06	944	98.52	7543	1	12.36908392	Ia io picornavirus 1:species
Copenhagen University Hospital	-57s	EU815052|EU815052.1 Rat theilovirus 1, complete genome. [29-AUG-2014]	94.466	790.4	98.644	7996	5	49.03701851	Rat theilovirus 1:no rank
Copenhagen University Hospital	-57s	KJ547633|KJ547633.1 Sewage-associated circular DNA virus-9 isolate SaCV-9 NZ-BS3681-2012, complete g	58.33	980	19.9	2423	1	8.543128353	Sewage-associated circular DNA virus-9:species
Copenhagen University Hospital	-57s	DQ890022|DQ890022.1 Paramecium bursaria Chlorella virus FR483, complete genome. [29-AUG-2014]	80	147	81.63	321240	1	0.037355248	Paramecium bursaria Chlorella virus FR483:no rank
Copenhagen University Hospital	-57s	S73580|S73580.1 putative polymerase...capsid protein [potato aucuba mosaic potexvirus PAMV, Genomic RI	76.47	158	96.84	7059	1	2.167445814	Potato aucuba mosaic virus:species
Copenhagen University Hospital	-57s	D12505|D12505.1 Cucumber green mottle mosaic virus RNA, complete genome. [29-AUG-2014]	97.385	1604.25	98.9425	6424	4	95.03424658	Cucumber green mottle mosaic virus:species
Copenhagen University Hospital	-57s	DQ365814|DQ365814.1 Avian leukosis virus strain MQNCSU, complete genome. [29-AUG-2014]	97.245	305	98.83	7704	2	7.827102804	Avian leukosis virus:species
Copenhagen University Hospital	-57s	M73260|M73260.1 Mastadenovirus h5 gene, complete genome. [29-AUG-2014]	100	121	99.17	35935	1	0.333936274	Human adenovirus 5:no rank
Copenhagen University Hospital	-57s	JF713713|JF713713.1 Porcine astrovirus 4 strain 35/USA, complete genome. [29-AUG-2014]	72.01	394	99.76	6639	2	11.8391324	Porcine astrovirus 4:species
Copenhagen University Hospital	-57s	DQ335246|DQ335246.2 Adeno-associated virus-Go.1, complete genome. [29-AUG-2014]	61.44	2304	96.09	4636	1	47.62726488	Adeno-associated virus-Go.1:no rank
Copenhagen University Hospital	-57s	KJ577815|KJ577815.1 Porcine stool-associated circular virus 7 isolate EP3-D, complete genome. [02-SEP-2(	54.77	1618	65.45	2596	1	41.94915254	Porcine stool-associated circular virus 7:species
Copenhagen University Hospital	-57s	KF477193|KF477193.1 Tomato mottle mosaic virus isolate MX5, complete genome. [29-AUG-2014]	96.155	205.5	100	6398	2	6.423882463	Tomato mottle mosaic virus:species
Copenhagen University Hospital	64-67 po	JQ898342|JQ898342.1 Kobuvirus sewage Kathmandu isolate KoV-SewKTM, complete genome. [29-AUG-201	94.329375	230.875	98.81125	6939	16	51.75097276	Kobuvirus sewage Kathmandu:no rank
Copenhagen University Hospital	64-67 po	M20301|M20301.1 Theiler murine encephalomyelitis virus, complete genome. [29-AUG-2014]	84	154	97.4	8093	1	1.853453602	Theiler's encephalomyelitis virus:no rank
Copenhagen University Hospital	64-67 po	AF332868|AF332868.1 Tomato mosaic virus isolate Queensland, complete genome. [29-AUG-2014]	99.63333333	969.3333333	99.88	6383	3	44.97885007	Tomato mosaic virus:species
Copenhagen University Hospital	64-67 po	KF831027|KF831027.1 Feline kobuvirus strain FK-13, complete genome. [29-AUG-2014]	100	125	98.4	8219	1	1.496532425	Feline kobuvirus:no rank
Copenhagen University Hospital	64-67 po	AJ417701|AJ417701.1 Tomato mosaic virus camellia strain, complete genome [29-AUG-2014]	100	531	99.44	6383	1	8.271972427	Tomato mosaic virus:species
Copenhagen University Hospital	64-67 po	X02396|X02396.1 Black beetle virus (BBV) RNA1 and RNA3 for protein A and B [29-AUG-2014]	100	112	99.11	3106	1	3.573728268	Black beetle virus:species
Copenhagen University Hospital	64-67 po	KJ158169|KJ158169.1 Rosavirus 2 strain GA7403, complete genome. [29-AUG-2014]	69.81	162	98.15	8931	1	1.780315754	Rosavirus A2:subspecies
Copenhagen University Hospital	64-67 po	AJ243571|AJ243571.1 Tomato mosaic virus (K1 strain) complete RNA genome [29-AUG-2014]	99.67666667	710.3333333	99.73333333	6383	3	33.32288892	Tomato mosaic virus:species
Copenhagen University Hospital	64-67 po	AB089381|AB089381.1 Paprika mild mottle virus complete genome, strain:Japanese. [29-AUG-2014]	99.854	438.8	99.394	6524	10	65.11342735	Paprika mild mottle virus:species
Copenhagen University Hospital	64-67 po	JQ941880|JQ941880.1 Bovine hungarovirus 1 strain BHUV1/2008/HUN, complete genome. [29-AUG-2014]	78.13166667	615.1666667	99.41166667	7583	6	47.27680338	Bovine hungarovirus 1:no rank
Copenhagen University Hospital	64-67 po	KF214645|KF214645.1 Parvo-like hybrid virus UC4, complete genome. [29-AUG-2014]	97.96	590	99.66	3638	1	16.16272677	Parvo-like hybrid virus UC4:no rank
Copenhagen University Hospital	64-67 po	AB078435|AB078435.1 Tobacco mild green mosaic virus complete genome, strain:Japanese. [29-AUG-2014	95.35	179.5	98.6	6356	2	5.569540592	Tobacco mild green mosaic virus:species
Copenhagen University Hospital	64-67 po	DQ355023|DQ355023.1 Bell pepper mottle virus, complete genome. [29-AUG-2014]	90.726	193.6	98.7	6375	5	15.01176471	Bell pepper mottle virus:species
Copenhagen University Hospital	64-67 po	FJ959081|FJ959081.1 Circovirus-like genome RW-E, complete genome. [29-AUG-2014]	84.09	139	94.96	2782	1	4.744787922	Circovirus-like genome RW-E:species
Copenhagen University Hospital	64-67 po	X56019|X56019.1 Theilers murine encephalomyelitis virus (TMEV) complete genome [29-AUG-2014]	84.72	220	98.18	8101	1	2.666337489	Theiler's encephalomyelitis virus (STRAIN GDVII):no rank
Copenhagen University Hospital	64-67 po	HE818438|HE818438.1 Tobacco mosaic virus isolate Pingtan-1, complete genome [29-AUG-2014]	97.77666667	312.5833333	99.03416667	6395	12	56.9820172	Tobacco mosaic virus:species
Copenhagen University Hospital	64-67 po	AY859497|AY859497.1 Pepper mild mottle virus isolate PMMoV-CN, complete genome. [29-AUG-2014]	99.73125	248.125	99.475	6356	8	30.53807426	Pepper mild mottle virus:species
Copenhagen University Hospital	64-67 po	M34077|M34077.1 Tobacco mild green mosaic virus, complete genome. [29-AUG-2014]	98.485	108.5	98.15	6355	2	3.351691581	Tobacco mild green mosaic virus:species
Copenhagen University Hospital	64-67 po	AB040749|AB040749.1 Aichi virus genomic RNA, complete genome. [29-AUG-2014]	92.73	167	98.8	8280	1	1.992753623	Aichivirus A:species
Copenhagen University Hospital	64-67 po	EU815052|EU815052.1 Rat theilovirus 1, complete genome. [29-AUG-2014]	93.22	397	99.72	7996	2	9.904952476	Rat theilovirus 1:no rank
Copenhagen University Hospital	64-67 po	FR878069|FR878069.1 Tobacco mosaic virus strain Ohio V, complete genome, genomic RNA [29-AUG-2014	97.06	105	97.14	6392	1	1.595744681	Tobacco mosaic virus strain Ohio V:no rank
Copenhagen University Hospital	64-67 po	AB628188|AB628188.1 Rehmannia mosaic virus genomic RNA, complete genome, isolate: Japanese. [29-AU	97.62	116.3333333	98.81333333	6395	3	5.394839719	Rehmannia mosaic virus:species
Copenhagen University Hospital	64-67 po	V01197|V01197.1 Rous sarcoma virus genome, strain Prague C (Pr-C). [29-AUG-2014]	91.32333333	194	90.87	9312	3	5.476804124	Rous sarcoma virus:species
Copenhagen University Hospital	64-67 po	JF755427|JF755427.1 Mouse kobuvirus M-5/USA/2010, complete genome. [29-AUG-2014]	79.68	389.5	93.145	8198	2	8.819224201	Mouse kobuvirus M-5/USA/2010:species
Copenhagen University Hospital	64-67 po	D12505|D12505.1 Cucumber green mottle mosaic virus RNA, complete genome. [29-AUG-2014]	97.4175	636.5	90.75916667	6424	12	94.76961395	Cucumber green mottle mosaic virus:species
Copenhagen University Hospital	64-67 po	AY509927|AY509927.1 Pepino mosaic virus strain US2, complete genome. [29-AUG-2014]	97.87	143	98.6	6410	1	2.199687988	Pepino mosaic virus:species
Copenhagen University Hospital	64-67 po	DQ365814|DQ365814.1 Avian leukosis virus strain MQNCSU, complete genome. [29-AUG-2014]	100	168	98.21	7704	1	2.141744548	Avian leukosis virus:species
Copenhagen University Hospital	64-67 po	KC991097|KC991097.1 Solenopsis invicta densovirus isolate SiDNV-Arg, complete genome. [29-AUG-2014]	84.5	111.5	95.405	5280	2	4.034090909	Solenopsis invicta densovirus:species
Copenhagen University Hospital	64-67 po	AB090161|AB090161.1 Theiler's-like virus of rats NGS910 genomic RNA, complete genome. [29-AUG-2014]	84.09666667	322	99.02333333	8021	3	11.85637701	Theiler's-like virus of rats:no rank
Copenhagen University Hospital	64-67 po	EF165067|EF165067.2 Saffold virus, complete genome. [29-AUG-2014]	68.18	402	98.51	8115	1	4.879852126	Saffold virus:no rank
Copenhagen University Hospital	64-67 po	JF729316|JF729316.1 Rabbit astrovirus TN/2208/2010, complete genome. [29-AUG-2014]	65.57	194	94.33	7353	1	2.48878009	Rabbit astrovirus TN/2208/2010:species
Copenhagen University Hospital	64-67 po	M37980|M37980.1 Avian leukosis virus - RSA, complete genome. [29-AUG-2014]	55.46	352	99.72	7286	1	4.570409004	Avian leukosis virus - RSA:no rank
Copenhagen University Hospital	64-67 po	KF477193|KF477193.1 Tomato mottle mosaic virus isolate MX5, complete genome. [29-AUG-2014]	96.238	248.8	99.03	6398	5	18.41200375	Tomato mottle mosaic virus:species
Kuala Lumpur 75ps-76 v2		JQ898342|JQ898342.1 Kobuvirus sewage Kathmandu isolate KoV-SewKTM, complete genome. [29-AUG-201	93.272	1096.8	99.524	6939	5	77.25897103	Kobuvirus sewage Kathmandu:no rank

Kuala Lumpur 75ps-76 v2	AF332868|AF332868.1 Tomato mosaic virus isolate Queensland, complete genome. [29-AUG-2014]	100	409.5	99.575	6383	2	12.78395739	Tomato mosaic virus:species
Kuala Lumpur 75ps-76 v2	KF831027|KF831027.1 Feline kobuvirus strain FK-13, complete genome. [29-AUG-2014]	87.04	670	72.54	8219	1	5.913128118	Feline kobuvirus:no rank
Kuala Lumpur 75ps-76 v2	KF371634|KF371634.1 Faecal-associated gemycircularvirus 8, complete genome. [02-OCT-2014]	84.91	163	97.55	2174	1	7.313707452	Faecal-associated gemycircularvirus 8:species
Kuala Lumpur 75ps-76 v2	FJ007373|FJ007373.1 Enterovirus J strain POo-1, complete genome. [29-AUG-2014]	48.04	625	96.48	7355	1	8.239292998	Enterovirus J:species
Kuala Lumpur 75ps-76 v2	X02396|X02396.1 Black beetle virus (BBV) RNA1 and RNA3 for protein A and B [29-AUG-2014]	95.892	538.6	99.436	3106	5	83.54797167	Black beetle virus:species
Kuala Lumpur 75ps-76 v2	AJ243571|AJ243571.1 Tomato mosaic virus (K1 strain) complete RNA genome [29-AUG-2014]	99.02	918	100	6383	1	14.38195206	Tomato mosaic virus:species
Kuala Lumpur 75ps-76 v2	KJ577817|KJ577817.1 Porcine stool-associated circular virus 8 isolate GP2, complete genome. [02-SEP-201	55.08	425	83.29	2477	1	14.29148163	Porcine stool-associated circular virus 8:species
Kuala Lumpur 75ps-76 v2	AB078435|AB078435.1 Tobacco mild green mosaic virus complete genome, strain:Japanese. [29-AUG-2014	97.515	2727.5	99.74	6356	2	85.76148521	Tobacco mild green mosaic virus:species
Kuala Lumpur 75ps-76 v2	DQ355023|DQ355023.1 Bell pepper mottle virus, complete genome. [29-AUG-2014]	88.4375	169.75	99.245	6375	4	10.58823529	Bell pepper mottle virus:species
Kuala Lumpur 75ps-76 v2	U75930|U75930.2 Orgyia pseudotsugata MNPV, complete genome. [29-AUG-2014]	95.24	103	61.17	131995	1	0.047729081	Orgyia pseudotsugata multiple nucleopolyhedrovirus:species
Kuala Lumpur 75ps-76 v2	KJ938717|KJ938717.1 Caribou feces-associated gemycircularvirus, complete genome. [14-SEP-2014]	80.182	335.4	88.198	2232	5	60.88709677	Caribou feces-associated gemycircularvirus:species
Kuala Lumpur 75ps-76 v2	AY388617|AY388617.1 Bovine adeno-associated virus, complete genome. [29-AUG-2014]	79.63	164	98.78	4693	1	3.451949712	Bovine adeno-associated virus:no rank
Kuala Lumpur 75ps-76 v2	AB040749|AB040749.1 Aichi virus genomic RNA, complete genome. [29-AUG-2014]	64.29	478	77.2	8280	1	4.420289855	Aichivirus A:species
Kuala Lumpur 75ps-76 v2	EU082131|EU082131.1 Black raspberry virus F, complete genome. [29-AUG-2014]	48.78	497	94.16	5077	1	9.572582234	Black raspberry virus F:species
Kuala Lumpur 75ps-76 v2	FR878069|FR878069.1 Tobacco mosaic virus strain Ohio V, complete genome, genomic RNA [29-AUG-2014	100	103	96.12	6392	1	1.548811014	Tobacco mosaic virus strain Ohio V:no rank
Kuala Lumpur 75ps-76 v2	JF938082|JF938082.1 Bat circovirus ZS/China/2011 isolate YN-BtCV-5, complete genome. [29-AUG-2014]	68.61	651	99.54	1818	1	36.79867987	Bat circovirus ZS/China/2011:species
Kuala Lumpur 75ps-76 v2	KC580640|KC580640.1 Canine bocavirus 3 isolate UCD, complete genome. [29-AUG-2014]	51.02	2977	57.24	5278	1	32.56915498	Canine bocavirus 3:species
Kuala Lumpur 75ps-76 v2	JN704610|JN704610.1 Meles meles fecal virus isolate VS4700006, complete genome. [29-AUG-2014]	80.82	512.5	72.18	2198	2	26.06915378	Meles meles fecal virus:species
Kuala Lumpur 75ps-76 v2	V01197|V01197.1 Rous sarcoma virus genome, strain Prague C (Pr-C). [29-AUG-2014]	93.90714286	182.2857143	93.69428571	9312	7	12.82216495	Rous sarcoma virus:species
Kuala Lumpur 75ps-76 v2	JF755427|JF755427.1 Mouse kobuvirus M-5/USA/2010, complete genome. [29-AUG-2014]	74.49	1586	91.36	8198	1	18.04098561	Mouse kobuvirus M-5/USA/2010:species
Kuala Lumpur 75ps-76 v2	JQ692586|JQ692586.1 Feline bocavirus strain HK797U, complete genome. [29-AUG-2014]	87.93	174	100	5179	1	3.359721954	Feline bocavirus:no rank
Kuala Lumpur 75ps-76 v2	KC991097|KC991097.1 Solenopsis invicta densovirus isolate SiDNV-Arg, complete genome. [29-AUG-2014]	82.0225	129.75	94.12	5280	4	9.261363636	Solenopsis invicta densovirus:species
Kuala Lumpur 75ps-76 v2	AB090161|AB090161.1 Theiler's-like virus of rats NGS910 genomic RNA, complete genome. [29-AUG-2014]	91.035	641	98.3475	8021	8	61.37638698	Theiler's-like virus of rats:no rank
Kuala Lumpur 75ps-76 v2	JQ037754|JQ037754.1 Artibeus jamaicensis parvovirus 1, complete genome. [29-AUG-2014]	79.49	119	98.32	4595	1	2.546245919	Artibeus jamaicensis parvovirus 1:species
Kuala Lumpur 75ps-76 v2	EU376394|EU376394.1 Human TMEV-like cardiovirus, complete genome. [29-AUG-2014]	81.05	477.3333333	98.99333333	7961	3	16.65619897	Human TMEV-like cardiovirus:no rank
Kuala Lumpur 75ps-76 v2	GU991616|GU991616.1 Providence virus isolate vFLM1, complete genome. [29-AUG-2014]	76.36	167	98.8	6155	1	2.68074736	Providence virus:species
Kuala Lumpur 75ps-76 v2	M20301|M20301.1 Theiler murine encephalomyelitis virus, complete genome. [29-AUG-2014]	67.09	1174	99.66	8093	1	14.53107624	Theiler's encephalomyelitis virus:no rank
Kuala Lumpur 75ps-76 v2	JQ340310|JQ340310.1 Astrovirus wild boar/WBAstV-1/2011/HUN, complete genome. [29-AUG-2014]	79.03	157.5	98.14	6707	2	4.607126882	Astrovirus wild boar/WBAstV-1/2011/HUN:species
Kuala Lumpur 75ps-76 v2	HQ425636|HQ425636.1 Avian leukemia virus isolate SCDY1, complete genome. [29-AUG-2014]	97.68	190.5	99.23	7489	2	5.047402858	Avian leukemia virus:no rank
Kuala Lumpur 75ps-76 v2	JX185430|JX185430.1 Dragonfly-associated circular virus 1 isolate FL1-2X-2010, complete genome. [29-AU	80.85	142	99.3	2225	1	6.337078652	Dragonfly-associated circular virus 1:species
Kuala Lumpur 75ps-76 v2	KJ158169|KJ158169.1 Rosavirus 2 strain GA7403, complete genome. [29-AUG-2014]	83.165	171.75	91.8725	8931	4	7.05408129	Rosavirus A2:subspecies
Kuala Lumpur 75ps-76 v2	KC610514|KC610514.1 Scheffersomyces segobiensis virus L isolate NRRL Y-11571, complete genome. [29-	48.66	4034	52.13	4590	1	45.68627451	Scheffersomyces segobiensis virus L:species
Kuala Lumpur 75ps-76 v2	FJ966084|FJ966084.1 Influenza A virus (A/California/04/2009(H1N1)) segment 6 neuraminidase (NA) gen	100	124	99.19	1410	1	8.723404255	Influenza A virus (A/California/04/2009(H1N1)):no rank
Kuala Lumpur 75ps-76 v2	AB089381|AB089381.1 Paprika mild mottle virus complete genome, strain:Japanese. [29-AUG-2014]	99.48333333	391.1666667	99.28583333	6524	12	67.84181484	Paprika mild mottle virus:species
Kuala Lumpur 75ps-76 v2	AY037928|AY037928.1 Human endogenous retrovirus K113 complete genome. [29-AUG-2014]	97.73	133	99.25	9472	1	1.393581081	Human endogenous retrovirus K113:no rank
Kuala Lumpur 75ps-76 v2	X00956|X00956.1 Black beetle virus (BBV) RNA2 sequence for coat protein precursor [29-AUG-2014]	94.335	636.5	99.42	1399	2	90.06433167	Black beetle virus:species
Kuala Lumpur 75ps-76 v2	JQ941880|JQ941880.1 Bovine hungarovirus 1 strain BHUV1/2008/HUN, complete genome. [29-AUG-2014]	71.725	402.5	96.09	7583	6	29.90900699	Bovine hungarovirus 1:no rank
Kuala Lumpur 75ps-76 v2	KF214645|KF214645.1 Parvo-like hybrid virus UC4, complete genome. [29-AUG-2014]	97.78	813	99.63	3638	1	22.26498076	Parvo-like hybrid virus UC4:no rank
Kuala Lumpur 75ps-76 v2	AM491609|AM491609.1 Botryotinia fuckeliana partitivirus 1, complete segment 1, genomic RNA [29-AUG-2	64.005	907	95.1	1793	2	90.51868377	Botryotinia fuckeliana partitivirus 1:species
Kuala Lumpur 75ps-76 v2	X56019|X56019.1 Theilers murine encephalomyelitis virus (TMEV) complete genome [29-AUG-2014]	79.07	1007.333333	83.47666667	8101	3	26.70040736	Theiler's encephalomyelitis virus (STRAIN GDVII):no rank
Kuala Lumpur 75ps-76 v2	HE818438|HE818438.1 Tobacco mosaic virus isolate Pingtan-1, complete genome [29-AUG-2014]	99.24	301	98.86666667	6395	9	40.10946052	Tobacco mosaic virus:species
Kuala Lumpur 75ps-76 v2	AY859497|AY859497.1 Pepper mild mottle virus isolate PMMoV-CN, complete genome. [29-AUG-2014]	98.17916667	386.8333333	99.045	6356	12	68.48646948	Pepper mild mottle virus:species
Kuala Lumpur 75ps-76 v2	M34077|M34077.1 Tobacco mild green mosaic virus, complete genome. [29-AUG-2014]	96.25	802	99.88	6355	1	12.60424862	Tobacco mild green mosaic virus:species
Kuala Lumpur 75ps-76 v2	EU815052|EU815052.1 Rat theilovirus 1, complete genome. [29-AUG-2014]	94.07666667	344.3333333	99.49666667	7996	3	12.86893447	Rat theilovirus 1:no rank
Kuala Lumpur 75ps-76 v2	FJ966082|FJ966082.1 Influenza A virus (A/California/04/2009(H1N1)) segment 4 hemagglutinin (HA) gene	96.23	160	99.38	1701	1	9.347442681	Influenza A virus (A/California/04/2009(H1N1)):no rank
Kuala Lumpur 75ps-76 v2	AB628188|AB628188.1 Rehmannia mosaic virus genomic RNA, complete genome, isolate: Japanese. [29-AU	100	132	98.8425	6395	4	8.146989836	Rehmannia mosaic virus:species
Kuala Lumpur 75ps-76 v2	HF920633|HF920633.1 Invertebrate iridovirus 22 complete genome [29-AUG-2014]	93.06	136	98.05	197693	2	0.135057893	Invertebrate iridovirus 22:species
Kuala Lumpur 75ps-76 v2	D12505|D12505.1 Cucumber green mottle mosaic virus RNA, complete genome. [29-AUG-2014]	98.03090909	168.0909091	98.54272727	6424	11	28.05105853	Cucumber green mottle mosaic virus:species
Kuala Lumpur 75ps-76 v2	JF713713|JF713713.1 Porcine astrovirus 4 strain 35/USA, complete genome. [29-AUG-2014]	79.12333333	200.6666667	88.03333333	6639	3	7.591504745	Porcine astrovirus 4:species
Kuala Lumpur 75ps-76 v2	DQ335246|DQ335246.2 Adeno-associated virus-Go.1, complete genome. [29-AUG-2014]	67.43666667	901.3333333	81.5	4636	3	45.10353753	Adeno-associated virus-Go.1:no rank
Kuala Lumpur 75ps-76 v2	EF165067|EF165067.2 Saffold virus, complete genome. [29-AUG-2014]	86	301	99.67	8115	1	3.696857671	Saffold virus:no rank
Kuala Lumpur 75ps-76 v2	KF477193|KF477193.1 Tomato mottle mosaic virus isolate MX5, complete genome. [29-AUG-2014]	98.235	196	99.27	6398	2	6.095654892	Tomato mottle mosaic virus:species
Kuala Langat 77	HF679134|HF679134.1 Mythimna separata entomopoxvirus 'L', complete genome [29-AUG-2014]	95	121	99.17	281182	1	0.042676985	Mythimna separata entomopoxvirus 'L':species
Kuala Langat 77	JQ898342|JQ898342.1 Kobuvirus sewage Kathmandu isolate KoV-SewKTM, complete genome. [29-AUG-201	93.38833333	276.75	98.765	6939	12	47.21141375	Kobuvirus sewage Kathmandu:no rank
Kuala Langat 77	AF332868|AF332868.1 Tomato mosaic virus isolate Queensland, complete genome. [29-AUG-2014]	99.658	620.8	99.214	6383	5	46.90584365	Tomato mosaic virus:species
Kuala Langat 77	AY150217|AY150217.1 Ambystoma tigrinum stebbensi virus, complete genome. [29-AUG-2014]	59.57	284	99.3	106332	1	0.265207087	Ambystoma tigrinum stebbensi virus:no rank
Kuala Langat 77	X02396|X02396.1 Black beetle virus (BBV) RNA1 and RNA3 for protein A and B [29-AUG-2014]	100	120	100	3106	1	3.863490019	Black beetle virus:species
Kuala Langat 77	AJ243571|AJ243571.1 Tomato mosaic virus (K1 strain) complete RNA genome [29-AUG-2014]	98.16	380.5	99.31	6383	2	11.84396052	Tomato mosaic virus:species
Kuala Langat 77	GQ918152|GQ918152.1 Wiseana iridescent virus, complete genome. [29-AUG-2014]	80.055	127	94.26	205791	2	0.115165386	Wiseana iridescent virus:no rank
Kuala Langat 77	AB078435|AB078435.1 Tobacco mild green mosaic virus complete genome, strain:Japanese. [29-AUG-2014	98.89	189	100	6356	2	5.947136564	Tobacco mild green mosaic virus:species
Kuala Langat 77	X00255|X00255.1 Gardner-Rasheed feline sarcoma virus (GR-FeSV) proviral genome coding for P70 gag-fgr p	96.83	195	96.92	2025	1	9.333333333	Feline sarcoma virus:no rank
Kuala Langat 77	DQ355023|DQ355023.1 Bell pepper mottle virus, complete genome. [29-AUG-2014]	92.7	322	99.3125	6375	4	20.14117647	Bell pepper mottle virus:species
Kuala Langat 77	FJ959081|FJ959081.1 Circovirus-like genome RW-E, complete genome. [29-AUG-2014]	77.855	171.5	98.755	2782	2	10.891445	Circovirus-like genome RW-E:species
Kuala Langat 77	KJ938717|KJ938717.1 Caribou feces-associated gemycircularvirus, complete genome. [14-SEP-2014]	79.64666667	167.3333333	93.57	2232	3	20.43010753	Caribou feces-associated gemycircularvirus:species
Kuala Langat 77	AY388617|AY388617.1 Bovine adeno-associated virus, complete genome. [29-AUG-2014]	66.8	1040	73.56	4693	1	16.36479864	Bovine adeno-associated virus:no rank
Kuala Langat 77	HM004429|HM004429.1 Micromonas sp. RCC1109 virus MpV1, complete genome. [29-AUG-2014]	79.17	151	95.36	184095	1	0.078220484	Micromonas sp. RCC1109 virus MpV1:species
Kuala Langat 77	KF938901|KF938901.1 UNVERIFIED: Anopheles minimus irodovirus isolate AMIV, complete genome. [29-AUG	63.27	145	99.31	163023	1	0.090171326	Anopheles minimus irodovirus:species
Kuala Langat 77	AF303741|AF303741.1 Chilo iridescent virus complete genome. [29-AUG-2014]	81.58	114	100	212482	1	0.052239719	Invertebrate iridescent virus 6:species
Kuala Langat 77	JF938082|JF938082.1 Bat circovirus ZS/China/2011 isolate YN-BtCV-5, complete genome. [29-AUG-2014]	67.45	259.5	95.01	1818	2	27.22772277	Bat circovirus ZS/China/2011:species
Kuala Langat 77	KC580640|KC580640.1 Canine bocavirus 3 isolate UCD, complete genome. [29-AUG-2014]	74.7	501	99.4	5278	1	9.435392194	Canine bocavirus 3:species
Kuala Langat 77	EF198242|EF198242.1 Marine RNA virus JP-B, complete genome. [29-AUG-2014]	38.31	1004	58.27	8926	1	6.621106879	Marine RNA virus JP-B:species
Kuala Langat 77	JN704610|JN704610.1 Meles meles fecal virus isolate VS4700006, complete genome. [29-AUG-2014]	83.5925	155	96.5475	2198	4	27.02456779	Meles meles fecal virus:species
Kuala Langat 77	V01197|V01197.1 Rous sarcoma virus genome, strain Prague C (Pr-C). [29-AUG-2014]	95.68	316	48.895	9312	2	3.135738832	Rous sarcoma virus:species
Kuala Langat 77	JF755427|JF755427.1 Mouse kobuvirus M-5/USA/2010, complete genome. [29-AUG-2014]	84.415	212	98.86	8198	4	10.53915589	Mouse kobuvirus M-5/USA/2010:species
Kuala Langat 77	AB090161|AB090161.1 Theiler's-like virus of rats NGS910 genomic RNA, complete genome. [29-AUG-2014]	93.61	116	98.385	8021	2	2.842538337	Theiler's-like virus of rats:no rank
Kuala Langat 77	AY563023|AY563023.1 Duck picornavirus TW90A, complete genome. [29-AUG-2014]	69.84	189	100	8289	1	2.280130293	Avian sapelovirus:species
Kuala Langat 77	EF536349|EF536349.1 Ursus maritimus papillomavirus type 1, complete genome. [29-AUG-2014]	96.43	128	65.62	7582	1	1.107887101	Ursus maritimus papillomavirus 1:no rank
Kuala Langat 77	AF174533|AF174533.1 Nodamura virus RNA1 segment, complete sequence. [29-AUG-2014]	66.29333333	497.3333333	99.34666667	3204	3	44.1011236	Nodamura virus:species
Kuala Langat 77	EU839994|EU839994.1 Agrotis ipsilon multiple nucleopolyhedrovirus strain Illinois, complete genome. [29-AU	67.74	115	78.26	155122	1	0.05801885	Agrotis ipsilon multiple nucleopolyhedrovirus:species
Kuala Langat 77	KF170373|KF170373.1 Parvo-like hybrid virus UC1, complete genome. [29-AUG-2014]	100	1075	99.63	3638	1	29.43925234	Parvo-like hybrid virus UC1:no rank
Kuala Langat 77	JQ340310|JQ340310.1 Astrovirus wild boar/WBAstV-1/2011/HUN, complete genome. [29-AUG-2014]	68.83	233	99.14	6707	1	3.444162815	Astrovirus wild boar/WBAstV-1/2011/HUN:species
Kuala Langat 77	AY275539|AY275539.1 Avian encephalomyelitis virus strain L2Z, complete genome. [29-AUG-2014]	68.74333333	230	98.83333333	7059	3	9.647258819	Avian encephalomyelitis virus:species
Kuala Langat 77	HQ425636|HQ425636.1 Avian leukemia virus isolate SCDY1, complete genome. [29-AUG-2014]	100	143	98.6	7489	1	1.882761383	Avian leukemia virus:no rank
Kuala Langat 77	KF214638|KF214638.1 Parvo-like hybrid virus UC11, complete genome. [29-AUG-2014]	100	975	100	3637	1	26.80780863	Parvo-like hybrid virus UC11:no rank
Kuala Langat 77	AB089381|AB089381.1 Paprika mild mottle virus complete genome, strain:Japanese. [29-AUG-2014]	99.116	188.6	98.954	6524	10	28.21888412	Paprika mild mottle virus:species
Kuala Langat 77	KJ859677|KJ859677.1 Penguinpox virus isolate PSan92, complete genome. [29-AUG-2014]	90.74	183	88.52	306862	1	0.05279246	Penguinpox virus:species
Kuala Langat 77	X00956|X00956.1 Black beetle virus (BBV) RNA2 sequence for coat protein precursor [29-AUG-2014]	68.81	246.5	97.94	1399	2	34.73909936	Black beetle virus:species
Kuala Langat 77	KF214645|KF214645.1 Parvo-like hybrid virus UC4, complete genome. [29-AUG-2014]	98.41	1357	97.27	3638	1	36.28367235	Parvo-like hybrid virus UC4:no rank
Kuala Langat 77	AM491609|AM491609.1 Botryotinia fuckeliana partitivirus 1, complete segment 1, genomic RNA [29-AUG-2	93.63	148.5	98.02	1793	2	16.22978249	Botryotinia fuckeliana partitivirus 1:species
Kuala Langat 77	GQ365709|GQ365709.1 Sclerotinia sclerotiorum hypovirulence associated DNA virus 1, complete genome. [2	86.49	112	99.11	2166	1	5.12465374	Sclerotinia sclerotiorum hypovirulence associated DNA virus 1:species
Kuala Langat 77	AF188515|AF188515.1 Cryphonectria hypovirus 3 isolate WY, complete genome. [29-AUG-2014]	71.7	166	95.78	9799	1	1.622614553	Cryphonectria hypovirus 3:species
Kuala Langat 77	FJ528584|FJ528584.1 Solenopsis invicta virus 3 isolate DM, complete genome. [29-AUG-2014]	94	152	98.68	10411	1	1.440783786	Solenopsis invicta virus 3:species
Kuala Langat 77	HE818438|HE818438.1 Tobacco mosaic virus isolate Pingtan-1, complete genome [29-AUG-2014]	96.31333333	1889	99.65666667	6395	3	83.98749023	Tobacco mosaic virus:species
Kuala Langat 77	AY859497|AY859497.1 Pepper mild mottle virus isolate PMMoV-CN, complete genome. [29-AUG-2014]	98.624	252.8	99.436	6356	5	19.82378855	Pepper mild mottle virus:species
Kuala Langat 77	HQ113105|HQ113105.1 Lausannevirus isolate 7715, complete genome. [29-AUG-2014]	94.685	104.5	86.305	346754	2	0.042393166	Lausannevirus:species
Kuala Langat 77	M34077|M34077.1 Tobacco mild green mosaic virus, complete genome. [29-AUG-2014]	100	109	99.01	6355	2	3.398898505	Tobacco mild green mosaic virus:species
Kuala Langat 77	EF428566|EF428566.1 Solenopsis invicta virus 2, complete genome. [29-AUG-2014]	49.406	645.6	91.338	11319	5	23.08507819	Solenopsis invicta virus 2:species
Kuala Langat 77	AB017037|AB017037.1 Himetobi P virus genomic RNA, complete sequence. [29-AUG-2014]	55	1144	26.22	9275	1	3.234501348	Himetobi P virus:species
Kuala Langat 77	U01060|U01060.1 Saccharomyces cerevisiae virus La, complete genome. [29-AUG-2014]	67.31	156	100	4615	1	3.38028169	Saccharomyces cerevisiae virus L-BC (La):species
Kuala Langat 77	AB628188|AB628188.1 Rehmannia mosaic virus genomic RNA, complete genome, isolate: Japanese. [29-AU	98.685	118.5	100	6395	2	3.706020328	Rehmannia mosaic virus:species
Kuala Langat 77	HF920637|HF920637.1 Armadillidium vulgare iridescent virus complete genome [25-JUN-2014]	94.59	114	97.37	220222	1	0.050403684	Armadillidium vulgare iridescent virus:no rank
Kuala Langat 77	DQ890022|DQ890022.1 Paramecium bursaria Chlorella virus FR483, complete genome. [29-AUG-2014]	100	149	98.66	321240	1	0.045760179	Paramecium bursaria Chlorella virus FR483:no rank
Kuala Langat 77	HF920633|HF920633.1 Invertebrate iridovirus 22 complete genome [29-AUG-2014]	94.44	108	100	197693	1	0.054630159	Invertebrate iridovirus 22:species
Kuala Langat 77	D12505|D12505.1 Cucumber green mottle mosaic virus RNA, complete genome. [29-AUG-2014]	98.92153846	190.1538462	98.96384615	6424	13	37.59339975	Cucumber green mottle mosaic virus:species
Kuala Langat 77	DQ365814|DQ365814.1 Avian leukosis virus strain MQNCSU, complete genome. [29-AUG-2014]	99.04	316	98.73	7704	1	4.049844237	Avian leukosis virus:species
Kuala Langat 77	DQ335246|DQ335246.2 Adeno-associated virus-Go.1, complete genome. [29-AUG-2014]	67.595	550.5	77.755	4636	2	19.45642796	Adeno-associated virus-Go.1:no rank
Kuala Langat 77	AF192260|AF192260.1 Periplaneta fuliginosa densovirus, complete genome. [29-AUG-2014]	90.40571429	186.2857143	98.38285714	5454	7	23.54235424	Periplaneta fuliginosa densovirus:no rank
Kuala Langat 77	KF477193|KF477193.1 Tomato mottle mosaic virus isolate MX5, complete genome. [29-AUG-2014]	97.92	146	98.63	6398	1	2.250703345	Tomato mottle mosaic virus:species
Kuala Lumpur 78	JQ898342|JQ898342.1 Kobuvirus sewage Kathmandu isolate KoV-SewKTM, complete genome. [29-AUG-201	94.23214286	345	99.01214286	6939	14	65.75875486	Kobuvirus sewage Kathmandu:no rank
Kuala Lumpur 78	AF332868|AF332868.1 Tomato mosaic virus isolate Queensland, complete genome. [29-AUG-2014]	99.8325	290.25	99.35	6383	4	18.09493968	Tomato mosaic virus:species
Kuala Lumpur 78	AJ417701|AJ417701.1 Tomato mosaic virus camellia strain, complete genome [29-AUG-2014]	100	108	94.44	6383	1	1.597994673	Tomato mosaic virus:species
Kuala Lumpur 78	EF101928|EF101928.1 Acanthocystis turfacea Chlorella virus 1, complete genome. [29-AUG-2014]	77.46666667	150.3333333	98.42666667	288047	3	0.154141512	Acanthocystis turfacea Chlorella virus 1:species
Kuala Lumpur 78	AJ243571|AJ243571.1 Tomato mosaic virus (K1 strain) complete RNA genome [29-AUG-2014]	100	210.5	98.79	6383	2	6.532978223	Tomato mosaic virus:species
Kuala Lumpur 78	JX962719|JX962719.1 Acanthamoeba polyphaga moumouvirus, complete genome. [29-AUG-2014]	70	138	86.96	1021348	1	0.010280531	Acanthamoeba polyphaga moumouvirus:no rank
Kuala Lumpur 78	GQ918152|GQ918152.1 Wiseana iridescent virus, complete genome. [29-AUG-2014]	87.5	137	96.435	205791	2	0.128285494	Wiseana iridescent virus:no rank
Kuala Lumpur 78	AB078435|AB078435.1 Tobacco mild green mosaic virus complete genome, strain:Japanese. [29-AUG-2014	98.306	488.4	99.522	6356	5	38.27879169	Tobacco mild green mosaic virus:species
Kuala Lumpur 78	DQ355023|DQ355023.1 Bell pepper mottle virus, complete genome. [29-AUG-2014]	83.89	158.5	98.41	6375	2	4.894117647	Bell pepper mottle virus:species
Kuala Lumpur 78	X13744|X13744.1 Avian Retrovirus IC10 proviral genome [29-AUG-2014]	97.78	137	98.54	4213	1	3.204367434	Avian retrovirus IC10:species
Kuala Lumpur 78	GU244497|GU244497.1 Cafeteria roenbergensis virus BV-PW1, complete genome. [29-AUG-2014]	85.065	122	89.755	617453	2	0.03498242	Cafeteria roenbergensis virus BV-PW1:no rank
Kuala Lumpur 78	HM004429|HM004429.1 Micromonas sp. RCC1109 virus MpV1, complete genome. [29-AUG-2014]	86.27	159	96.23	184095	1	0.083109264	Micromonas sp. RCC1109 virus MpV1:species
Kuala Lumpur 78	FR878069|FR878069.1 Tobacco mosaic virus strain Ohio V, complete genome, genomic RNA [29-AUG-2014	98.53	285	98.765	6392	2	8.823529412	Tobacco mosaic virus strain Ohio V:no rank
Kuala Lumpur 78	AF022937|AF022937.1 Rhopalosiphum padi virus complete genome. [29-AUG-2014]	93.325	134	97.22	10011	2	2.607132155	Rhopalosiphum padi virus:species
Kuala Lumpur 78	V01197|V01197.1 Rous sarcoma virus genome, strain Prague C (Pr-C). [29-AUG-2014]	93.44333333	180	97.91166667	9312	6	11.37242268	Rous sarcoma virus:species
Kuala Lumpur 78	JF755427|JF755427.1 Mouse kobuvirus M-5/USA/2010, complete genome. [29-AUG-2014]	84.472	292.8	86.814	8198	5	14.63771652	Mouse kobuvirus M-5/USA/2010:species
Kuala Lumpur 78	AM398436|AM398436.1 Brugmansia sp. tobamovirus complete virion genome [29-AUG-2014]	84.62	119	98.32	6381	1	1.833568406	Brugmansia mild mottle virus:species
Kuala Lumpur 78	KC991097|KC991097.1 Solenopsis invicta densovirus isolate SiDNV-Arg, complete genome. [29-AUG-2014]	64.625	3960	48.41	5280	2	71.00378788	Solenopsis invicta densovirus:species
Kuala Lumpur 78	M37980|M37980.1 Avian leukosis virus - RSA, complete genome. [29-AUG-2014]	98.89	270	100	7286	1	3.70573703	Avian leukosis virus - RSA:no rank
Kuala Lumpur 78	M20301|M20301.1 Theiler murine encephalomyelitis virus, complete genome. [29-AUG-2014]	93.75	100	96	8093	1	1.186210305	Theiler's encephalomyelitis virus:no rank
Kuala Lumpur 78	DQ333351|DQ333351.1 Choristoneura occidentalis granulovirus, complete genome. [29-AUG-2014]	92.31	405	19.26	104710	1	0.074491453	Choristoneura occidentalis granulovirus:species
Kuala Lumpur 78	HQ425636|HQ425636.1 Avian leukemia virus isolate SCDY1, complete genome. [29-AUG-2014]	97.67	258	100	7489	1	3.445052744	Avian leukemia virus:no rank
Kuala Lumpur 78	GQ404855|GQ404855.1 Cyclovirus NG14, complete genome. [29-AUG-2014]	77.14	110	95.45	1795	1	5.849582173	Cyclovirus NG14:species
Kuala Lumpur 78	JX185430|JX185430.1 Dragonfly-associated circular virus 1 isolate FL1-2X-2010, complete genome. [29-AU	60.94	651	58.99	2225	1	16.85393258	Dragonfly-associated circular virus 1:species
Kuala Lumpur 78	AB261167|AB261167.1 Cucumber mottle virus genomic RNA, complete genome. [29-AUG-2014]	92.055	116	98.265	6485	2	3.515805705	Cucumber mottle virus:species
Kuala Lumpur 78	KF214638|KF214638.1 Parvo-like hybrid virus UC11, complete genome. [29-AUG-2014]	100	561	99.47	3637	1	15.34231509	Parvo-like hybrid virus UC11:no rank
Kuala Lumpur 78	AB089381|AB089381.1 Paprika mild mottle virus complete genome, strain:Japanese. [29-AUG-2014]	100	128	98.44	6524	1	1.931330472	Paprika mild mottle virus:species
Kuala Lumpur 78	AY037928|AY037928.1 Human endogenous retrovirus K113 complete genome. [29-AUG-2014]	90.615	128.5	98.065	9472	2	2.660472973	Human endogenous retrovirus K113:no rank
Kuala Lumpur 78	JQ941880|JQ941880.1 Bovine hungarovirus 1 strain BHUV1/2008/HUN, complete genome. [29-AUG-2014]	96.685	134	98.445	7583	2	3.481471713	Bovine hungarovirus 1:no rank
Kuala Lumpur 78	KF214645|KF214645.1 Parvo-like hybrid virus UC4, complete genome. [29-AUG-2014]	100	1080	100	3638	1	29.68664101	Parvo-like hybrid virus UC4:no rank
Kuala Lumpur 78	KF738874|KF738874.1 Dragonfly larvae associated circular virus-2 isolate DflaCV-2 NZ-PG8-LS, complete ge	77.855	114	94.77	2398	2	8.632193495	Dragonfly larvae associated circular virus-2:species
Kuala Lumpur 78	JN391187|JN391187.1 Tianjin totivirus, complete genome. [29-AUG-2014]	50	306	99.02	7611	1	3.902246748	Tianjin totivirus:species
Kuala Lumpur 78	GQ365709|GQ365709.1 Sclerotinia sclerotiorum hypovirulence associated DNA virus 1, complete genome. [2	76.32	119	95.8	2166	1	4.986149584	Sclerotinia sclerotiorum hypovirulence associated DNA virus 1:species
Kuala Lumpur 78	HE818438|HE818438.1 Tobacco mosaic virus isolate Pingtan-1, complete genome [29-AUG-2014]	96.50666667	1945.333333	99.58	6395	3	78.68647381	Tobacco mosaic virus:species
Kuala Lumpur 78	AY859497|AY859497.1 Pepper mild mottle virus isolate PMMoV-CN, complete genome. [29-AUG-2014]	99.1	146.6666667	97.24666667	6356	3	6.088735053	Pepper mild mottle virus:species
Kuala Lumpur 78	EU780426|EU780426.1 Spodoptera litura nucleopolyhedrovirus II, complete genome. [29-AUG-2014]	83.33	296	54.73	148634	1	0.108992559	Spodoptera litura nucleopolyhedrovirus II:species
Kuala Lumpur 78	M34077|M34077.1 Tobacco mild green mosaic virus, complete genome. [29-AUG-2014]	98.835	268.5	99.11	6355	2	8.402832415	Tobacco mild green mosaic virus:species
Kuala Lumpur 78	JF411744|JF411744.1 Paramecium bursaria Chlorella virus 1 (PBCV-1), complete genome. [29-AUG-2014]	71.01	228	90.79	330611	1	0.062611347	Paramecium bursaria Chlorella virus 1:species
Kuala Lumpur 78	AB628188|AB628188.1 Rehmannia mosaic virus genomic RNA, complete genome, isolate: Japanese. [29-AU	98.23666667	522	99.37666667	6395	9	70.39874902	Rehmannia mosaic virus:species
Kuala Lumpur 78	HF920637|HF920637.1 Armadillidium vulgare iridescent virus complete genome [25-JUN-2014]	73.33	139	97.12	220222	1	0.061301777	Armadillidium vulgare iridescent virus:no rank
Kuala Lumpur 78	HF920633|HF920633.1 Invertebrate iridovirus 22 complete genome [29-AUG-2014]	80.74	132	93.115	197693	2	0.124435362	Invertebrate iridovirus 22:species
Kuala Lumpur 78	D12505|D12505.1 Cucumber green mottle mosaic virus RNA, complete genome. [29-AUG-2014]	98.71625	279.5	98.93125	6424	8	34.04420922	Cucumber green mottle mosaic virus:species
Kuala Lumpur 78	DQ174103|DQ174103.1 Atlantic salmon swim bladder sarcoma virus from USA, complete genome. [29-AUG-	95	126	95.24	10305	1	1.164483261	Atlantic salmon swim bladder sarcoma virus:species
Kuala Lumpur 78	EU715328|EU715328.1 Armigeres subalbatus virus SaX06-AK20, complete genome. [29-AUG-2014]	73.21	170	98.82	7510	1	2.23701731	Armigeres subalbatus virus SaX06-AK20:species
Kuala Lumpur 78	EF165067|EF165067.2 Saffold virus, complete genome. [29-AUG-2014]	97.14	108	97.22	8115	1	1.293900185	Saffold virus:no rank

Supplementary Table S1 B: Virus-like contig found with DNA sequencing

location	Closest ressemblance	Per cent identity	query length	per cent covera	Length of	Nur	Per cent of cove	Lowest common ancestor
Amager East 46	KJ547633|KJ547633.1 Sewage-associated circular DNA virus-9 isolate SaCV-9 NZ-BS3681-2012, complete ;	97.73	133	99.25	2423	1	5.447791993	Sewage-associated circular DNA virus-9:species
Amager East 46	JX885207|JX885207.1 Megavirus lba isolate LBA111, complete genome. [29-AUG-2014]	92.59	194	41.75	1230522	1	0.006582572	Megavirus lba:species
Amager East 47	U01834|U01834.1 Multimammate rat papillomavirus, complete genome. [29-AUG-2014]	82.05	120	97.5	7687	1	1.522050215	Iotapapillomavirus 1:species
Amager East 47	JF755402|JF755402.1 Rodent stool-associated circular genome virus strain RodSCV M-89, complete genome	67.15	422	97.39	2069	1	19.86466892	Rodent stool-associated circular genome virus:species

Amager East 47	DQ890022lDQ890022.1 Paramecium bursaria Chlorella virus FR483, complete genome. [29-AUG-2014]	94.29	107	98.13	321240	1	0.032685842	Paramecium bursaria Chlorella virus FR483:no rank
Amager East 48	EF101928lEF101928.1 Acanthocystis turfacea Chlorella virus 1, complete genome. [29-AUG-2014]	70.45	144	91.67	288047	1	0.045825855	Acanthocystis turfacea Chlorella virus 1:species
Amager East 48	DQ890022lDQ890022.1 Paramecium bursaria Chlorella virus FR483, complete genome. [29-AUG-2014]	95.12	126	97.62	321240	1	0.03828913	Paramecium bursaria Chlorella virus FR483:no rank
Amager East 49	EF101928lEF101928.1 Acanthocystis turfacea Chlorella virus 1, complete genome. [29-AUG-2014]	81.48	164	98.78	288047	1	0.056240822	Acanthocystis turfacea Chlorella virus 1:species
Amager East 49	GQ918152lGQ918152.1 Wiseana iridescent virus, complete genome. [29-AUG-2014]	85.71	108	97.22	205791	1	0.051022639	Wiseana iridescent virus:no rank
Amager East 49	U60315lU60315.1 Molluscum contagiosum virus subtype 1, complete genome. [29-AUG-2014]	77.87	1307	25.48	190289	1	0.190762472	Molluscum contagiosum virus subtype 1:no rank
Amager East 49	GQ180114lGQ180114.1 Rattus norvegicus papillomavirus 1 EES-2009, complete genome. [29-AUG-2014]	100	140	98.57	7378	1	1.87042559	Rattus norvegicus papillomavirus 1 EES-2009:no rank
Amager East 49	JF755402lJF755402.1 Rodent stool-associated circular genome virus strain RodSCV M-89, complete genome	69.53	387	99.22	2069	1	18.55969067	Rodent stool-associated circular genome virus:species
Amager East 49	DQ890022lDQ890022.1 Paramecium bursaria Chlorella virus FR483, complete genome. [29-AUG-2014]	100	101.5	99.015	321240	2	0.062570041	Paramecium bursaria Chlorella virus FR483:no rank
Amager East 51	KF703446lKF703446.1 Retroperitoneal fibromatosis-associated herpesvirus isolate RFHVMnM78114, comple	64.1	118	96.61	127320	1	0.091894439	Retroperitoneal fibromatosis-associated herpesvirus:no rank
Amager East 51	AY037928lAY037928.1 Human endogenous retrovirus K113 complete genome. [29-AUG-2014]	91.95142857	146.2857143	93.86571429	9472	7	10.13513514	Human endogenous retrovirus K113:no rank
Amager East 51	DQ890022lDQ890022.1 Paramecium bursaria Chlorella virus FR483, complete genome. [29-AUG-2014]	98.485	103.5	98.565	321240	2	0.063503922	Paramecium bursaria Chlorella virus FR483:no rank
Amager East 52	HM004432lHM004432.1 Bathycoccus sp. RCC1105 virus BpV1, complete genome. [29-AUG-2014]	55.26	219	100	198519	1	0.111828087	Bathycoccus sp. RCC1105 virus BpV1:no rank
Amager East 52	HM049560lHM049560.1 Murine adenovirus 2 isolate K87, complete genome. [29-AUG-2014]	91.89	112	99.11	35203	1	0.315314036	Murine adenovirus 2:no rank
Amager East 52	EF101928lEF101928.1 Acanthocystis turfacea Chlorella virus 1, complete genome. [29-AUG-2014]	74.12	127.25	96.35	288047	4	0.170805459	Acanthocystis turfacea Chlorella virus 1:species
Amager East 52	JX962719lJX962719.1 Acanthamoeba polyphaga moumouvirus, complete genome. [29-AUG-2014]	43.62	422	98.82	1021348	1	0.042003313	Acanthamoeba polyphaga moumouvirus:no rank
Amager East 52	HM004429lHM004429.1 Micromonas sp. RCC1109 virus MpV1, complete genome. [29-AUG-2014]	75	121	99.17	184095	1	0.065183737	Micromonas sp. RCC1109 virus MpV1:species
Amager East 52	JN704610lJN704610.1 Meles meles fecal virus isolate VS4700006, complete genome. [29-AUG-2014]	67.36	432	97.92	2198	1	19.65423112	Meles meles fecal virus:species
Amager East 52	JQ037754lJQ037754.1 Artibeus jamaicensis parvovirus 1, complete genome. [29-AUG-2014]	66.67	255	95.29	4595	1	5.28835691	Artibeus jamaicensis parvovirus 1:species
Amager East 52	X02449lX02449.1 Hamster polyomavirus (HapV) genome [29-AUG-2014]	86.98	169.5	99.055	5366	2	6.26164741	Hamster polyomavirus:species
Amager East 52	U01834lU01834.1 Multimammate rat papillomavirus, complete genome. [29-AUG-2014]	64.58	148	97.3	7687	1	1.873292572	Iotapapillomavirus 1:species
Amager East 52	DQ890022lDQ890022.1 Paramecium bursaria Chlorella virus FR483, complete genome. [29-AUG-2014]	98.66	112	99.765	321240	4	0.1391483	Paramecium bursaria Chlorella virus FR483:no rank
Amager East 52	JF755418lJF755418.1 Peromyscus papillomavirus type 1 strain M-14 PmPV1, complete genome. [29-AUG-2	75.76	102	97.06	7704	1	1.285046729	Peromyscus papillomavirus type 1:species
Amager East 53	EF101928lEF101928.1 Acanthocystis turfacea Chlorella virus 1, complete genome. [29-AUG-2014]	76.6	149	94.63	288047	1	0.048950345	Acanthocystis turfacea Chlorella virus 1:species
Amager East 53	KJ577819lKJ577819.1 Porcine stool-associated circular virus 6 isolate XP1, complete genome. [02-SEP-201	75.23	330	99.09	2603	1	12.56242797	Porcine stool-associated circular virus 6:species
Amager East 53	HM004429lHM004429.1 Micromonas sp. RCC1109 virus MpV1, complete genome. [29-AUG-2014]	80.95	126	100	184095	1	0.068442923	Micromonas sp. RCC1109 virus MpV1:species
Amager East 53	X02449lX02449.1 Hamster polyomavirus (HapV) genome [29-AUG-2014]	97.22	109	99.08	5366	1	2.012672382	Hamster polyomavirus:species
Amager East 53	JF755402lJF755402.1 Rodent stool-associated circular genome virus strain RodSCV M-89, complete genome	78.18	168	98.21	2069	1	7.974867086	Rodent stool-associated circular genome virus:species
Amager East 53	FJ940765lFJ940765.3 Anguillid herpesvirus 1 strain 500138, complete genome. [29-AUG-2014]	68.57	106	99.06	248526	1	0.042249101	Anguillid herpesvirus 1:species
Amager East 53	KF740664lKF740664.1 Pithovirus sibericum isolate P1084-T, complete genome. [29-AUG-2014]	69.57	147	93.88	610033	1	0.020162844	Pithovirus sibericum:species
Amager East 53	DQ890022lDQ890022.1 Paramecium bursaria Chlorella virus FR483, complete genome. [29-AUG-2014]	88.5475	122.5	98.585	321240	4	0.151288756	Paramecium bursaria Chlorella virus FR483:no rank
Amager East 54	HM049560lHM049560.1 Murine adenovirus 2 isolate K87, complete genome. [29-AUG-2014]	94.29	109	96.33	35203	1	0.298270034	Murine adenovirus 2:no rank
Amager East 54	KJ577819lKJ577819.1 Porcine stool-associated circular virus 6 isolate XP1, complete genome. [02-SEP-201	68.52	178	91.01	2603	1	6.223588167	Porcine stool-associated circular virus 6:species
Amager East 54	U01834lU01834.1 Multimammate rat papillomavirus, complete genome. [29-AUG-2014]	83.69	143.3333333	99.07	7687	3	5.50279693	Iotapapillomavirus 1:species
Amager East 54	GQ180114lGQ180114.1 Rattus norvegicus papillomavirus 1 EES-2009, complete genome. [29-AUG-2014]	100	137	98.54	7378	1	1.829764164	Rattus norvegicus papillomavirus 1 EES-2009:no rank
Amager East 54	JF755402lJF755402.1 Rodent stool-associated circular genome virus strain RodSCV M-89, complete genome	71.72	300	99	2069	1	14.35476075	Rodent stool-associated circular genome virus:species
Amager East 54	KF740664lKF740664.1 Pithovirus sibericum isolate P1084-T, complete genome. [29-AUG-2014]	75	122	81.15	610033	1	0.01770396	Pithovirus sibericum:species
Amager East 54	JF411744lJF411744.1 Paramecium bursaria Chlorella virus 1 (PBCV-1), complete genome. [29-AUG-2014]	82.86	108	97.22	330611	1	0.031759379	Paramecium bursaria Chlorella virus 1:species
Amager East 54	DQ890022lDQ890022.1 Paramecium bursaria Chlorella virus FR483, complete genome. [29-AUG-2014]	90.995	132.5	99.64	321240	2	0.082181547	Paramecium bursaria Chlorella virus FR483:no rank
Amager East 55	EF101928lEF101928.1 Acanthocystis turfacea Chlorella virus 1, complete genome. [29-AUG-2014]	80	143	94.41	288047	1	0.046867352	Acanthocystis turfacea Chlorella virus 1:species
Amager East 55	KJ577819lKJ577819.1 Porcine stool-associated circular virus 6 isolate XP1, complete genome. [02-SEP-201	75.27	347	75.715	2603	2	17.40299654	Porcine stool-associated circular virus 6:species
Amager East 55	U01834lU01834.1 Multimammate rat papillomavirus, complete genome. [29-AUG-2014]	88	151	99.34	7687	1	1.951346429	Iotapapillomavirus 1:species
Amager East 55	GQ351275lGQ351275.1 Chimpanzee stool associated circular ssDNA virus isolate GM510, complete genome	70.27	142	78.17	2589	1	4.287369641	Chimpanzee stool associated circular ssDNA virus:species
Amager East 55	JF755402lJF755402.1 Rodent stool-associated circular genome virus strain RodSCV M-89, complete genome	71.96	324	99.07	2069	1	15.51474142	Rodent stool-associated circular genome virus:species
Amager East 55	KJ547633lKJ547633.1 Sewage-associated circular DNA virus-9 isolate SaCV-9 NZ-BS3681-2012, complete	90	121	99.17	2423	1	4.952538176	Sewage-associated circular DNA virus-9:species
Amager East 55	DQ890022lDQ890022.1 Paramecium bursaria Chlorella virus FR483, complete genome. [29-AUG-2014]	97.5	114.5	99.54	321240	2	0.070974972	Paramecium bursaria Chlorella virus FR483:no rank
Egedal 21	X64346lX64346.1 Saimiriine herpesvirus 2 complete genome [29-AUG-2014]	76.09	139	99.28	112930	1	0.122199593	Saimiriine herpesvirus 2:species
Egedal 21	EF101928lEF101928.1 Acanthocystis turfacea Chlorella virus 1, complete genome. [29-AUG-2014]	74.52	114.5	83.49	288047	2	0.067697286	Acanthocystis turfacea Chlorella virus 1:species
Egedal 21	KF246569lKF246569.1 Fur seal faeces associated circular DNA virus isolate as50, complete genome. [29-AU	44.98	1926	31.62	2925	1	20.61538462	Fur seal faeces associated circular DNA virus:species
Egedal 21	KJ938717lKJ938717.1 Caribou feces-associated gemycircularvirus, complete genome. [14-SEP-2014]	81.33	1361	16.53	2232	1	10.08064516	Caribou feces-associated gemycircularvirus:species
Egedal 21	KJ577819lKJ577819.1 Porcine stool-associated circular virus 6 isolate XP1, complete genome. [02-SEP-201	78.72	283	99.65	2603	1	10.83365348	Porcine stool-associated circular virus 6:species
Egedal 21	AB611707lAB611707.1 Simian retrovirus Y proviral DNA, complete genome, strain: Y001. [29-AUG-2014]	76.19	128	98.44	8145	1	1.546961326	Simian retrovirus Y:species
Egedal 21	JF755402lJF755402.1 Rodent stool-associated circular genome virus strain RodSCV M-89, complete genome	63.04	353	78.19	2069	1	13.33977767	Rodent stool-associated circular genome virus:species
Egedal 21	KJ547633lKJ547633.1 Sewage-associated circular DNA virus-9 isolate SaCV-9 NZ-BS3681-2012, complete	90.42333333	268.3333333	92.37333333	2423	3	25.13413124	Sewage-associated circular DNA virus-9:species
Egedal 21	DQ890022lDQ890022.1 Paramecium bursaria Chlorella virus FR483, complete genome. [29-AUG-2014]	96.71	153.6666667	98.97666667	321240	3	0.141949944	Paramecium bursaria Chlorella virus FR483:no rank
Egedal 23	AF204951lAF204951.2 Ectocarpus siliculosus virus, complete genome. [29-AUG-2014]	92.31	107	72.9	335593	1	0.02324244	Ectocarpus siliculosus virus 1:species
Egedal 23	EF101928lEF101928.1 Acanthocystis turfacea Chlorella virus 1, complete genome. [29-AUG-2014]	90.91	100	99	288047	1	0.034369391	Acanthocystis turfacea Chlorella virus 1:species
Egedal 23	JX185430lJX185430.1 Dragonfly-associated circular virus 1 isolate FL1-2X-2010, complete genome. [29-AU	90.91	103	96.12	2225	1	4.449438202	Dragonfly-associated circular virus 1:species
Egedal 23	HQ113105lHQ113105.1 Lausannevirus isolate 7715, complete genome. [29-AUG-2014]	88	167	89.82	346754	1	0.043258333	Lausannevirus:species
Egedal 23	JF755402lJF755402.1 Rodent stool-associated circular genome virus strain RodSCV M-89, complete genome	71.59	266	99.25	2069	1	12.75978734	Rodent stool-associated circular genome virus:species
Egedal 23	KJ547633lKJ547633.1 Sewage-associated circular DNA virus-9 isolate SaCV-9 NZ-BS3681-2012, complete	93.64	2573	40.34	2423	1	42.83945522	Sewage-associated circular DNA virus-9:species
Egedal 23	DQ890022lDQ890022.1 Paramecium bursaria Chlorella virus FR483, complete genome. [29-AUG-2014]	100	120	97.5	321240	1	0.036421367	Paramecium bursaria Chlorella virus FR483:no rank
Egedal 24	EF101928lEF101928.1 Acanthocystis turfacea Chlorella virus 1, complete genome. [29-AUG-2014]	63.83	144	97.92	288047	1	0.048950345	Acanthocystis turfacea Chlorella virus 1:species
Egedal 24	KF938901lKF938901.1 UNVERIFIED: Anopheles minimus irodovirus isolate AMIV, complete genome. [29-AUG	97.37	118	96.61	163023	1	0.069928783	Anopheles minimus irodovirus:species
Egedal 24	EU410304lEU410304.1 Vaccinia virus GLV-1h68, complete genome. [29-AUG-2014]	60	168	98.21	203057	1	0.081257972	Vaccinia virus GLV-1h68:no rank
Egedal 24	DQ890022lDQ890022.1 Paramecium bursaria Chlorella virus FR483, complete genome. [29-AUG-2014]	94.0775	135.75	98.7525	321240	4	0.167164737	Paramecium bursaria Chlorella virus FR483:no rank
Botanical Garden of Copenhagen 60	KC977570lKC977570.1 Pandoravirus dulcis, complete genome. [29-AUG-2014]	91.18	106	96.23	1908524	1	0.005344444	Pandoravirus dulcis:species
Botanical Garden of Copenhagen 60	HM049560lHM049560.1 Murine adenovirus 2 isolate K87, complete genome. [29-AUG-2014]	84.78666667	122.6666667	97.83666667	35203	3	1.022640116	Murine adenovirus 2:no rank
Botanical Garden of Copenhagen 60	EF101928lEF101928.1 Acanthocystis turfacea Chlorella virus 1, complete genome. [29-AUG-2014]	76.12666667	133	95.44	288047	3	0.132270081	Acanthocystis turfacea Chlorella virus 1:species
Botanical Garden of Copenhagen 60	DQ643392lDQ643392.1 Aedes taeniorhynchus iridescent virus, complete genome. [29-AUG-2014]	82.61	142	97.18	191100	1	0.072213501	Aedes taeniorhynchus iridescent virus:no rank
Botanical Garden of Copenhagen 60	JN704610lJN704610.1 Meles meles fecal virus isolate VS4700006, complete genome. [29-AUG-2014]	64.34	589	94.8	2198	2	52.82074613	Meles meles fecal virus:species
Botanical Garden of Copenhagen 60	X02449lX02449.1 Hamster polyomavirus (HapV) genome [29-AUG-2014]	73.5375	316.5	83.5625	5366	4	19.56764816	Hamster polyomavirus:species
Botanical Garden of Copenhagen 60	U01834lU01834.1 Multimammate rat papillomavirus, complete genome. [29-AUG-2014]	75.575	241.75	92.555	7687	4	11.00559386	Iotapapillomavirus 1:species
Botanical Garden of Copenhagen 60	KC771281lKC771281.1 Human cyclovirus VS5700009, complete genome. [29-AUG-2014]	64.2	247	98.38	1832	1	11.95414847	Human cyclovirus VS5700009:species
Botanical Garden of Copenhagen 60	EU835513lEU835513.1 Murine adenovirus 3, complete genome. [29-AUG-2014]	92.5	164	73.17	30570	1	0.392541708	Murine adenovirus 3:no rank
Botanical Garden of Copenhagen 60	JF411744lJF411744.1 Paramecium bursaria Chlorella virus 1 (PBCV-1), complete genome. [29-AUG-2014]	76.125	148	99.325	330611	2	0.081666974	Paramecium bursaria Chlorella virus 1:species
Botanical Garden of Copenhagen 60	DQ890022lDQ890022.1 Paramecium bursaria Chlorella virus FR483, complete genome. [29-AUG-2014]	93.97666667	135.3333333	99.11666667	321240	3	0.125140082	Paramecium bursaria Chlorella virus FR483:no rank
Botanical Garden of Copenhagen 60	JF755418lJF755418.1 Peromyscus papillomavirus type 1 strain M-14 PmPV1, complete genome. [29-AUG-2	68.18	136	97.06	7704	1	1.713395639	Peromyscus papillomavirus type 1:species
Botanical Garden of Copenhagen 60	DQ335246lDQ335246.2 Adeno-associated virus-Go.1, complete genome. [29-AUG-2014]	61.34	4187	53.67	4636	1	48.27437446	Adeno-associated virus-Go.1:no rank
Botanical Garden of Copenhagen 61	HM049560lHM049560.1 Murine adenovirus 2 isolate K87, complete genome. [29-AUG-2014]	81.82	136	96.875	35203	2	0.749936085	Murine adenovirus 2:no rank
Botanical Garden of Copenhagen 61	EF101928lEF101928.1 Acanthocystis turfacea Chlorella virus 1, complete genome. [29-AUG-2014]	70.59	155	98.71	288047	1	0.053116332	Acanthocystis turfacea Chlorella virus 1:species
Botanical Garden of Copenhagen 61	JX645345lJX645345.1 Rat minute virus isolate NTU2, complete genome. [29-AUG-2014]	97.3	113	98.23	4780	1	2.322175732	Rat minute virus:species
Botanical Garden of Copenhagen 61	D10032lD10032.1 Baboon endogenous virus strain M7 proviral DNA, complete genome. [29-AUG-2014]	84	165	90.91	8507	1	1.763253791	Baboon endogenous virus strain M7:no rank
Botanical Garden of Copenhagen 61	GQ918152lGQ918152.1 Wiseana iridescent virus, complete genome. [29-AUG-2014]	85.225	128.5	88.335	205791	2	0.110792017	Wiseana iridescent virus:no rank
Botanical Garden of Copenhagen 61	KC580640lKC580640.1 Canine bocavirus 3 isolate UCD, complete genome. [29-AUG-2014]	54.29	2658	54.18	5278	1	27.51042061	Canine bocavirus 3:species
Botanical Garden of Copenhagen 61	GU188428lGU188428.1 Fowl adenovirus C isolate ON1, complete genome. [29-AUG-2014]	78.57	394	53.3	45667	1	0.453281363	Fowl aviadenovirus C:species
Botanical Garden of Copenhagen 61	DQ280312lDQ280312.2 Gallus gallus MLV-related endogenous retrovirus, complete genome. [29-AUG-2014	64.29	128	98.44	9133	1	1.34676448	Gallus gallus:species
Botanical Garden of Copenhagen 61	X02449lX02449.1 Hamster polyomavirus (HapV) genome [29-AUG-2014]	90.215	165.5	96.74	5366	2	5.982109579	Hamster polyomavirus:species
Botanical Garden of Copenhagen 61	U01834lU01834.1 Multimammate rat papillomavirus, complete genome. [29-AUG-2014]	55.38666667	8310.666667	18.42333333	7687	3	59.82828151	Iotapapillomavirus 1:species
Botanical Garden of Copenhagen 61	AY037928lAY037928.1 Human endogenous retrovirus K113 complete genome. [29-AUG-2014]	90.91	167	98.8	9472	1	1.741976351	Human endogenous retrovirus K113:no rank
Botanical Garden of Copenhagen 61	JF411744lJF411744.1 Paramecium bursaria Chlorella virus 1 (PBCV-1), complete genome. [29-AUG-2014]	78.79	102	97.06	330611	1	0.029944557	Paramecium bursaria Chlorella virus 1:species
Botanical Garden of Copenhagen 61	DQ890022lDQ890022.1 Paramecium bursaria Chlorella virus FR483, complete genome. [29-AUG-2014]	95.69777778	130.1111111	95.16333333	321240	9	0.345536048	Paramecium bursaria Chlorella virus FR483:no rank
Botanical Garden of Copenhagen 61	JX867617lJX867617.1 Murid herpesvirus 8 isolate England, complete genome. [29-AUG-2014]	100	132	100	202946	1	0.065041932	Murid herpesvirus 8:species
Botanical Garden of Copenhagen 61	DQ335246lDQ335246.2 Adeno-associated virus-Go.1, complete genome. [29-AUG-2014]	61.34	4298	52.28	4636	1	48.27437446	Adeno-associated virus-Go.1:no rank
Hong Kong 71	KJ577819lKJ577819.1 Porcine stool-associated circular virus 6 isolate XP1, complete genome. [02-SEP-201	77.24	373	98.93	2603	1	14.17595083	Porcine stool-associated circular virus 6:species
Hong Kong 73	GQ918152lGQ918152.1 Wiseana iridescent virus, complete genome. [29-AUG-2014]	91.18	106	96.23	205791	1	0.04956485	Wiseana iridescent virus:no rank
Hong Kong 73	HM004429lHM004429.1 Micromonas sp. RCC1109 virus MpV1, complete genome. [29-AUG-2014]	67.5	121	99.17	184095	1	0.065183737	Micromonas sp. RCC1109 virus MpV1:species
Hong Kong 73	DQ890022lDQ890022.1 Paramecium bursaria Chlorella virus FR483, complete genome. [29-AUG-2014]	92.65333333	128.6666667	98.71666667	321240	3	0.118602914	Paramecium bursaria Chlorella virus FR483:no rank
Malaysia 69	HM049560lHM049560.1 Murine adenovirus 2 isolate K87, complete genome. [29-AUG-2014]	87.18	119	98.32	35203	1	0.332358038	Murine adenovirus 2:no rank
Malaysia 69	EF101928lEF101928.1 Acanthocystis turfacea Chlorella virus 1, complete genome. [29-AUG-2014]	61.17	632	97.78	288047	1	0.21454832	Acanthocystis turfacea Chlorella virus 1:species
Malaysia 69	GU244497lGU244497.1 Cafeteria roenbergensis virus BV-PW1, complete genome. [29-AUG-2014]	44.97	664	66.87	617453	1	0.051016029	Cafeteria roenbergensis virus BV-PW1:no rank
Malaysia 69	AY388617lAY388617.1 Bovine adeno-associated virus, complete genome. [29-AUG-2014]	80	211	99.53	4693	1	4.474749627	Bovine adeno-associated virus:no rank
Malaysia 69	JN704610lJN704610.1 Meles meles fecal virus isolate VS4700006, complete genome. [29-AUG-2014]	71.15	483	96.89	2198	1	21.29208371	Meles meles fecal virus:species
Malaysia 69	JQ037754lJQ037754.1 Artibeus jamaicensis parvovirus 1, complete genome. [29-AUG-2014]	69.64	192	87.5	4595	1	3.656147987	Artibeus jamaicensis parvovirus 1:species
Malaysia 69	DQ335246lDQ335246.2 Adeno-associated virus-Go.1, complete genome. [29-AUG-2014]	82.35	205	99.51	4636	1	4.400345125	Adeno-associated virus-Go.1:no rank
Malaysia 69	GU225708lGU225708.1 Human papillomavirus type 128, complete genome. [29-AUG-2014]	100	138	100	7259	1	1.901088304	Human papillomavirus type 128:no rank
Malaysia 70	KJ577819lKJ577819.1 Porcine stool-associated circular virus 6 isolate XP1, complete genome. [02-SEP-201	75.45	510	98.24	2603	1	19.24702267	Porcine stool-associated circular virus 6:species
Malaysia 70	JF755402lJF755402.1 Rodent stool-associated circular genome virus strain RodSCV M-89, complete genome	71.72	300	99	2069	1	14.35476075	Rodent stool-associated circular genome virus:species
Malaysia 70	KJ547633lKJ547633.1 Sewage-associated circular DNA virus-9 isolate SaCV-9 NZ-BS3681-2012, complete	91.43	109	96.33	2423	1	4.333470904	Sewage-associated circular DNA virus-9:species
Faculty of Health and Medical Sciences 81	JQ303225lJQ303225.1 Rhinolophus ferrumequinum retrovirus isolate RfRV, complete genome. [02-JUL-201	79.17	144	100	8389	1	1.716533556	Rhinolophus ferrumequinum retrovirus:no rank
Faculty of Health and Medical Sciences 81	EF101928lEF101928.1 Acanthocystis turfacea Chlorella virus 1, complete genome. [29-AUG-2014]	85.71	113	92.92	288047	1	0.036452385	Acanthocystis turfacea Chlorella virus 1:species
Faculty of Health and Medical Sciences 81	D10032lD10032.1 Baboon endogenous virus strain M7 proviral DNA, complete genome. [29-AUG-2014]	77.5	120	100	8507	1	1.410603033	Baboon endogenous virus strain M7:no rank
Faculty of Health and Medical Sciences 81	GQ918152lGQ918152.1 Wiseana iridescent virus, complete genome. [29-AUG-2014]	90.635	162.5	98.8	205791	2	0.155983498	Wiseana iridescent virus:no rank
Faculty of Health and Medical Sciences 81	DQ890022lDQ890022.1 Paramecium bursaria Chlorella virus FR483, complete genome. [29-AUG-2014]	92.63714286	127.9285714	96.47714286	321240	14	0.534180052	Paramecium bursaria Chlorella virus FR483:no rank
Faculty of Health and Medical Sciences 81	HF920633lHF920633.1 Invertebrate iridovirus 22 complete genome [29-AUG-2014]	87.116	136.4	97.898	197693	5	0.327780953	Invertebrate iridovirus 22:species
Copenhagen University Hospital 56	KC961304lKC961304.1 Choristoneura rosaceana alphabaculovirus, complete genome. [29-AUG-2014]	80.77	141	53.19	129052	1	0.060440753	Choristoneura rosaceana alphabaculovirus:species
Copenhagen University Hospital 64	GQ918152lGQ918152.1 Wiseana iridescent virus, complete genome. [29-AUG-2014]	80	140	96.43	205791	1	0.065600536	Wiseana iridescent virus:no rank
Copenhagen University Hospital 64	HM004429lHM004429.1 Micromonas sp. RCC1109 virus MpV1, complete genome. [29-AUG-2014]	40.96	526	81.56	184095	1	0.26725332	Micromonas sp. RCC1109 virus MpV1:species
Copenhagen University Hospital 64	AY653733lAY653733.1 Acanthamoeba polyphaga mimivirus, complete genome. [29-AUG-2014]	88.46	214	36.45	1181404	1	0.006602314	Acanthamoeba polyphaga mimivirus:species
Copenhagen University Hospital 64	X02449lX02449.1 Hamster polyomavirus (HapV) genome [29-AUG-2014]	74.155	5936	20.57	5366	2	45.22922102	Hamster polyomavirus:species
Copenhagen University Hospital 64	GQ180114lGQ180114.1 Rattus norvegicus papillomavirus 1 EES-2009, complete genome. [29-AUG-2014]	100	145	99.31	7378	1	1.951748441	Rattus norvegicus papillomavirus 1 EES-2009:no rank
Copenhagen University Hospital 64	DQ890022lDQ890022.1 Paramecium bursaria Chlorella virus FR483, complete genome. [29-AUG-2014]	88.91	121	97.59	321240	5	0.187710123	Paramecium bursaria Chlorella virus FR483:no rank
Copenhagen University Hospital 65	EF101928lEF101928.1 Acanthocystis turfacea Chlorella virus 1, complete genome. [29-AUG-2014]	55.96	861	96.52	288047	1	0.282245606	Acanthocystis turfacea Chlorella virus 1:species
Copenhagen University Hospital 65	D10032lD10032.1 Baboon endogenous virus strain M7 proviral DNA, complete genome. [29-AUG-2014]	78.57	128	98.44	8507	1	1.481133184	Baboon endogenous virus strain M7:no rank
Copenhagen University Hospital 65	GQ918152lGQ918152.1 Wiseana iridescent virus, complete genome. [29-AUG-2014]	92.31	159	98.11	205791	1	0.075805064	Wiseana iridescent virus:no rank
Copenhagen University Hospital 65	X00255lX00255.1 Gardner-Rasheed feline sarcoma virus (GR-FeSV) proviral genome coding for P70 gag-fgr p	96	114	65.79	2025	1	3.703703704	Feline sarcoma virus:no rank
Copenhagen University Hospital 65	GQ222416lGQ222416.1 Orcinus orca endogenous retrovirus, complete genome. [29-AUG-2014]	80.43	154	89.61	8273	1	1.668076877	Orcinus orca:species
Copenhagen University Hospital 65	AF303741lAF303741.1 Chilo iridescent virus complete genome. [29-AUG-2014]	95.35	131	98.47	212482	1	0.060711025	Invertebrate iridescent virus 6:species
Copenhagen University Hospital 65	AF053745lAF053745.1 Mus dunni endogenous virus complete genome. [29-AUG-2014]	61.54	191	87.96	8655	1	2.253032929	Mus dunni endogenous virus:no rank
Copenhagen University Hospital 65	X02449lX02449.1 Hamster polyomavirus (HapV) genome [29-AUG-2014]	54.31	8132	33.57	5366	1	49.98136414	Hamster polyomavirus:species
Copenhagen University Hospital 65	DQ890022lDQ890022.1 Paramecium bursaria Chlorella virus FR483, complete genome. [29-AUG-2014]	98.935	138	97.855	321240	2	0.084049309	Paramecium bursaria Chlorella virus FR483:no rank
Copenhagen University Hospital 65	JX867617lJX867617.1 Murid herpesvirus 8 isolate England, complete genome. [29-AUG-2014]	100	132	100	202946	1	0.065041932	Murid herpesvirus 8:species
Copenhagen University Hospital 66	X02449lX02449.1 Hamster polyomavirus (HapV) genome [29-AUG-2014]	78.90333333	250	97.98	5366	3	13.69735371	Hamster polyomavirus:species
Copenhagen University Hospital 67	HM049560lHM049560.1 Murine adenovirus 2 isolate K87, complete genome. [29-AUG-2014]	96.97	108.5	98.095	35203	2	0.605062069	Murine adenovirus 2:no rank
Copenhagen University Hospital 67	JQ303225lJQ303225.1 Rhinolophus ferrumequinum retrovirus isolate RfRV, complete genome. [02-JUL-201	79.07	132	97.73	8389	1	1.537727977	Rhinolophus ferrumequinum retrovirus:no rank
Copenhagen University Hospital 67	D10032lD10032.1 Baboon endogenous virus strain M7 proviral DNA, complete genome. [29-AUG-2014]	79.715	125.5	98.02	8507	2	2.891736217	Baboon endogenous virus strain M7:no rank
Copenhagen University Hospital 67	AF033815lAF033815.1 Mason-Pfizer monkey virus, complete genome. [29-AUG-2014]	64.24	501	98.8	8557	1	5.784737642	Mason-Pfizer monkey virus:species
Copenhagen University Hospital 67	AF053745lAF053745.1 Mus dunni endogenous virus complete genome. [29-AUG-2014]	76.335	202.5	78.665	8655	2	3.500866551	Mus dunni endogenous virus:no rank
Copenhagen University Hospital 67	JQ619843lJQ619843.1 Human herpesvirus 8 strain DG1, complete genome. [29-AUG-2014]	93.48	153	90.2	135890	1	0.101552726	Human herpesvirus 8:species
Copenhagen University Hospital 67	AF033807lAF033807.1 Mouse mammary tumor virus complete proviral genome. [29-AUG-2014]	80.63	140.5	99.365	8805	2	3.168654174	Mouse mammary tumor virus:species
Copenhagen University Hospital 67	X02449lX02449.1 Hamster polyomavirus (HapV) genome [29-AUG-2014]	75.32333333	480	96.54	5366	3	22.19530376	Hamster polyomavirus:species
Copenhagen University Hospital 67	DQ890022lDQ890022.1 Paramecium bursaria Chlorella virus FR483, complete genome. [29-AUG-2014]	82.5	146.5	98.185	321240	2	0.096189765	Paramecium bursaria Chlorella virus FR483:no rank
Malaysia 75	AY388617lAY388617.1 Bovine adeno-associated virus, complete genome. [29-AUG-2014]	80.28	233	91.42	4693	1	4.538674622	Bovine adeno-associated virus:no rank
Malaysia 75	JN704610lJN704610.1 Meles meles fecal virus isolate VS4700006, complete genome. [29-AUG-2014]	72.99	419	98.09	2198	1	18.69881711	Meles meles fecal virus:species
Malaysia 75	DQ335246lDQ335246.2 Adeno-associated virus-Go.1, complete genome. [29-AUG-2014]	85.71	127	99.21	4636	1	2.717860224	Adeno-associated virus-Go.1:no rank
Malaysia 75	JQ037754lJQ037754.1 Artibeus jamaicensis parvovirus 1, complete genome. [29-AUG-2014]	66.67	244	99.59	4595	1	5.28835691	Artibeus jamaicensis parvovirus 1:species
Malaysia 76	HM049560lHM049560.1 Murine adenovirus 2 isolate K87, complete genome. [29-AUG-2014]	82.66	135.3333333	97.55333333	35203	3	1.141948129	Murine adenovirus 2:no rank
Kuala Langat 77	JN704610lJN704610.1 Meles meles fecal virus isolate VS4700006, complete genome. [29-AUG-2014]	69.75	497	95.98	2198	1	22.11101001	Meles meles fecal virus:species
Kuala Langat 77	DQ335246lDQ335246.2 Adeno-associated virus-Go.1, complete genome. [29-AUG-2014]	86.67	101	89.11	4636	1	1.941328732	Adeno-associated virus-Go.1:no rank
Kuala Langat 77	AF192260lAF192260.1 Periplaneta fuliginosa densovirus, complete genome. [29-AUG-2014]	84.4	425	99.53	5454	1	7.755775578	Periplaneta fuliginosa densovirus:no rank
