# Supplementary material for: High diversity of picornaviruses in rats from different continents revealed by deep sequencing
Source: Emerg Microbes Infect. 2016 Aug 17;5(8):e90–. doi: 10.1038/emi.2016.90 (PMC5034103; doi:10.1038/emi.2016.90)
Supplement: Supplementary Table 2 [file emi201690x4.pdf]

Supplementary Table S2 Metabarcoding analysis of rat faeces.

| | Plant family | Plant genus/tribe | Denmark samples | | | Malaysia samples | | |
|---|---|---|---|---|---|---|---|---|
| | | | Egedal | Copenhagen University Hospital | Botanical Garden of Copenhagen | Kuala Lumpur | Kuala Lumpur | Kuala Lumpur |
| Plant (trnL c/h) Data | Annonaceae | | | | | ✔ | | |
| | Apiaceae | | | | | | | ✔ |
| | Asteraceae | | | ✔ | ✔ | | | |
| | Betulaceae | | | ✔ | | | | |
| | Brassicaceae | | ✔ | ✔ | | | | |
| | Cupressaceae | *Platycladus* | | | | | | ✔ |
| | Euphoriaceae | | | | | | | ✔ |
| | Fabaceae | *Glycine* | ✔ | | | ✔ | | |
| | | *Arachis* | | ✔ | | | | |
| | Lamiaceae | *Mentha* | | | | | ✔ | |
| | Linaceae | *Linum* | | ✔ | ✔ | | | |
| | Malvaceae | *Theobroma* | | ✔ | ✔ | | | |
| | Moraceae | *Ficus* | | | | ✔ | | |
| | Piperaceae | *Piper* | | | | ✔ | | ✔ |
| | Plantaginaceae | *Scoparia* | | | | ✔ | | |
| | Poaceae | PACMAD clade | ✔ | ✔ | | | | |
| | | Triticeae | ✔ | ✔ | ✔ | ✔ | ✔ | |
| | | *Avena* | | | ✔ | | | |
| | | *Oryza* | | | ✔ | | ✔ | ✔ |
| | Polygonaceae | | ✔ | | | | | |
| | Rosaceae | | | ✔ | | | | |
| | | *Prunus* | | | ✔ | | | |
| | Salicaceae | *Salix* | ✔ | | | | | |
| | Sapindaceae | *Acer* | ✔ | | ✔ | | | |
| | | | | | | ✔ | | ✔ |
| | Solanaceae | | | | | | ✔ | ✔ |
| | Urticaceae | | | | | ✔ | | |
| | Vitaceae | | | ✔ | ✔ | | | ✔ |
| Insect (Mzart COI) Data | **Order/ family** | **Subfamily/genus** | | | | | | |
| | Porcellionidae | *Porcellio* | ✔ | | | | | |
| | Saturniidae | Hemileucinae | ✔ | | | | | |
| | Coleoptera | | ✔ | | | | | |
| | Tachinidae | | ✔ | | | | | |
| | Hymenoptera | | | ✔ | | | | |
| | Rhabditida | (strongylida) | | ✔ | | | | |
| | Leptoceridae | | | | | | ✔ | |
| | Philodinidae | *Rotaria* | | | | | ✔ | ✔ |
| | Rhodymeniaceae | | | | | | | ✔ |
| | Phoxocephalidae | | | | | | | ✔ |
| 16sMam D | **Family** | **Genus/species** | | | | | | |
| | Muridae | *Rattus norvegicus* | ✔ | ✔ | ✔ | ✔ | ✔ | ✔ |
| | Bovidae | *Bos* | ✔ | | | | | |
| | | *Ovis* | | | | | | ✔ |
