# Supplementary material for: High diversity of picornaviruses in rats from different continents revealed by deep sequencing
Source: Emerg Microbes Infect. 2016 Aug 17;5(8):e90–. doi: 10.1038/emi.2016.90 (PMC5034103; doi:10.1038/emi.2016.90)
Supplement: Supplementary Table 3 [file emi201690x5.pdf]

Supplementary Table S3 Primers used for PCR validation

Oligo name	Sequence of PCR primer (5' →3')	Confirmed mapping of sanger sequence§
RodSCV_amgr_F	GACCCATATGTTGGAGCACT	+
RodSCV_amgr_R	CTCTTCGAGCTCGTTCATTC	+
HTMEV_amgr_F	GAACAACACAGGACATGCAA	+
HTMEV_amgr_R	AGATGAGACAGCGGAATCAG	+
Himet_amgr_F	CTAATGAGAGAGGCGCAAAC	+
Himet_amgr_R	CCATTCATTACCCAGTGGAC	+
Provi_ege21_F	TTACCGAACACGTTCCAAAT	+
Provi_ege21_R	TGAATGAAAATCCGACACCT	+
HTMEV_ege21_F	ATAGTCATGGGGTGAAGCAA	+
HTMEV_ege21_R	ACCCACATCAACTGCTGAAT	+
Provi_ege23_F	CAGGTGTCGGATTTTCATTC	
Provi_ege23_R	ACCCTCACCTGTCATTTCAA	
Minio_ege24_F	GGAAGAACAAGGTGCTCTGA	+
Minio_ege24_R	CTTTTCCATTGCATCCATTC	+
BatCir_Geo60_F	CGCAAGATGTCGTATCTCCT	
BatCir_Geo60_R	TTTACCCCGTGGCTAATGTA	
BocaGeo60_F	CGGTCTGGTTCTGTGATACC	
BocaGeo60_R	GGTTGCTAACCTGCAAAGAA	
sclsclGeo60_F	AGTTTGCTGAGCACATTTCC	+
sclsclGeo60_R	ACGAACACGTGGAGATGAAT	+
Minio_Geo60_F	GGAAAAAGTACAGGCAGCAA	+
Minio_Geo60_R	TTGGTACCTTGCATCCTCAT	+
DFcycGe060_F	CGTAGATCGCCTTGAACACT	+
DFcycGe060_R	TGCACGAAAGTGGTGTTTTA	
addGeo60_F	CGGACATCACGCTTTCTAGT	+
addGeo60_R	GCTCACCGTAGTCGACTTGT	+

§The "+" indicate that, in addition to a PCR product, the oligonucleotide produced a Sanger sequence mapping to the expected reference genome.
