# Supplementary material for: High diversity of picornaviruses in rats from different continents revealed by deep sequencing
Source: Emerg Microbes Infect. 2016 Aug 17;5(8):e90–. doi: 10.1038/emi.2016.90 (PMC5034103; doi:10.1038/emi.2016.90)
Supplement: Supplementary Information [file emi201690x6.pdf]

Supplementary Materials

((((((Equine_rhinitis_A_virus|gb|AFY10938.1|:0.0263445,((Equine_rhinitis_A_vir
us|gb|ABB76801.1|:0.00537248,((Equine_rhinitis_A_virus|gb|ABB76804.1|:0.00
701121,(('Equine rhinitis A virus|gb||':9.25469E-
7,Equine_rhinitis_A_virus|gb|AAB61952.1|:9.14769E-
4)100:0.0285755,Equine_rhinitis_A_virus|gb|ABB76802.1|:0.00630377)85:0.00
66832)44:0.00564135,Equine_rhinitis_A_virus|gb|ABB76803.1|:0.00804648)54:
0.00505896)99:0.0333638,(Equine_rhinitis_A_virus|gb|ABB76805.1|:0.0013745
6,(Equine_rhinitis_A_virus|gb|CAA65613.1|:9.25469E-
7,Equine_rhinitis_A_virus|gb|CAA65614.1|:9.25469E-
7)99:0.00209483)96:0.0206464)66:0.0127258)100:1.5091,((Equine_rhinitis_B_
virus_1|gb|CAA65615.1|:0.131315,Equine_rhinitis_B_virus_2|gb|AAK91591.1|:0.
0865741)100:2.45872,(((Mouse_Mosavirus|gb|AEM05833.1|:0.362151,Mosavir
us_A2|gb|AHN95561.1|:0.37465)100:2.23677,(((Porcine_teschovirus_1|gb|AAK
12417.1|:0.0409863,Porcine_teschovirus_1|gb|CAB40546.1|:0.0379896)100:0.0
661786,(Porcine_teschovirus_8|gb|AAK12387.1|:0.0698558,Porcine_teschovirus
_4|gb|AGB67759.1|:0.0775591)99:0.0444483)100:2.55385,((((Human_rhinovir
us_A38|gb|ACK37436.1|:0.00130958,Human_rhinovirus_A38|gb|ABF51189.1|:0.
00204119)100:0.133617,(Human_rhinovirus_1B|gb|BAA00168.1|:0.024033,(Hu
man_rhinovirus_A1|gb|ACK37367.1|:0.0148305,Rhinovirus_A|gb|AET25085.1|:0
.0342728)75:0.0248918)100:0.135622)100:0.818021,(Human_poliovirus_2|gb|
CAD67522.1|:0.633694,Enterovirus_B|gb|AGP25524.1|:0.51083)100:0.537565)
100:0.521602,query_AE_kloak_46-55_v2.contig-3323000012_3_|1_-
_294|FORWARD_SENSE:1.18585):2.43568)93:0.534815)85:0.325533,((((Huma
n_cosavirus_B1|gb|ACL15190.1|:0.490083,(Human_cosavirus_E1|gb|ACL99815.
1|:0.308501,(Human_cosavirus_E/D|gb|AFJ04538.1|:0.124447,(Human_cosaviru
s_D1|gb|ACL15191.1|:0.123722,Human_cosavirus|gb|AHV83701.1|:0.0677401)
100:0.305237)100:0.157702)100:0.328057)60:0.17406,(Cosavirus_A|gb|ACL15
186.1|:0.111361,(Cosavirus_A|gb|ACL15185.1|:0.157403,((Cosavirus_A|gb|ADJ3
9692.1|:0.0226459,Human_cosavirus_A|gb|AHX26969.1|:0.0373761)100:0.1055
28,(Human_cosavirus_A20|gb|AFJ04537.1|:0.116256,Human_cosavirus_A19|gb|
AFJ04540.1|:0.116717)100:0.0702155)65:0.0386613)68:0.0305422)100:0.290
463)50:0.131286,Human_cosavirus_F|gb|AFJ04539.1|:0.431328)100:1.63985,(
Miniopterus_schreibersii_picornavirus_1|gb|AFK85007.1|:1.94843,((Seneca_vall
ey_virus|gb|ABG23522.1|:0.00799649,Seneca_valley_virus|gb|AGM16001.1|:0.0
149377)100:2.1554,((((((((((((((((((Boone_cardiovirus|gb|AGC69961.1|:9.7352
3E-6,query_CUH-57s.contig-7387000011_1_|3_-
_140|FORWARD_SENSE:6.11352E-
6):0.0131693,query__Boone_cardiovirus_isolate_Boone-NYC_NYC-
D10_polyprotein_gene__partial_cds_3_|1_-
_960|FORWARD_SENSE:0.00489865):0.0433752,query_EM_24_v2.contig-
27941000013_93_|6301_-
_146|REVERSE_SENSE:0.0546797):0.0106122,query_Kuala_Lumpur_68s_v2.cont
ig-8000017_59_|1339_-
_8340|FORWARD_SENSE:0.0477474):0.00944699,query_AE_kloak_46-
55_v2.contig-2000011_117_|5441_-
_3|REVERSE_SENSE:0.0997053):0.0117448,query_EM_21_v2.contig-

1000015_31_|2213_-_3|REVERSE_SENSE:0.101339):1.6923E-
4,query_Kuala_Langat_v2.contig-8513000015_4_|189_-
_1|REVERSE_SENSE:6.11352E-6):0.0107482,query_CUH_56-DNAse.contig-
11916000005_5_|280_-_2|REVERSE_SENSE:6.11352E-
6):0.00632829,query_BGC_60s_v2.contig-96000006_100_|4750_-
_2|REVERSE_SENSE:0.0872731):0.0178604,query_BGC_61s_v2.contig-
10699000013_4_|3_-
_488|FORWARD_SENSE:0.048909):0.0225584,query_Kuala_Lumpur_69s.contig-
8178000009_4_|2_-_418|FORWARD_SENSE:8.79979E-
6):0.00132027,query_Sachsenroder_SRR1438002.contig-2115000010_3_|3_-
_431|FORWARD_SENSE:0.124415):0.00209737,query_BGC_60s_v2.contig-
40819000015_6_|406_-_2|REVERSE_SENSE:9.7914E-
6):1.12658,((((Encephalomyocarditis_virus_type_2|gb|AFO66759.1|:0.175012,((
Encephalomyocarditis_virus|gb|AGU38151.1|:9.25469E-
7,Encephalomyocarditis_virus|gb|AGU38152.1|:0.00126741)100:0.109177,(((En
cephalomyocarditis_virus|gb|AAA43035.1|:0.00191136,(Encephalomyocarditis_
virus|gb|CAA60776.1|:0.00127858,(Encephalomyocarditis_virus|gb|AAA43034.
1|:6.37145E-4,(Encephalomyocarditis_virus_strain_emc-
b_nondiabetogenic|gb|P17593.1|:9.25469E-
7,Encephalomyocarditis_virus|gb|AAA43033.1|:0.00128115)100:0.00321204)9
9:0.00385067)88:0.00129773)100:0.0323796,(Encephalomyocarditis_virus|gb|
AAP51180.1|:0.029492,(Encephalomyocarditis_virus|gb|CAA52361.1|:0.005023
53,((((Encephalomyocarditis_virus|gb|AAA43037.1|:6.21236E-
4,Encephalomyocarditis_virus|gb|CAA25152.1|:0.0423415)100:0.00578068,Enc
ephalomyocarditis_virus|gb|ABC25550.1|:9.25469E-
7)97:0.00312237,Encephalomyocarditis_virus|gb|AHF20227.1|:0.00264285)21:
0.00127761,(Encephalomyocarditis_virus|gb|AAL83502.1|:0.00256403,((Porcin
e_encephalomyocarditis_virus|gb|ABG20637.1|:9.25469E-
7,(Encephalomyocarditis_virus|gb|ACI47518.1|:0.00578294,Encephalomyocardi
tis_virus|gb|ACI47517.1|:0.00384476)67:6.35933E-
4)90:0.00127852,(Encephalomyocarditis_virus|gb|AHX84176.1|:6.383E-
4,((((Encephalomyocarditis_virus|gb|ACM45091.1|:6.39193E-
4,Encephalomyocarditis_virus|gb|AHF20225.1|:0.00255892)76:0.00127827,(En
cephalomyocarditis_virus|gb|AHF20226.1|:6.38509E-
4,Encephalomyocarditis_virus|gb|ACM45090.1|:9.25469E-7)77:6.38587E-
4)70:0.00127725,((((Encephalomyocarditis_virus|gb|ABE77396.1|:9.25469E-
7,(Encephalomyocarditis_virus|gb|AHF20224.1|:0.00127811,Encephalomyocard
itis_virus|gb|AHF20223.1|:6.39337E-
4)68:0.0012792)90:0.00384303,(Encephalomyocarditis_virus|gb|AHA43044.1|:
0.00385819,(Encephalomyocarditis_virus|gb|ABE77395.1|:6.38859E-
4,Encephalomyocarditis_virus|gb|AHA43045.1|:0.00577161)18:9.25469E-
7)29:6.38877E-4)8:9.25469E-
7,(Porcine_encephalomyocarditis_virus|gb|ADN52625.1|:6.39199E-
4,((Encephalomyocarditis_virus|gb|AGS11291.1|:0.00643743,(Encephalomyocar
ditis_virus|gb|AHA43046.1|:0.00320787,(Encephalomyocarditis_virus|gb|AHA4
3047.1|:0.00258603,Encephalomyocarditis_virus|gb|AHA43048.1|:0.00450294)
45:9.25469E-7)50:9.25469E-7)27:6.39689E-
4,Encephalomyocarditis_virus|gb|AGY30767.1|:6.38949E-4)4:9.25469E-
7)10:6.39403E-4)16:6.3988E-

4,(Encephalomyocarditis_virus|gb|ACQ90253.1|:0.0031962,Encephalomyocardit
is_virus|gb|AHF20222.1|:9.25469E-7)98:0.00319677)2:9.25469E-
7)13:6.38195E-
4,Encephalomyocarditis_virus|gb|AFU64561.1|:0.001277)5:9.25469E-
7)16:6.37557E-4)40:0.00127868)38:7.23292E-4)43:6.85094E-
4)100:0.0213356)79:0.007813)100:0.0182471,(Encephalomyocarditis_virus|gb
|ABI15777.2|:0.0291352,(Mengo_virus|gb|AAA46547.1|:0.00115699,Mengo_vir
us|gb|ABB97066.1|:1.21265E-
4)100:0.0485374)57:0.0107744)100:0.0971334)94:0.0527982)100:0.287415,q
uery_AE_kloak_46-55_v2.contig-4256000003_2_|3_-
_167|FORWARD_SENSE:6.11352E-6):0.239903,query_CUH_56-DNAse.contig-
3832000002_3_|3_-_185|FORWARD_SENSE:6.11352E-
6):0.187122,((((((((((Theilers_encephalomyelitis_virus|gb|AGM61326.1|:0.002
58578,(Theilers_encephalomyelitis_virus|gb|AAA47928.1|:0.00254115,Theilers_
encephalomyelitis_virus|gb|AHH60910.1|:0.00127071)99:0.00250852)100:0.02
48876,(Theilers_encephalomyelitis_virus|gb|AEC04618.1|:0.0339501,((Theilers_
encephalomyelitis_virus|gb|AAA47929.1|:0.00252848,'Theilers_encephalomyelit
is_virus_(STRAIN_GDVII)|gb|CAA39496.1|':6.30122E-
4)100:0.0326966,(Theilers_encephalomyelitis_virus|gb|ACG55802.1|:0.0323944
,(Theilers_encephalomyelitis_virus|gb|ACG55799.1|:0.025604,(Theilers_encepha
lomyelitis_virus|gb|AAA47930.1|:0.00383896,Theilers_encephalomyelitis_virus|
gb|ABD67451.1|:9.25469E-
7)100:0.0184244)70:0.00889929)56:0.00506645)74:0.00961667)78:0.015103
2)91:0.0132833,(Theilers_encephalomyelitis_virus|gb|ACG55800.1|:0.045222,Si
khote-
Alin_virus|gb|AHW57724.1|:0.0329055)60:0.00823982)100:0.0669204,query_C
UH_64-67_pool.contig-1831000009_8_|613_-
_2|REVERSE_SENSE:0.247869):0.0194233,query_AE_kloak_46-55_v2.contig-
6869000000_1_|3_-
_119|FORWARD_SENSE:0.167842):0.0408007,query_Kuala_Lumpur_75ps-
76_v2.contig-44000014_18_|1047_-
_4|REVERSE_SENSE:0.197078):0.00208767,query_Kuala_Lumpur_75ps-
76_v2.contig-925000017_6_|3_-
_1001|FORWARD_SENSE:0.161082):0.0057426,query_CUH_64-67_pool.contig-
50000011_3_|218_-
_3|REVERSE_SENSE:0.180455):0.0303648,(((((((((((((((Saffold_virus|gb|AFP81
729.1|:0.00123803,Saffold_virus|gb|AFP81731.1|:0.00371565)8:9.25469E-
7,((Saffold_virus|gb|AFP81727.1|:0.00123733,Saffold_virus|gb|AFP81728.1|:0.0
0495789)70:6.1894E-
4,Saffold_virus|gb|AFP81730.1|:0.00123792)66:6.18432E-
4)100:0.0173078,(Saffold_virus|gb|ABP68884.2|:0.0191514,Saffold_virus|gb|BA
M44706.1|:0.00551531)59:0.0039801)100:0.04688,(Saffold_virus|gb|BAM4470
9.1|:0.0456517,(Saffold_virus|gb|BAM44707.1|:0.0195256,((Saffold_virus|gb|CB
W45996.2|:0.0173724,Saffold_virus|gb|ADO20363.1|:0.0210667)100:0.016719
8,((Cardiovirus_BR/118/2006|gb|ACG61136.2|:0.0224743,(Human_TMEV-
like_cardiovirus|gb|ADF28539.1|:0.0130208,(Human_TMEV-
like_cardiovirus|gb|ACB29695.1|:0.00221786,(Saffold_virus|gb|CAP58274.1|:0.0
0318614,Saffold_virus|gb|AEE69077.1|:0.00442426)100:0.00736942)100:0.015
5581)45:0.00403519)48:0.00349684,(Saffold_virus_2|gb|AFP86294.1|:0.01616

9,(Cardiovirus_D/VI2229/2004|gb|ACG61135.2|:0.00439,(Saffold_virus|gb|CBS9
1673.1|:0.00564336,(Saffold_virus|gb|AER13151.1|:0.00187862,(Saffold_virus|g
b|AER13150.1|:6.25588E-4,Saffold_virus|gb|AER13152.1|:9.25469E-
7)98:0.00187873)100:0.00250669)73:6.25493E-
4)100:0.00487947)23:0.00202959)53:0.00469682)77:0.00791725)100:0.0383
363)95:0.0258519)97:0.024249,((((Saffold_virus|gb|BAM44708.1|:0.0127889,(
Saffold_virus|gb|ADK91813.1|:0.0176974,((((Saffold_virus|gb|CAR62533.1|:0.00
930279,Saffold_virus|gb|ADO20359.1|:0.0105347)31:5.98591E-
4,(Saffold_virus|gb|ADO20358.1|:0.00432345,Saffold_virus|gb|AEK80410.1|:0.0
0308341)78:6.12976E-
4)42:0.00158009,Saffold_virus|gb|AEM00022.1|:0.00710158)99:0.00711682,((
Cardiovirus_D/VI2273/2004|gb|ACG61137.2|:0.00431069,Cardiovirus_D/VI222
3/2004|gb|ACG61138.2|:6.81007E-
4)100:0.00921389,(Saffold_virus|gb|ADK91816.1|:0.00584871,(Saffold_virus|gb
|ADK91815.1|:0.00370693,Saffold_virus|gb|ADK91814.1|:0.00309084)96:0.001
55614)100:0.00870265)95:0.00649615)85:0.00406692)74:0.00939083)100:0.
0371135,(Saffold_virus|gb|BAM44713.1|:0.0371638,Saffold_virus|gb|BAM4471
6.1|:0.0481594)98:0.0313626)89:0.0244794,((Saffold_virus|gb|BAM44712.1|:0.
062683,(Saffold_virus|gb|BAM44714.1|:0.0487573,Saffold_virus|gb|BAM44715.
1|:0.0506526)100:0.0386818)97:0.00691533,query_Kuala_Lumpur_75ps-
76_v2.contig-11000015_3_|1_-
_387|FORWARD_SENSE:0.131569):0.0145682)99:0.0341513,(((((Saffold_virus|
gb|ACO92357.1|:0.0150259,Saffold_virus|gb|BAM44711.1|:0.00319491)100:0.0
435511,(Saffold_virus|gb|BAM44710.1|:0.00696227,(Saffold_virus|gb|ACO9235
5.1|:0.00556904,Saffold_virus|gb|ACO92353.1|:0.017964)52:9.68695E-
4)100:0.0345578)100:0.0297307,query_Kuala_Lumpur_75ps-76_v2.contig-
978000006_2_|1_-
_300|FORWARD_SENSE:0.0371699):0.00752416,query_Sachsenroder_SRR1438
012.contig-1787000011_2_|1_-
_165|FORWARD_SENSE:0.145282):0.00554038,query_Kuala_Lumpur_75ps-
76_v2.contig-9000003_4_|2_-
_517|FORWARD_SENSE:0.155742):0.0180681)48:0.0118974)100:0.150894,que
ry_Kuala_Lumpur_75ps-76_v2.contig-6988000004_10_|524_-
_3|REVERSE_SENSE:0.0839071):0.0468944,query_CUH_56-DNAse.contig-
7347000012_1_|1_-_267|FORWARD_SENSE:6.11352E-
6):0.0291922,query_AE_kloak_46-55_v2.contig-5341000014_4_|3_-
_377|FORWARD_SENSE:6.11352E-
6):0.0804006,((((((((((((((((((((((((((((((((((((((Theilers-
like_virus_of_rats|gb|BAC58035.1|:7.70161E-6,query_CUH_56-DNAse.contig-
4881000006_1_|1_-_162|FORWARD_SENSE:0.0309692):0.0,query_CUH_56-
DNAse.contig-1283000017_4_|317_-
_3|REVERSE_SENSE:0.0715511):0.00445767,query_CUH-57s.contig-
16399000000_1_|2_-_100|FORWARD_SENSE:0.0788011):2.66522E-
4,query_CUH_56-DNAse.contig-30668000013_2_|3_-
_104|FORWARD_SENSE:0.0292671):0.00286567,query_AE_kloak_46-
55_v2.contig-10325000007_2_|1_-
_303|FORWARD_SENSE:0.134993):0.00125744,query_AE_kloak_46-
55_v2.contig-8485000011_10_|383_-
_3|REVERSE_SENSE:0.111487):0.00691789,((((((((Rat_theilovirus_1|gb|ACD678

70.1|:6.87089E-6,query_CUH_64-67_pool.contig-480000005_3_|1_-
_246|FORWARD_SENSE:6.11352E-6):0.0,query_Hong_kong_71-73_pool.contig-
9443000011_2_|116_-_3|REVERSE_SENSE:6.11352E-6):0.0,query_CUH_64-
67_pool.contig-2284000011_4_|154_-
_2|REVERSE_SENSE:0.0264227):0.0,query_CUH_56-DNAse.contig-
2022000011_4_|199_-_2|REVERSE_SENSE:6.11352E-6):0.0,query_CUH_56-
DNAse.contig-1661000003_2_|1_-
_339|FORWARD_SENSE:0.0521171):0.0,query_AE_kloak_46-55_v2.contig-
2743000012_2_|2_-
_199|FORWARD_SENSE:0.113671):0.0,query_Kuala_Langat_v2.contig-
6286000007_2_|122_-_3|REVERSE_SENSE:6.11352E-
6):0.00702892,((((((Rat_theilovirus_1|gb|ACF19652.1|:6.46151E-
6,query_Kuala_Lumpur_75ps-76_v2.contig-2532000011_4_|3_-
_332|FORWARD_SENSE:0.0675477):0.0,query_CUH_56-DNAse.contig-
9190000003_1_|157_-_2|REVERSE_SENSE:0.154016):0.00590507,query_CUH-
57s.contig-9321000015_2_|128_-_3|REVERSE_SENSE:0.037372):7.72947E-
5,query_CUH-57s.contig-297000000_3_|3_-
_539|FORWARD_SENSE:0.0520163):1.81571E-4,query_AE_kloak_46-
55_v2.contig-20000006_4_|3_-_425|FORWARD_SENSE:0.0687172):3.71116E-
4,query_Kuala_Lumpur_75ps-76_v2.contig-6819000005_2_|3_-
_218|FORWARD_SENSE:0.0445473):0.00669167)49:0.00588258)100:7.17904E-
6,query_Kuala_Lumpur_75ps-76_v2.contig-4598000004_3_|128_-
_3|REVERSE_SENSE:6.11352E-6):0.0129736,query_AE_kloak_46-55_v2.contig-
61000008_3_|365_-_3|REVERSE_SENSE:6.11352E-6):6.038E-6,query_CUH-
57s.contig-111000009_21_|1586_-
_3|REVERSE_SENSE:0.048617):0.00533094,query_AE_kloak_46-55_v2.contig-
12010000015_1_|1_-_270|FORWARD_SENSE:7.55436E-6):9.43726E-
4,query_AE_kloak_46-55_v2.contig-2483000006_2_|1_-
_297|FORWARD_SENSE:6.11352E-6):0.00599822,query_CUH_56-DNAse.contig-
14894000006_2_|127_-
_2|REVERSE_SENSE:0.0685625):0.0012677,query_AE_kloak_46-55_v2.contig-
12000016_7_|2_-_685|FORWARD_SENSE:6.11352E-
6):0.00267146,query_CUH_56-DNAse.contig-6076000003_5_|350_-
_3|REVERSE_SENSE:0.071035):0.00246678,query_CUH_56-DNAse.contig-
23960000009_3_|266_-_3|REVERSE_SENSE:6.11352E-6):1.337E-5,query_CUH-
57s.contig-1279000003_3_|263_-_3|REVERSE_SENSE:6.11352E-6):1.1864E-
4,query_Kuala_Lumpur_75ps-76_v2.contig-41000001_3_|1_-
_465|FORWARD_SENSE:0.0188265):0.00227186,query_CUH-57s.contig-
76000010_8_|3_-_1085|FORWARD_SENSE:0.0292796):8.13923E-
4,query_CUH_64-67_pool.contig-2607000010_4_|2_-
_526|FORWARD_SENSE:0.0220693):0.00442458,query_CUH_56-DNAse.contig-
1220000010_4_|2_-
_349|FORWARD_SENSE:0.0124241):0.0015938,query_Kuala_Lumpur_69s.contig
-276000007_46_|3522_-_1|REVERSE_SENSE:0.065144):7.91336E-
4,query_CUH_56-DNAse.contig-7558000004_2_|3_-
_164|FORWARD_SENSE:0.0600594):0.00209421,query_Kuala_Lumpur_69s.conti
g-1940000000_15_|1_-
_2085|FORWARD_SENSE:0.0528602):0.00115156,query_CUH-57s.contig-
296000006_5_|1_-_861|FORWARD_SENSE:0.0424233):6.32663E-

4,query_Kuala_Lumpur_70.contig-22000005_4_|241_-
_2|REVERSE_SENSE:6.11352E-6):6.353E-6,query_Kuala_Lumpur_75ps-
76_v2.contig-1949000017_4_|373_-_2|REVERSE_SENSE:6.11352E-6):5.97554E-
4,query_Kuala_Lumpur_75ps-76_v2.contig-24000007_11_|2_-
_1339|FORWARD_SENSE:0.0250192):7.47296E-
4,query_Kuala_Lumpur_69s.contig-859000001_8_|651_-
_112|REVERSE_SENSE:0.0292192):0.00151733,query_Sachsenroder_SRR14380
12.contig-2137000006_1_|2_-
_148|FORWARD_SENSE:0.237017):0.00209906,query_CUH_56-DNAse.contig-
10378000001_8_|626_-_3|REVERSE_SENSE:0.0414715):0.0046251,query_CUH-
57s.contig-121000015_6_|3_-
_887|FORWARD_SENSE:0.0755864):0.00364314,query_CUH_64-67_pool.contig-
3105000006_2_|104_-_3|REVERSE_SENSE:6.11352E-
6):0.00120269,query_Kuala_Lumpur_75ps-76_v2.contig-25000010_8_|156_-
_1301|FORWARD_SENSE:0.105413):9.329E-4,query_Kuala_Lumpur_70.contig-
754000004_1_|2_-_163|FORWARD_SENSE:0.0687958):0.010039,query_CUH-
57s.contig-193000010_15_|844_-
_2|REVERSE_SENSE:0.0620261):0.00100841,query_Kuala_Lumpur_75ps-
76_v2.contig-2784000001_4_|1_-
_681|FORWARD_SENSE:0.0557196):0.00334145,query_Kuala_Lumpur_69s.conti
g-332000013_4_|1_-
_534|FORWARD_SENSE:0.104616):0.0422823,query_Sachsenroder_SRR143801
2.contig-3507000010_1_|3_-_122|FORWARD_SENSE:0.0455688):7.18321E-
6)69:6.37339E-6,query_Kuala_Lumpur_70.contig-2277000008_2_|3_-
_152|FORWARD_SENSE:6.11352E-6):0.0250418,query_CUH_56-DNAse.contig-
3220000000_4_|210_-
_1|REVERSE_SENSE:0.0857904):0.00269376,query_Hong_kong_71-
73_pool.contig-2691000006_2_|2_-_151|FORWARD_SENSE:6.11352E-
6):0.0249864,query_CUH_64-67_pool.contig-746000002_4_|267_-
_1|REVERSE_SENSE:6.11352E-
6):0.0138741,query_Kuala_Lumpur_68s_v2.contig-1022000007_48_|940_-
_7623|FORWARD_SENSE:0.124174):0.00501167,query_Kuala_Lumpur_75ps-
76_v2.contig-17000013_6_|3_-
_1172|FORWARD_SENSE:0.203644):0.0023312,query_Kuala_Lumpur_75ps-
76_v2.contig-40000009_18_|942_-
_10|REVERSE_SENSE:0.174344):0.0304764)100:0.0348684,query_CUH_64-
67_pool.contig-7000010_2_|3_-
_401|FORWARD_SENSE:0.144418):0.123147,query_Kuala_Lumpur_78.contig-
27881000000_3_|106_-_2|REVERSE_SENSE:6.11352E-
6):0.415381)100:0.387038)100:6.41913E-6,query_EM_21_v2.contig-
2000008_11_|3_-
_1946|FORWARD_SENSE:0.0972192):0.0,query_BGC_61s_v2.contig-
1357000001_5_|356_-_3|REVERSE_SENSE:0.265468):0.0,query_AE_kloak_46-
55_v2.contig-0_14_|2_-
_2395|FORWARD_SENSE:0.110894):0.0,query_EM_21_v2.contig-
1000010_24_|1520_-
_3|REVERSE_SENSE:0.0955716):0.0,query_BGC_60s_v2.contig-
74403000011_14_|1_-
_2559|FORWARD_SENSE:0.0938024):0.420678)37:0.165935)71:0.321361)95:0.

346886)91:0.389869)100:0.832516)100:0.819398,(Bovine_rhinitis_B_virus|gb|
ACA05181.1|:1.01763,Bovine_rhinitis_A_virus|gb|AFC40221.1|:1.02478)95:0.30
4709)100:0.943366,(((((Foot-and-mouth_disease_virus_-
_type_SAT_3|gb|AAT01793.1|:0.0375902,(Foot-and-mouth_disease_virus_-
_type_SAT_3|gb|AAT01796.1|:0.0209824,(Foot-and-mouth_disease_virus_-
_type_SAT_3|gb|AAT01794.1|:0.00201584,Foot-and-mouth_disease_virus_-
_type_SAT_3|gb|AAT01795.1|:9.25469E-
7)100:0.0386983)100:0.0136341)100:0.0743469,(((Foot-and-
mouth_disease_virus_-_type_SAT_1|gb|AFE84744.1|:0.0389392,Foot-and-
mouth_disease_virus_-
_type_SAT_1|gb|AAT01789.1|:0.0379263)69:0.0128284,Foot-and-
mouth_disease_virus_-
_type_SAT_1|gb|ADI24382.1|:0.167707)74:0.0110731,(((Foot-and-
mouth_disease_virus_-_type_SAT_1|gb|AAT01783.1|:0.023728,Foot-and-
mouth_disease_virus_-
_type_SAT_1|gb|AAT01784.1|:0.0178912)53:0.00444472,Foot-and-
mouth_disease_virus_-
_type_SAT_1|gb|AAT01781.1|:0.0431778)34:0.00269886,((Foot-and-
mouth_disease_virus_-_type_SAT_1|gb|AAT01786.1|:0.0181685,Foot-and-
mouth_disease_virus_-
_type_SAT_1|gb|AAT01788.1|:0.0161904)86:0.0058179,Foot-and-
mouth_disease_virus_-
_type_SAT_1|gb|AAT01785.1|:0.015803)42:0.00327395)99:0.0151717)78:0.063
9229)79:0.0891443,(Foot-and-mouth_disease_virus_-
_type_SAT_2|gb|AFE84748.1|:0.0336598,(Foot-and-mouth_disease_virus_-
_type_SAT_2|gb|AAT01790.1|:0.0388356,Foot-and-mouth_disease_virus_-
_type_SAT_2|gb|AAQ11227.1|:0.0360078)37:0.00545343)80:0.00930868)78:0.1
24389,(Foot-and-mouth_disease_virus_-
_type_SAT_2|gb|ADI24380.1|:0.028076,(Foot-and-mouth_disease_virus_-
_type_SAT_2|gb|ADI24381.1|:0.0223506,Foot-and-mouth_disease_virus_-
_type_SAT_2|gb|AFE84746.1|:0.019343)73:0.00700506)98:0.0331401)93:0.068
4274,Foot-and-mouth_disease_virus_-
_type_SAT_2|gb|AAT01792.1|:0.0487713)99:0.15297)100:0.120783,((((Foot-
and-mouth_disease_virus_-_type_Asia_1|gb|AEQ49430.1|:0.0124233,Foot-and-
mouth_disease_virus_-
_type_Asia_1|gb|AEQ49431.1|:0.0204883)100:0.010565,(((((((Foot-and-
mouth_disease_virus_-_type_Asia_1|gb|ABI93981.1|:0.0170484,Foot-and-
mouth_disease_virus_-
_type_Asia_1|gb|ABI93982.1|:0.012457)85:0.00290472,Foot-and-
mouth_disease_virus_-
_type_Asia_1|gb|ABI93984.1|:0.0289841)22:0.00300361,(Foot-and-
mouth_disease_virus_-_type_Asia_1|gb|ADX97245.1|:0.0243181,Foot-and-
mouth_disease_virus_-
_type_Asia_1|gb|AAU00941.1|:0.0235459)19:0.00339262)9:0.00202179,((Foot-
and-mouth_disease_virus_-_type_Asia_1|gb|ABI93992.1|:0.00987617,(Foot-and-
mouth_disease_virus_-_type_Asia_1|gb|ABI93990.1|:0.00942868,Foot-and-
mouth_disease_virus_-
_type_Asia_1|gb|ABI93995.1|:0.01822)94:0.00609047)68:0.00600137,((Foot-
and-mouth_disease_virus_-_type_Asia_1|gb|ABI93989.1|:0.0172961,Foot-and-

mouth_disease_virus_-
_type_Asia_1|gb|ABI93985.1|:0.0314385)33:0.00576104,(Foot-and-
mouth_disease_virus_-_type_Asia_1|gb|ABI93978.1|:0.0178669,Foot-and-
mouth_disease_virus_-
_type_Asia_1|gb|ABI93988.1|:0.0203243)45:0.00364452)7:0.00272977)2:5.841
62E-4)53:0.002762,(Foot-and-mouth_disease_virus_-
_type_Asia_1|gb|AEO22161.1|:0.00135272,Foot-and-mouth_disease_virus_-
_type_Asia_1|gb|AAT01743.1|:9.25469E-
7)100:0.0236428)52:0.00502738,((Foot-and-mouth_disease_virus_-
_type_Asia_1|gb|AAT01738.1|:0.0268118,(Foot-and-mouth_disease_virus_-
_type_Asia_1|gb|ABM66095.1|:0.0116215,(Foot-and-mouth_disease_virus_-
_type_Asia_1|gb|ABV03522.1|:0.00179061,((Foot-and-mouth_disease_virus_-
_type_Asia_1|gb|ACP44144.1|:0.00610243,(Foot-and-mouth_disease_virus_-
_type_Asia_1|gb|ADB28902.1|:9.25469E-7,Foot-and-mouth_disease_virus_-
_type_Asia_1|gb|ADC92543.1|:0.00135363)97:0.00338704)7:9.25469E-7,Foot-
and-mouth_disease_virus_-_type_Asia_1|gb|AEB00690.1|:9.25469E-
7)74:0.00128703)80:0.00226906)100:0.0236013)49:0.00452338,(Foot-and-
mouth_disease_virus_-_type_Asia_1|gb|AAQ90285.1|:0.0411001,((Foot-and-
mouth_disease_virus_-_type_Asia_1|gb|ABI93979.1|:0.0156305,(Foot-and-
mouth_disease_virus_-_type_Asia_1|gb|ABI93976.1|:0.00843482,Foot-and-
mouth_disease_virus_-
_type_Asia_1|gb|ABI93977.1|:0.00475502)98:0.00458548)100:0.0200869,(Foot
-and-mouth_disease_virus_-_type_Asia_1|gb|AAT01739.1|:0.026264,Foot-and-
mouth_disease_virus_-
_type_Asia_1|gb|AAT01740.1|:0.0237958)78:0.00541545)98:0.00646944)82:0.0
0620115)25:0.00359053)33:0.00441829,Foot-and-mouth_disease_virus_-
_type_Asia_1|gb|ABI93986.1|:0.0329163)52:0.00493166)100:0.0569904,(((Foot
-and-mouth_disease_virus_C4|gb|AAT01751.1|:0.0261135,((Foot-and-
mouth_disease_virus_C1|gb|AAT01747.1|:6.74373E-4,Foot-and-
mouth_disease_virus_C1|gb|AAT01748.1|:6.74112E-4)90:0.00161324,(((Foot-
and-mouth_disease_virus_-_type_C|gb|CAL64901.1|:0.0067818,Foot-and-
mouth_disease_virus|gb|CAL64773.1|:9.25469E-7)95:0.0019529,(((Foot-and-
mouth_disease_virus_-_type_C|gb|ABD67461.1|:0.00610279,(Foot-and-
mouth_disease_virus_-_type_C|gb|ABD67456.1|:0.00442346,Foot-and-
mouth_disease_virus_-
_type_C|gb|ABD67458.1|:0.00202683)66:0.00203104)47:0.0013098,(Foot-and-
mouth_disease_virus_-_type_C|gb|ABD67457.1|:0.00196293,Foot-and-
mouth_disease_virus_-
_type_C|gb|ABD67460.1|:0.00198256)79:0.00301476)90:0.00425557,(Foot-
and-mouth_disease_virus_-_type_C|gb|ACO40496.1|:9.25469E-7,(Foot-and-
mouth_disease_virus_-_type_C|gb|ABD67453.1|:0.00146385,Foot-and-
mouth_disease_virus_-
_type_C|gb|ABD67455.1|:0.00135707)48:0.00135374)29:8.04443E-
4)98:0.00341188)100:0.00550094,(Foot-and-mouth_disease_virus_-
_type_C|gb|CAB60267.1|:9.25469E-7,((Foot-and-mouth_disease_virus_-
_type_C|gb|CAB60265.1|:9.25469E-7,Foot-and-mouth_disease_virus_-
_type_C|gb|CAB60266.1|:0.00202988)100:0.00677688,(Foot-and-
mouth_disease_virus|gb|CAM73960.1|:0.00134869,Foot-and-
mouth_disease_virus|gb|CAM73961.1|:9.25469E-

7)96:0.00405944)66:6.74808E-4)77:5.29276E-4)100:0.00873802)100:0.0186749)88:0.00928839,(Foot-and-mouth_disease_virus_-_type_C|gb|AAT01753.1|:0.0393348,(Foot-and-mouth_disease_virus_C3|gb|AAT01750.1|:0.00555485,Foot-and-mouth_disease_virus_C5|gb|AAT01752.1|:0.0115681)100:0.0140379)39:0.00260811)82:0.00729472,(Foot-and-mouth_disease_virus_C3|gb|AAT01749.1|:0.0138826,(Foot-and-mouth_disease_virus|gb|CAA07472.1|:9.25469E-7,Foot-and-mouth_disease_virus|gb|CAA07561.1|:0.00135239)100:0.028193)98:0.00725539)100:0.0558865)56:0.0170583,((((((Foot-and-mouth_disease_virus_-_type_O|gb|AHE63362.1|:0.0174295,Foot-and-mouth_disease_virus_-_type_O|gb|AHK60405.1|:0.0141117)69:0.00513622,(((((Foot-and-mouth_disease_virus_-_type_O|gb|AFZ77002.1|:0.00222844,(Foot-and-mouth_disease_virus_-_type_O|gb|AFZ77041.1|:0.00112276,((Foot-and-mouth_disease_virus_-_type_O|gb|AFZ77042.1|:6.7016E-4,Foot-and-mouth_disease_virus_-_type_O|gb|AFZ77045.1|:6.69211E-4)90:0.00134247,(Foot-and-mouth_disease_virus_-_type_O|gb|AFZ77043.1|:6.69061E-4,Foot-and-mouth_disease_virus_-_type_O|gb|AFZ77044.1|:9.25469E-7)53:9.25469E-7)84:8.84692E-4)72:0.0020091)100:0.00448437,Foot-and-mouth_disease_virus_-_type_O|gb|AFZ77049.1|:6.70565E-4)51:6.6905E-4,((((Foot-and-mouth_disease_virus_-_type_O|gb|AFZ77054.1|:0.00201108,Foot-and-mouth_disease_virus_-_type_O|gb|AFZ77055.1|:0.00268374)91:0.00201124,Foot-and-mouth_disease_virus_-_type_O|gb|AFZ77048.1|:0.00268178)17:9.25469E-7,(Foot-and-mouth_disease_virus_-_type_O|gb|AFZ77053.1|:0.00201092,Foot-and-mouth_disease_virus_-_type_O|gb|AFZ77050.1|:0.00268286)11:9.25469E-7)2:9.25469E-7,(Foot-and-mouth_disease_virus_-_type_O|gb|AFZ77052.1|:0.00201049,Foot-and-mouth_disease_virus_-_type_O|gb|AFZ77046.1|:0.00133939)13:9.25469E-7)33:9.25469E-7)30:9.25469E-7,Foot-and-mouth_disease_virus_-_type_O|gb|AFZ77051.1|:0.002685)100:0.0166406,((Foot-and-mouth_disease_virus_-_type_O|gb|AGZ15284.1|:0.0137615,((Foot-and-mouth_disease_virus_-_type_O|gb|ADR51745.1|:0.002019,(Foot-and-mouth_disease_virus_-_type_O|gb|ADR51743.1|:0.0013408,Foot-and-mouth_disease_virus_-_type_O|gb|ADR51744.1|:9.25469E-7)100:0.00336261)57:7.24089E-4,(Foot-and-mouth_disease_virus_-_type_O|gb|ADR51746.1|:0.00206481,Foot-and-mouth_disease_virus_-_type_O|gb|ADR51741.1|:0.00534303)47:6.7153E-4)99:0.00530134)97:0.00684089,((Foot-and-mouth_disease_virus_-_type_O|gb|ADR66169.1|:6.73626E-4,Foot-and-mouth_disease_virus_-_type_O|gb|ADR66170.1|:9.25469E-7)100:0.014783,(Foot-and-mouth_disease_virus_-_type_O|gb|AEO16215.1|:0.00421612,(Foot-and-mouth_disease_virus_-_type_O|gb|ADV52245.1|:0.00134576,Foot-and-mouth_disease_virus_-_type_O|gb|AFE84736.1|:0.00134603)100:0.00525771)61:0.00153311)60:0.00155408)94:0.00549142)37:0.00140368)32:0.00343891,Foot-and-mouth_disease_virus_-_type_O|gb|AAT01777.1|:0.0205761)14:9.15195E-4,(Foot-and-mouth_disease_virus_-_type_O|gb|AAT01755.1|:0.02381,(Foot-and-

mouth_disease_virus_-_type_O|gb|BAC06475.1|:0.00949994,(Foot-and-
mouth_disease_virus_-_type_O|gb|AHC06115.1|:0.00364682,(Foot-and-
mouth_disease_virus_-_type_O|gb|AAP79123.1|:0.0136937,((Foot-and-
mouth_disease_virus_-_type_O|gb|ABV03521.1|:0.00271227,Foot-and-
mouth_disease_virus_-_type_O|gb|AHC06121.1|:9.25469E-
7)100:0.00742734,((Foot-and-mouth_disease_virus_-
_type_O|gb|AHC06117.1|:9.25469E-7,Foot-and-mouth_disease_virus_-
_type_O|gb|AHC06123.1|:6.74499E-4)67:6.74407E-4,(Foot-and-
mouth_disease_virus_-_type_O|gb|AHC06120.1|:9.25469E-7,(Foot-and-
mouth_disease_virus_-_type_O|gb|AHC06112.1|:6.74768E-4,(Foot-and-
mouth_disease_virus_-_type_O|gb|AHC06118.1|:6.74479E-4,Foot-and-
mouth_disease_virus_-_type_O|gb|AHC06114.1|:6.73646E-4)50:9.25469E-
7)16:9.25469E-7)30:9.25469E-7)90:7.62511E-
4)69:0.00327115)46:0.00203162)100:0.0157791)100:0.0103779)65:0.005601
95)17:0.00290226,((Foot-and-mouth_disease_virus_-
_type_O|gb|ADV52244.1|:0.0270391,((Foot-and-mouth_disease_virus_-
_type_O|gb|AAZ23807.2|:0.0205721,(Foot-and-mouth_disease_virus_-
_type_O|gb|AGO58288.1|:0.0128935,((Foot-and-mouth_disease_virus_-
_type_O|gb|ADH32285.1|:0.00134688,(Foot-and-mouth_disease_virus_-
_type_O|gb|AET43041.1|:0.00676242,(((Foot-and-mouth_disease_virus_-
_type_O|gb|AFN55126.1|:0.00544503,Foot-and-mouth_disease_virus_-
_type_O|gb|AGO58295.1|:0.00542839)94:0.00219591,(Foot-and-
mouth_disease_virus_-_type_O|gb|AGO58298.1|:8.3928E-4,(Foot-and-
mouth_disease_virus_-_type_O|gb|AET43040.1|:0.00473682,(((Foot-and-
mouth_disease_virus_-_type_O|gb|AGG91149.1|:6.74127E-4,(Foot-and-
mouth_disease_virus_-_type_O|gb|AHA36704.1|:0.00337364,Foot-and-
mouth_disease_virus_-_type_O|gb|AGO58296.1|:9.25469E-7)16:9.25469E-
7)99:0.00337269,(Foot-and-mouth_disease_virus_-
_type_O|gb|AGO58297.1|:0.00337381,(Foot-and-mouth_disease_virus_-
_type_O|gb|AGO58292.1|:0.00406132,(Foot-and-mouth_disease_virus_-
_type_O|gb|AHA36705.1|:6.7424E-4,Foot-and-mouth_disease_virus_-
_type_O|gb|AHA36706.1|:0.00338182)96:0.00405078)51:9.25469E-
7)79:6.74057E-4)98:0.00270279,Foot-and-mouth_disease_virus_-
_type_O|gb|AGO58294.1|:0.00543293)74:6.74435E-
4)82:0.00186277)85:0.00270432)65:0.00186202,(Foot-and-
mouth_disease_virus_-_type_O|gb|ADN28046.1|:0.00296516,Foot-and-
mouth_disease_virus_-
_type_O|gb|AFM56034.1|:0.00516593)96:0.00337712)42:9.25469E-
7)14:9.25469E-7)100:0.00472944,Foot-and-mouth_disease_virus_-
_type_O|gb|AGO58289.1|:0.00405072)48:9.25469E-
7)100:0.00956998)100:0.0113368,(Foot-and-mouth_disease_virus_-
_type_O|gb|ADC92546.1|:0.0184162,(Foot-and-mouth_disease_virus_-
_type_O|gb|ADC92548.1|:0.0204704,(Foot-and-mouth_disease_virus_-
_type_O|gb|ADV52247.1|:0.00667966,(Foot-and-mouth_disease_virus_-
_type_O|gb|ADH32284.1|:0.00545014,(Foot-and-mouth_disease_virus_-
_type_O|gb|ADH32283.1|:0.00203333,(Foot-and-mouth_disease_virus_-
_type_O|gb|AGO58290.1|:0.00545001,Foot-and-mouth_disease_virus_-
_type_O|gb|AGO58293.1|:0.0047694)87:0.0020406)56:9.25469E-
7)100:0.00491143)98:0.00836483)55:0.0044977)82:0.00509666)81:0.005858

87)100:0.011869,Foot-and-mouth_disease_virus_-
_type_O|gb|AAT01771.1|:0.0122428)28:0.00214507)33:0.00387734,((((Foot-
and-mouth_disease_virus_O/HK/2001|gb|ABY91241.1|:0.010452,(Foot-and-
mouth_disease_virus_-_type_O|gb|ABM63320.1|:0.00588751,Foot-and-
mouth_disease_virus_-
_type_O|gb|ABB69024.1|:0.00501222)100:0.00708805)99:0.00822628,(Foot-
and-mouth_disease_virus_O/ES/2001|gb|AAT97073.1|:0.0232352,(Foot-and-
mouth_disease_virus_-_type_O|gb|AAT01776.1|:0.00340828,((Foot-and-
mouth_disease_virus_-_type_O|gb|AAG45408.1|:0.0013606,Foot-and-
mouth_disease_virus_-_type_O|gb|AAT01778.1|:9.25469E-
7)67:0.00136016,(Foot-and-
mouth_disease_virus|gb|AAD38188.1|:0.00340361,Foot-and-
mouth_disease_virus_-_type_O|gb|AAB81991.2|:0.0195908)38:9.25469E-
7)18:9.25469E-7)100:0.00973707)64:0.00445435)100:0.0668597,(Foot-and-
mouth_disease_virus_-_type_O|gb|AAT01756.1|:0.0571422,(Foot-and-
mouth_disease_virus_-_type_O|gb|AAQ55600.1|:0.0157136,(Foot-and-
mouth_disease_virus_-_type_O|gb|AAM44304.1|:0.0107976,(Foot-and-
mouth_disease_virus_-_type_O|gb|ABF00113.1|:0.00668373,Foot-and-
mouth_disease_virus_-
_type_O|gb|ABF00114.1|:0.00690774)100:0.0231257)93:0.0107918)97:0.0097
674)40:0.00672388)22:0.00669211,((Foot-and-mouth_disease_virus_-
_type_O|gb|ACL52158.1|:0.00480364,(Foot-and-mouth_disease_virus_-
_type_O|gb|ACL52157.1|:0.0153935,(Foot-and-mouth_disease_virus_-
_type_O|gb|ABR19839.1|:0.00517284,Foot-and-mouth_disease_virus_-
_type_O|gb|ADM36039.1|:0.00302127)100:0.0111558)60:0.00472486)100:0.01
97643,(((Foot-and-mouth_disease_virus_-
_type_O|gb|AAT01772.1|:0.0434639,(Foot-and-mouth_disease_virus_-
_type_O|gb|AAT01765.1|:0.012405,Foot-and-mouth_disease_virus_-
_type_O|gb|AAT01769.1|:0.0151455)100:0.016326)92:0.00894651,Foot-and-
mouth_disease_virus_-
_type_O|gb|AAT01764.1|:0.0215329)48:0.00403196,((((Foot-and-
mouth_disease_virus_-_type_O|gb|AAT01762.1|:0.0013424,(Foot-and-
mouth_disease_virus_-_type_O|gb|CAC86575.1|:6.70495E-4,Foot-and-
mouth_disease_virus_-_type_O|gb|AAT01761.1|:9.25469E-
7)100:0.00402794)37:9.25469E-7,(((Foot-and-mouth_disease_virus_-
_type_O|gb|AFZ75241.1|:0.00201397,Foot-and-mouth_disease_virus_-
_type_O|gb|AFZ75237.1|:0.00268823)14:9.25469E-7,((((Foot-and-
mouth_disease_virus_-_type_O|gb|AAT01758.1|:6.70293E-4,(Foot-and-
mouth_disease_virus_-_type_O|gb|ACC63448.1|:9.25469E-7,Foot-and-
mouth_disease_virus_-_type_O|gb|AAT01759.1|:6.70344E-4)51:6.70388E-
4)42:9.25469E-7,(Foot-and-mouth_disease_virus_-
_type_O|gb|AFZ75240.1|:6.70435E-4,Foot-and-mouth_disease_virus_-
_type_O|gb|AFZ75239.1|:0.0013411)5:9.25469E-7)0:9.25469E-7,Foot-and-
mouth_disease_virus_-_type_O|gb|AFZ75242.1|:9.25469E-7)0:9.25469E-
7,((Foot-and-mouth_disease_virus_-_type_O|gb|ACC63458.1|:9.25469E-
7,((((((Foot-and-mouth_disease_virus_-_type_O|gb|ACC63460.1|:9.25469E-
7,Foot-and-mouth_disease_virus_-_type_O|gb|ACC63461.1|:6.70933E-
4)71:6.70799E-4,Foot-and-mouth_disease_virus_-
_type_O|gb|ACC63459.1|:0.00134461)23:9.25469E-7,(Foot-and-

mouth_disease_virus_-_type_O|gb|ACC63450.1|:9.25469E-7,(Foot-and-
mouth_disease_virus_-_type_O|gb|ACC63449.1|:9.25469E-7,(Foot-and-
mouth_disease_virus_-_type_O|gb|AFU55210.1|:9.25469E-7,((((Foot-and-
mouth_disease_virus_-_type_O|gb|AFU55213.1|:9.25469E-7,Foot-and-
mouth_disease_virus_-_type_O|gb|AFU55214.1|:6.72723E-
4)78:0.00134306,(Foot-and-mouth_disease_virus_-
_type_O|gb|AFU55211.1|:6.72373E-4,(Foot-and-mouth_disease_virus_-
_type_O|gb|AFU55217.1|:6.70726E-4,Foot-and-mouth_disease_virus_-
_type_O|gb|AFU55216.1|:9.25469E-7)37:9.25469E-7)64:6.709E-4)38:9.25469E-
7,Foot-and-mouth_disease_virus_-_type_O|gb|AFU55209.1|:9.25469E-
7)26:9.25469E-7,Foot-and-mouth_disease_virus_-
_type_O|gb|AFU55201.1|:6.71274E-4)63:9.25469E-7)51:6.7018E-
4)64:6.71016E-4)55:6.70998E-4)4:9.25469E-7,Foot-and-mouth_disease_virus_-
_type_O|gb|AFU55205.1|:9.25469E-7)2:9.25469E-7,Foot-and-
mouth_disease_virus_-_type_O|gb|ACC63457.1|:9.25469E-7)9:9.25469E-7,Foot-
and-mouth_disease_virus_-_type_O|gb|ACC63453.1|:6.70879E-4)41:6.71117E-
4)59:6.70485E-4,Foot-and-mouth_disease_virus_-
_type_O|gb|AFZ75243.1|:6.71519E-4)5:9.25469E-7)5:9.25469E-7)24:9.25469E-
7,Foot-and-mouth_disease_virus_-
_type_O|gb|AFZ75238.1|:0.00402622)79:0.00335694)39:6.79309E-4,(Foot-and-
mouth_disease_virus_-_type_O|gb|AAT01780.1|:0.00203253,(((Foot-and-
mouth_disease_virus_-_type_O|gb|AAT01773.1|:0.00869463,(Foot-and-
mouth_disease_virus_-_type_O|gb|AAT01760.1|:0.00407064,Foot-and-
mouth_disease_virus|gb|CAA25416.1|:0.00804831)58:0.00112647)35:9.56935E
-4,Foot-and-mouth_disease_virus_-
_type_O|gb|AAT01757.1|:0.00386528)30:0.00151431,(Foot-and-
mouth_disease_virus_-_type_O|gb|AFZ75232.1|:0.00805901,Foot-and-
mouth_disease_virus_-_type_O|gb|AAT01763.1|:6.71861E-4)42:6.58882E-
4)0:9.25469E-7)2:6.49018E-4)63:0.00197489,Foot-and-mouth_disease_virus_-
_type_O|gb|AFZ75233.1|:0.0094958)100:0.010869)94:0.0153614)19:0.0035918
1)25:0.00427833)100:0.046946)49:0.0196831)99:0.0136755,(Foot-and-
mouth_disease_virus_-_type_A|gb|AAT01698.1|:0.0475072,((((Foot-and-
mouth_disease_virus_-_type_A|gb|AEQ49433.1|:0.0131746,(Foot-and-
mouth_disease_virus_-_type_A|gb|ABU87557.1|:0.0060221,(Foot-and-
mouth_disease_virus_-_type_A|gb|ABU87556.1|:0.00744293,(Foot-and-
mouth_disease_virus_-_type_A|gb|ABU87555.1|:0.00401281,Foot-and-
mouth_disease_virus_-
_type_A|gb|AFE84725.1|:0.00871589)89:0.00133234)97:0.00335181)100:0.010
2884)100:0.0213622,(((Foot-and-mouth_disease_virus_-
_type_A|gb|ADM16569.1|:0.0153162,(Foot-and-mouth_disease_virus_-
_type_A|gb|ADM16568.1|:0.00907388,Foot-and-mouth_disease_virus_-
_type_A|gb|AEE65037.1|:0.0118098)100:0.0201489)86:0.00711409,Foot-and-
mouth_disease_virus_-
_type_A|gb|AEE65036.1|:0.0213395)65:0.00536642,(Foot-and-
mouth_disease_virus_-_type_A|gb|ADM16567.1|:0.0319395,(Foot-and-
mouth_disease_virus_-_type_A|gb|AEO16200.1|:0.0188994,(Foot-and-
mouth_disease_virus_-_type_A|gb|ADV52248.1|:0.0104528,((Foot-and-
mouth_disease_virus_-_type_A|gb|ACX54404.1|:0.00403318,(Foot-and-
mouth_disease_virus_-_type_A|gb|ACX54400.1|:0.00201653,Foot-and-

mouth_disease_virus_-_type_A|gb|ACX54401.1|:6.71225E-4)56:6.70745E-
4)48:6.3871E-4,((Foot-and-mouth_disease_virus_-
_type_A|gb|ACX54402.1|:0.00201592,Foot-and-mouth_disease_virus_-
_type_A|gb|ACX54405.1|:0.0033597)32:9.25469E-7,Foot-and-
mouth_disease_virus_-_type_A|gb|ACX54403.1|:0.00337253)41:7.10973E-
4)100:0.0124015)75:0.00559868)100:0.030743)39:0.00511655)31:0.0034407
4)43:0.00592602,((Foot-and-mouth_disease_virus_-
_type_A|gb|AFE84732.1|:0.0301319,Foot-and-mouth_disease_virus_-
_type_A|gb|AAT01734.1|:0.0149777)99:0.013974,(((Foot-and-
mouth_disease_virus_-_type_A|gb|AEE65042.1|:0.0127101,(Foot-and-
mouth_disease_virus_-_type_A|gb|AEE65041.1|:0.0157736,(Foot-and-
mouth_disease_virus_-_type_A|gb|AEE65039.1|:0.00330184,(Foot-and-
mouth_disease_virus_-_type_A|gb|AEE65038.1|:0.0121427,Foot-and-
mouth_disease_virus_-
_type_A|gb|AEE65040.1|:0.00751393)55:0.00134577)50:6.68758E-
4)55:0.00276023)98:0.0102441,(Foot-and-mouth_disease_virus_-
_type_A|gb|AEE65052.1|:0.018888,(Foot-and-mouth_disease_virus_-
_type_A|gb|AEE65050.1|:0.00536447,Foot-and-mouth_disease_virus_-
_type_A|gb|AEE65051.1|:0.011582)97:0.00587332)98:0.00850627)78:0.006044
98,(Foot-and-mouth_disease_virus_-_type_A|gb|AEE65047.1|:0.0313545,((Foot-
and-mouth_disease_virus_-_type_A|gb|AEE65044.1|:0.034034,Foot-and-
mouth_disease_virus_-_type_A|gb|AEE65049.1|:0.026829)96:0.00996787,Foot-
and-mouth_disease_virus_-
_type_A|gb|AEE65046.1|:0.0295527)18:0.00116959)65:0.00538304)89:0.01022
28)46:0.00382661)44:0.00819685,((Foot-and-mouth_disease_virus_-
_type_A|gb|AFE84727.1|:0.0519118,(((Foot-and-mouth_disease_virus_-
_type_A|gb|AAT01736.1|:0.0314886,(((Foot-and-mouth_disease_virus_-
_type_A|gb|AAT01713.1|:0.0340098,Foot-and-mouth_disease_virus_-
_type_A|gb|AAT01699.1|:0.039518):0.00659685,(Foot-and-
mouth_disease_virus_-_type_A|gb|AAT01746.1|:0.0129215,(Foot-and-
mouth_disease_virus_-_type_A|gb|AAT01730.1|:0.0037508,Foot-and-
mouth_disease_virus_-
_type_A|gb|AAT01731.1|:0.00922)99:0.00853993)100:0.0127863)67:0.002331
99,Foot-and-mouth_disease_virus_-
_type_A|gb|AAT01718.1|:0.0249127)26:0.00393312)36:0.00564127,((((Foot-
and-mouth_disease_virus_-_type_A|gb|AAT01714.1|:0.016572,(Foot-and-
mouth_disease_virus_-_type_A|gb|AAT01703.1|:0.0192091,(Foot-and-
mouth_disease_virus_-_type_A|gb|AAT01710.1|:0.0144067,(Foot-and-
mouth_disease_virus_-_type_A|gb|AAT01735.1|:0.00757233,(Foot-and-
mouth_disease_virus_-_type_A|gb|AAT01724.1|:0.00670349,(Foot-and-
mouth_disease_virus_-_type_A|gb|AAT01723.1|:0.00417452,(Foot-and-
mouth_disease_virus_-_type_A|gb|AAT01697.1|:2.21186E-4,Foot-and-
mouth_disease_virus_-
_type_A|gb|AAT01721.1|:0.0011338)100:0.0130811)73:0.00268179)100:0.003
51334)87:0.00348201)54:9.80062E-4)96:0.00595625)98:0.00796239,(Foot-
and-mouth_disease_virus_-_type_A|gb|AAT01719.1|:0.0173033,(Foot-and-
mouth_disease_virus_-_type_A|gb|AAT01694.1|:0.0221395,(Foot-and-
mouth_disease_virus_-_type_A|gb|AAT01717.1|:0.00201428,(Foot-and-
mouth_disease_virus_-_type_A|gb|AAT01720.1|:6.73449E-4,Foot-and-

mouth_disease_virus_-_type_A|gb|AAT01722.1|:9.25469E-
7)96:0.00135389)100:0.012925)82:0.00764239)99:0.0125998)46:0.00532106,
(Foot-and-mouth_disease_virus_-_type_A|gb|AAT01737.1|:0.0345944,Foot-and-
mouth_disease_virus_-
_type_A|gb|AAT01711.1|:0.0198233)52:0.0101684)7:0.00316265,(Foot-and-
mouth_disease_virus_-_type_A|gb|AAT01700.1|:0.00874323,(Foot-and-
mouth_disease_virus_-_type_A|gb|AAT01696.1|:6.77535E-4,Foot-and-
mouth_disease_virus_-_type_A|gb|AAT01701.1|:9.25469E-
7)100:0.0185561)100:0.0422404)11:0.00316988)29:0.00348128,Foot-and-
mouth_disease_virus_-
_type_A|gb|AAT01725.1|:0.0530232)32:0.0101336)92:0.00725941,Foot-and-
mouth_disease_virus_-
_type_A|gb|AAT01709.1|:0.0366318)44:0.0106805)64:0.00815562)48:0.01367
55);
